# Supplementary material for: State transitions in the statistically stable place cell population correspond to rate of perceptual change
Source: Curr Biol. 2022 Aug 22;32(16):3505–3514.e7. doi: 10.1016/j.cub.2022.06.046 (PMC9616721; doi:10.1016/j.cub.2022.06.046)
Supplement: Document S1. Figures S1–S5 and Table S1 [file mmc1.pdf]

Current Biology, Volume *32*

Supplemental Information

State transitions in the statistically stable place cell population correspond to rate of perceptual change

Sander Tanni, William de Cothi, and Caswell Barry

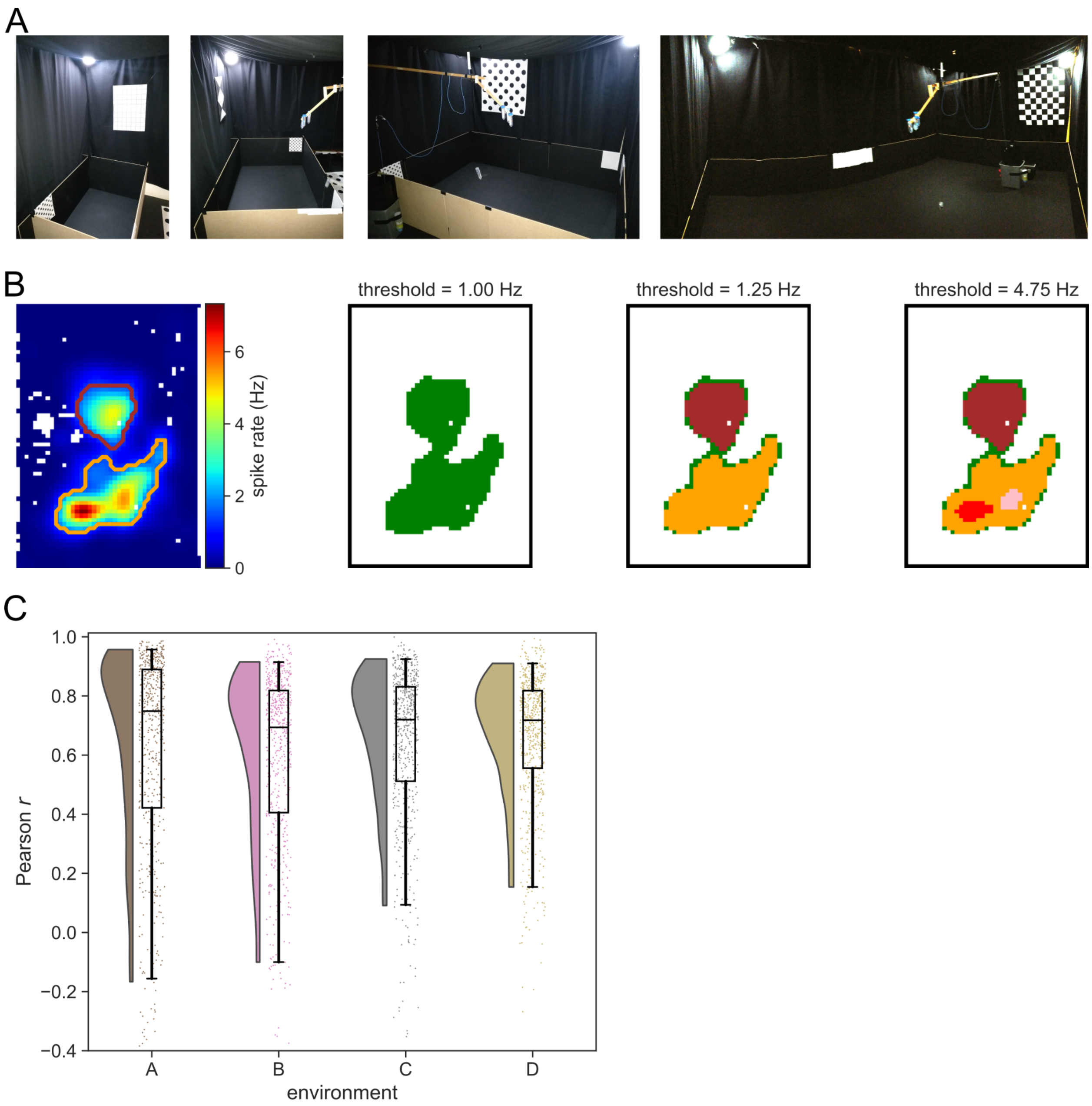

Figure S1. Experimental paradigm description and validation. Related to main Figure 1. (**A**) Photographs of experimental environments (from left: A, B, C and D). (**B**) Field detection method based on iterative thresholding identifies multiple place fields in a single ratemap. The spatial ratemap of a place cell is shown on the left. With the threshold at 1 Hz, only one place field (green) is detected. While increasing the threshold at 0.05 Hz increments, two place fields (brown and orange) are identified with 1.25 Hz threshold. These both pass the place field criteria. By increasing the threshold further, two smaller place fields (red and pink) are detected with a 4.75 Hz threshold, both overlapping with the larger orange place field. At least one of the smaller place fields (red and pink) did not pass the place field criteria. Therefore, both of them (red and pink) were ignored because a larger place field, detected with a lower threshold and overlapping with them, did pass the place field criteria. (**C**) Spatial correlation between first and second half of the recording in each environment for every active place cell. The box shows quartiles of the dataset, and whiskers indicate the 5th and 95th percentile of the data distribution.

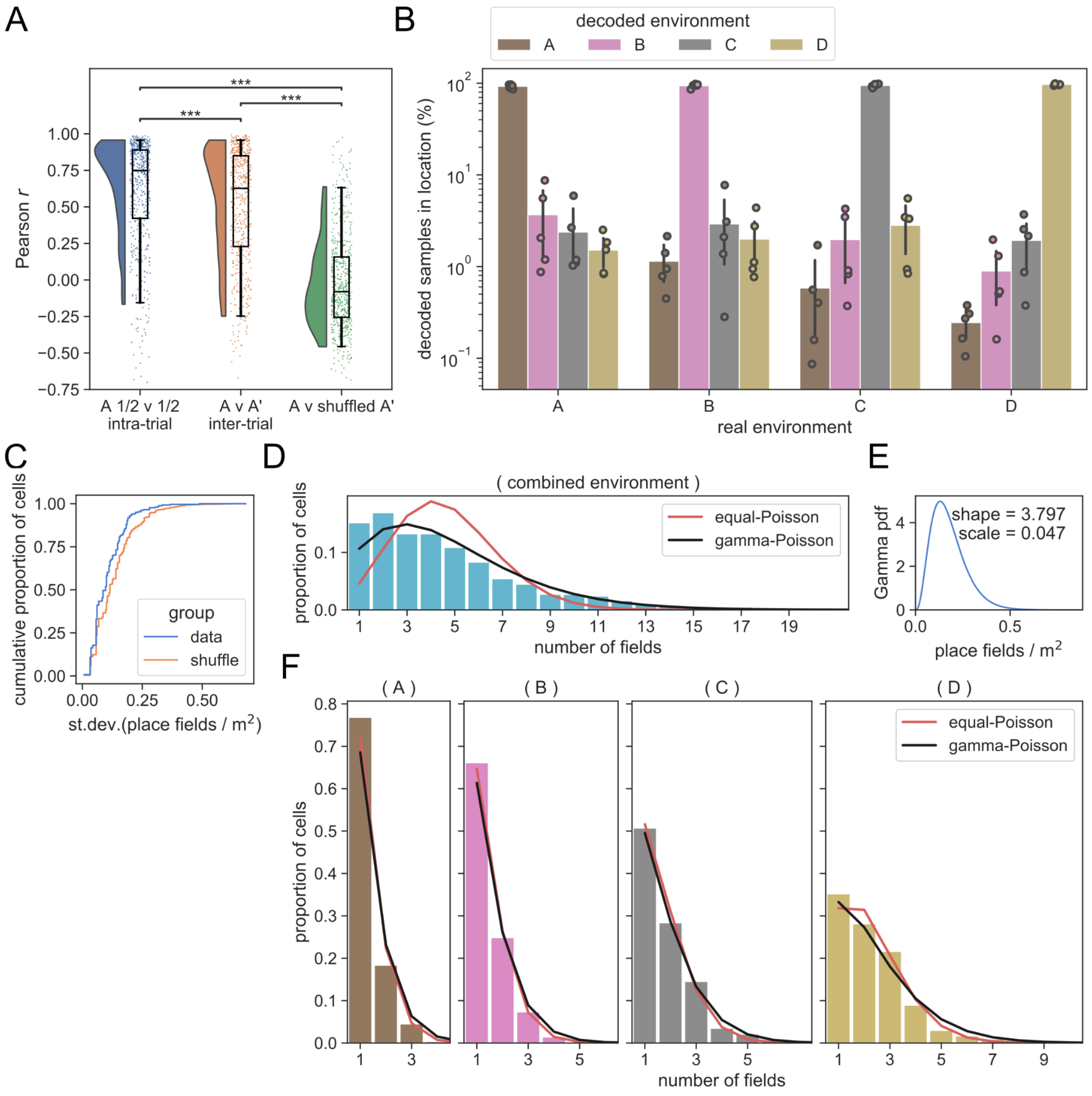

Figure S2. Details of recorded place cell and place field counts. Related to main Figure 2. (**A**) Place cells formed stable representations. Spatial correlations (bin-wise Pearson correlation) between ratemaps from the first and second half of environment A (A ½ v ½) and between repeated trials (A v A') were high (mean spatial correlation of 0.61 and 0.51, respectively), significantly exceeding the values obtained by randomly repairing cells (A v shuffled A') (-0.04). Kruskal-Wallis test: H = 644, $p = 1.64 \times 10^{-140}$; Mann-Whitney for A ½ v ½ and A v A', $U = 1.8 \times 10^{5}$, $p = 3.2 \times 10^{-6}$, for A ½ v ½ and A v shuffled A', $U = 2.8 \times 10^{5}$, $p = 10^{-92}$, for A v A' and A v shuffled A', $U = 2.9 \times 10^{5}$, $p = 10^{-114}$. The box shows quartiles of the dataset, and whiskers indicate the 5th and 95th percentile of the data distribution. P-value markers: ***, 0.001. The kernel-density estimate is bounded between the 5th and 95th percentile. (**B**) Place cells formed distinct representations for each environment. Population activity vectors were reliably decoded to the environment from which they were drawn - Bayesian-framework with 1s window used for decoding. Error bars show 95% confidence intervals of the mean based on bootstrapping. (**C**) Place cell field formation propensity is conserved across environments. The standard deviation of place cells' field formation propensity (place fields / m^2, accounting for field density in

Figure 2E inset) across environments was lower than for a shuffled distribution (Mann-Whitney: $U = 2.7 \times 10^7$; $p = 6 \times 10^{-8}$; $n = 258$). Only cells with at least 1 field in each environment were used in this analysis. Shuffle was obtained by permuting cell identities within each animal and environment 1000 times. (**D**) Distribution of field counts per place cell after grouping all environments together and predictions of the two models fit to this data. (**E**) Probability density function (pdf) of gamma with fitted parameters, defining the field propensity distribution as a function of environment size. (**F**) Distribution of field counts per place cell for cells in each environment that have at least one place field, and predictions of the two models fit to data in **D**.

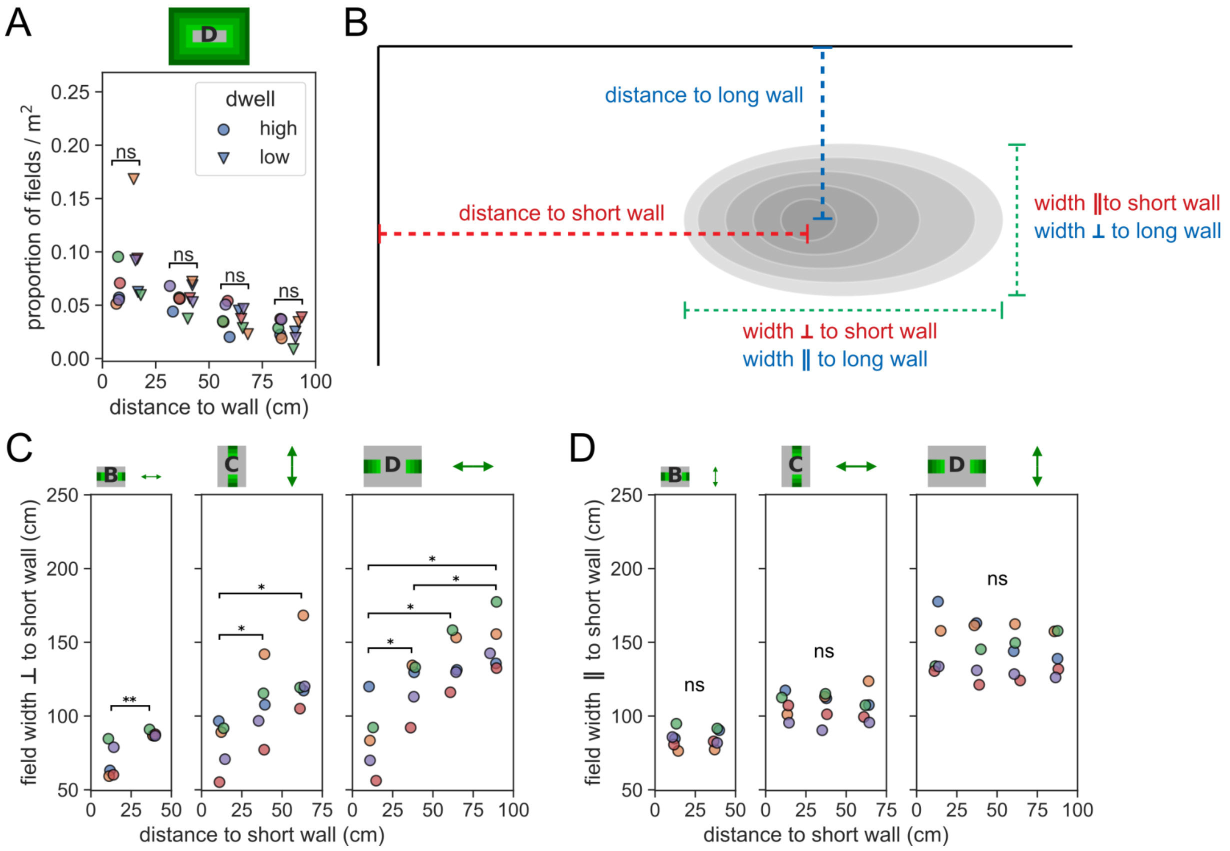

Figure S3. Variability in place field density and size. Related to main Figure 3. (**A**) Change in field density with distance to wall is not explained by the difference in dwell time. Field density (proportion of fields / m^2) and dwell time were computed for 25 x 25 cm non-overlapping regions of the largest environment chosen to be at a range of distances to the nearest wall. For each animal, we grouped regions according to distance to the wall and for each of these groups found the mean field density in the region with the highest and lowest dwell time. Field density did not vary with dwell time (Mann-Whitney for all comparisons: $U \geq 6$, $p \geq 0.11$) but was different between regions at different distances to the wall (Kruskal-Wallis for both high and low dwell: $H \geq 13.1$, $p \leq 0.004$). P-value markers: *, 0.05; **, 0.01; ns, not significant. (**B**) The width of each place field was measured along two orthogonal axes. The distance of the place field from nearest walls in the two axes was measured from the location of peak firing rate. (**C**) The average place field size per animal measured orthogonal to the short wall increases with distance from the wall and is also greater near the wall in the larger environments. Pair-wise post hoc tests adjusted for multiple comparisons using Benjamini/Hochberg (non-negative) correction. The cartoon above the plot indicates the wall distances and included locations in the environment in green, and the arrow indicates the axis of measurement. (**D**) The average place field size per animal measured parallel to the short wall is constant at all distances to the short wall in all environments.

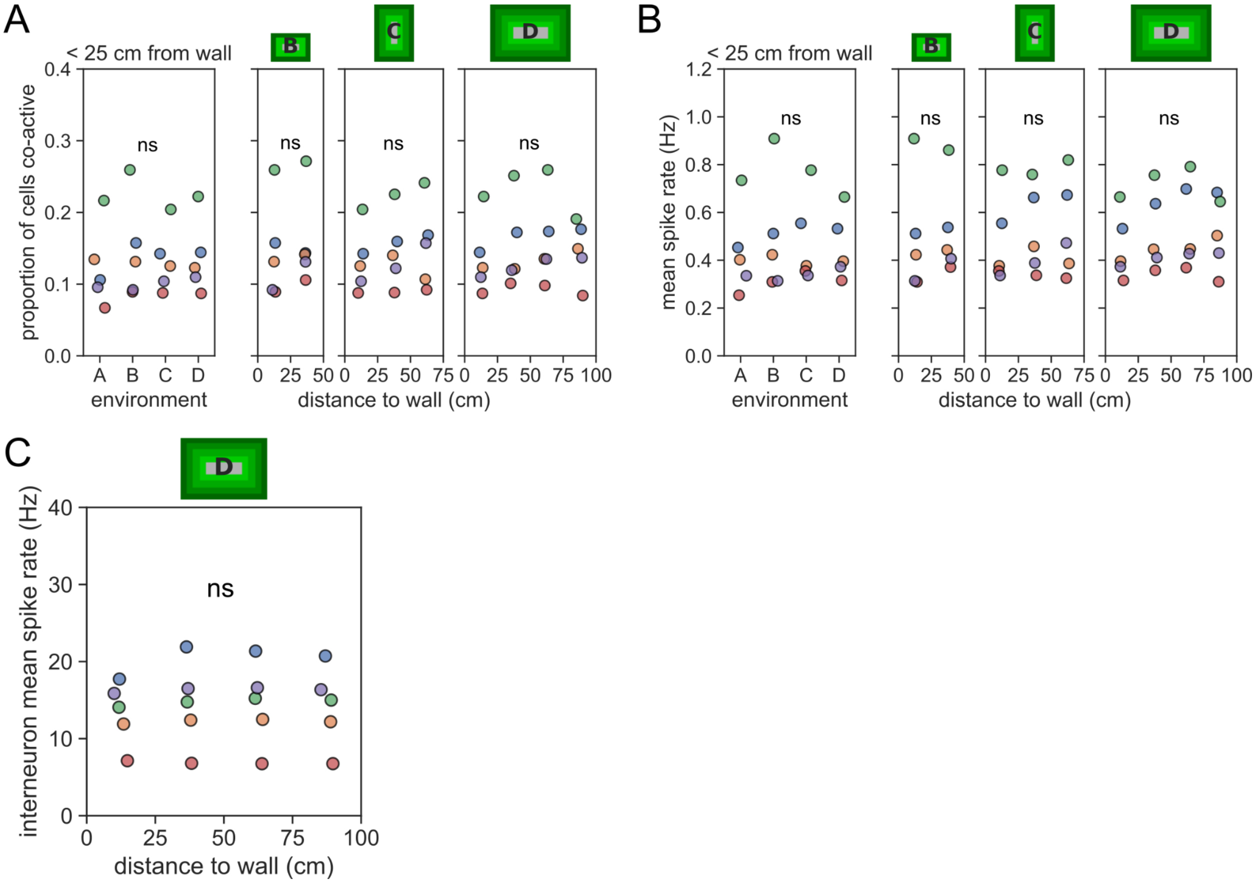

Figure S4. Homeostasis of place cell population activity. Related to main Figure 4. (**A**) The proportion of place cells recorded in each animal that were firing at greater than 1 Hz was constant at different distances to the wall in all environments and same across environments. P-value markers: ns, not significant. (**B**) The mean firing rate of all place cells recorded in each animal was constant at different distances to the wall in all environments and across environments. (**C**) Interneuron firing rate is constant at all distances to walls. Mean spike rate of all interneurons detected in each animal at different distances to wall in environment D.

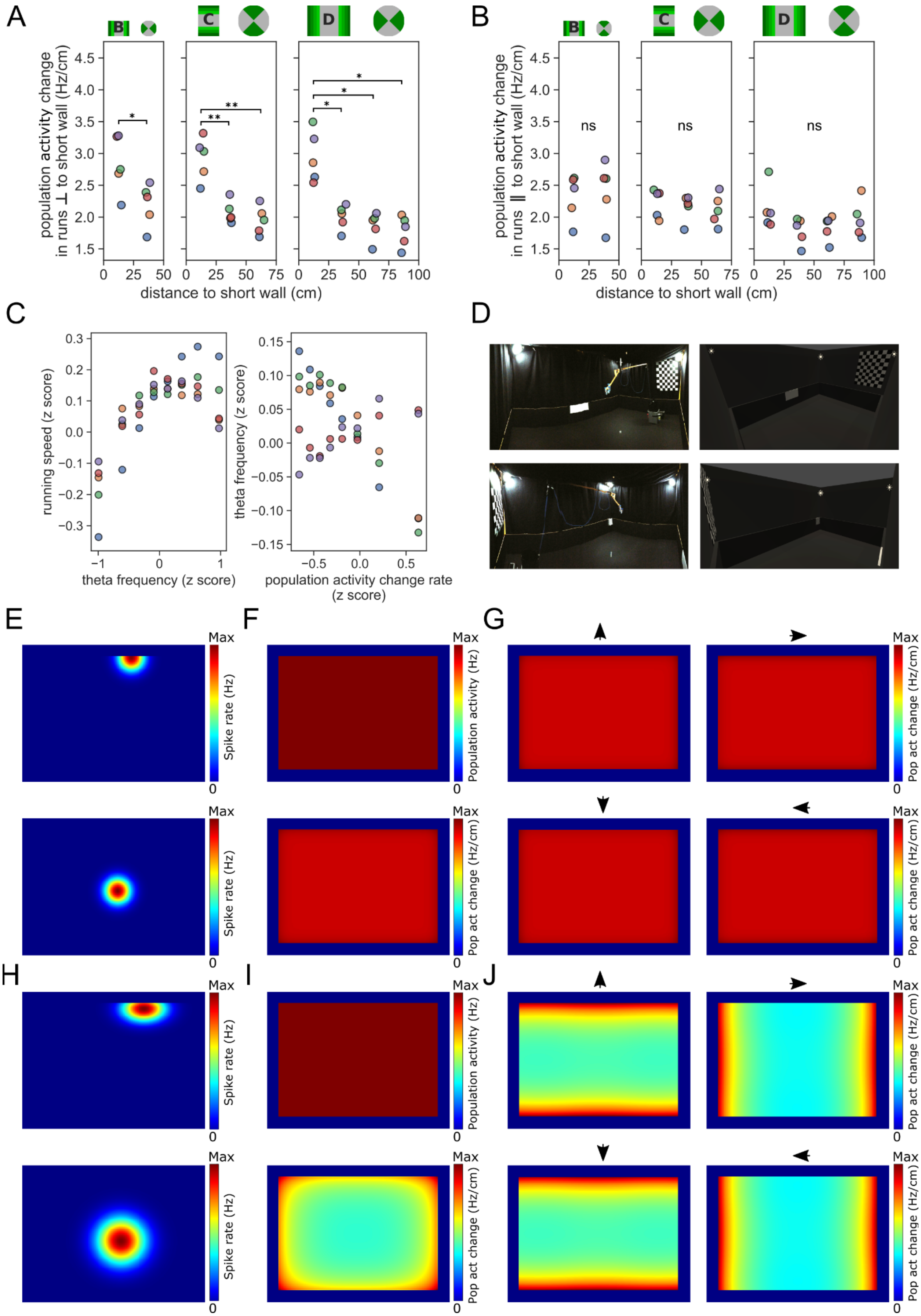

A
B
C
D
population activity change in runs ⊥ to short wall (Hz/cm)
*
**
**
*
*
*
distance to short wall (cm)
population activity change in runs ∥ to short wall (Hz/cm)
ns
ns
ns
running speed (z score)
theta frequency (z score)
theta frequency (z score)
population activity change rate (z score)
E
F
G
H
I
J
Max
0
Spike rate (Hz)
Population activity (Hz)
Pop act change (Hz/cm)

Figure S5. Analysis of population activity change rate. Related to main Figure 5. (**A&B**) Population activity change was higher in runs orthogonal and close to walls in all environments. (**A**) Euclidean distance between vectors representing population activity at different distances to the short walls of each environment, only including runs orthogonal to the short wall. Pair-wise post hoc tests adjusted for multiple comparisons using Benjamini/Hochberg (non-negative) correction. The cartoon above each plot indicates the wall distances and included locations in the environment in green, and the green sectors on the circles indicate the sampled movement directions. P-value markers: *, 0.05; **, 0.01; ns, not significant. (**B**) Same as **A**, but runs parallel to the short wall. (**C**) Running speed (z-scored) was positively correlated with theta frequency (z-scored) (r = 0.558, p = 0.0002) in partial correlation while accounting for population activity change rate (z-scored) in the largest environment, however, theta oscillation frequency was not correlated with population activity change rate, while running speed was accounted for in the partial correlation analysis (r = -0.009, p = 0.96). (**D**) The virtual environment used to calculate visual change. Recording environment D (left) was replicated in a virtual environment (right) in order to estimate each rodent's change in visual scene during its movement through the experimental environment. (**E**) Simulating 1271 equal-sized Gaussian place fields (two examples shown) that evenly span the largest recording environment such that 'true' field peaks can lay outside the environmental boundaries means that (**F** top) the total firing of the place cell population and (**F** bottom) the rate of change in the place cell population are the same at every measurable location, independent of heading direction (**G**). If instead (**H**) the place fields are distributed more densely at boundaries with field widths that increase the further the distance to an orthogonal boundary (two examples shown), we see (**I** top) the total population firing is still the same everywhere but now (**I** bottom) the rate of change in the population activity is increased at the boundaries. In particular, (**J**) this increase at the boundaries is primarily driven when travelling orthogonally to them, as seen in our main results (Fig 5). The simulations can be reproduced using our code shared on GitHub (https://github.com/Barry-lab/Publication_TanniDeCothiBarry2022/tree/main).

Animal	N cells	Mahalanobis distance VS field count	L-ratio VS field count	Mahalanobis distance VS field area	L-ratio VS field area
1	55	r = 0.066, p = 0.630	r = -0.087, p = 0.525	r = -0.079, p = 0.566	r = -0.250, p = 0.065
2	90	r = 0.124, p = 0.245	r = -0.106, p = 0.322	r = -0.002, p = 0.981	r = -0.038, p = 0.722
3	92	r = -0.038, p = 0.719	r = -0.160, p = 0.127	r = -0.026, p = 0.809	r = -0.024, p = 0.822
4	132	r = 0.100, p = 0.254	r = -0.201, **p = 0.021**	r = -0.080, p = 0.361	r = -0.006, p = 0.943
5	132	r = -0.072, p = 0.409	r = -0.050, p = 0.566	r = 0.132, p = 0.130	r = -0.030, p = 0.733

Table S1. Clustering quality does not account for differences in field size and field number between cells. Related to main Figures 2 and 3. Place fields were counted and their average field areas measured for each cell in the largest environment (D), these values were correlated with two measures of cluster goodness - Mahalanobis distance and L-ratio. A single significant correlation was found (in bold), indicating for Animal 4 that better separated cells (L-ratio) tended to have more fields. Since no adjustment for multiple comparisons was applied we believe this single result does not imply a systematic relationship between cluster goodness and field measures.
