# Supplementary material for: State transitions in the statistically stable place cell population correspond to rate of perceptual change
Source: Curr Biol. 2022 Aug 22;32(16):3505–3514.e7. doi: 10.1016/j.cub.2022.06.046 (PMC9616721; doi:10.1016/j.cub.2022.06.046)
Supplement: Data S1. Ratemaps and spike plots of example place cells for unit-level qualitative assessment, related to Figure 1 and population activity change in the STAR Methods — (A) Boundary-visit dependent ratemaps of 50 cells. Ratemaps (left), spike and trajectory plots (middle), and boundary maps (right) of 50 place cells in the largest environment, 10 from each rat. Panels of each cell are separated by horizontal lines. All three panels are in pairs with data split by time into the first and second half of the recording. Boundary maps were created by computing separate ratemaps for each wall (N - north, E - east, S - south, W - west), filtering for periods of the recording where the respective wall was the last one reached by the animal (traversing ≤ 20 from the wall), and merging these four ratemaps into different colour channels (N - red, E - green, S - blue, W - grey). Cross-correlograms between opposing boundary-tethered rate maps did not show a systematic offset (t(99)=1.1, p=0.27). Colour coding of the spikes on the spike and trajectory plots (middle) are indicated above the panel for the first cell. (B) Spike and trajectory plots of 50 place cells. 10 cells per animal in the largest environment. [file mmc2.pdf]

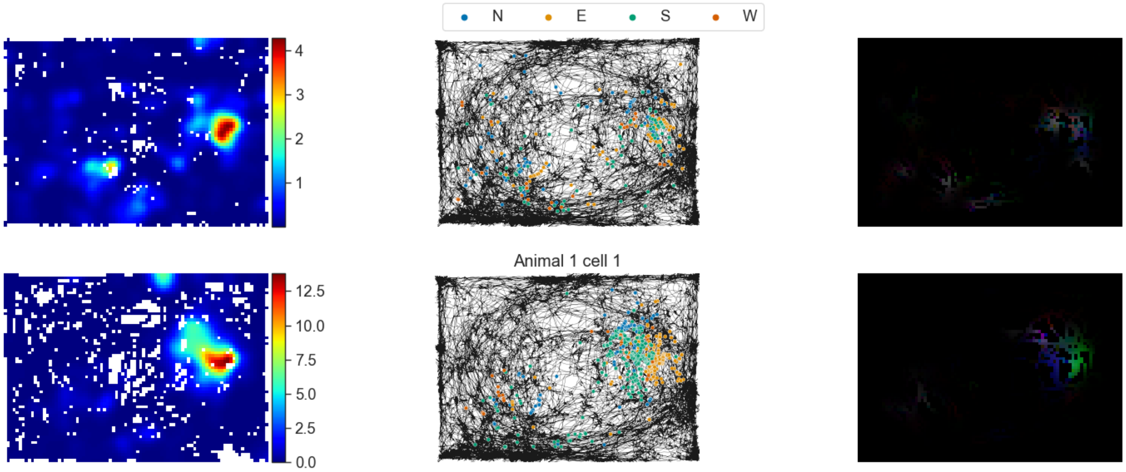
N
E
S
W
Animal 1 cell 1

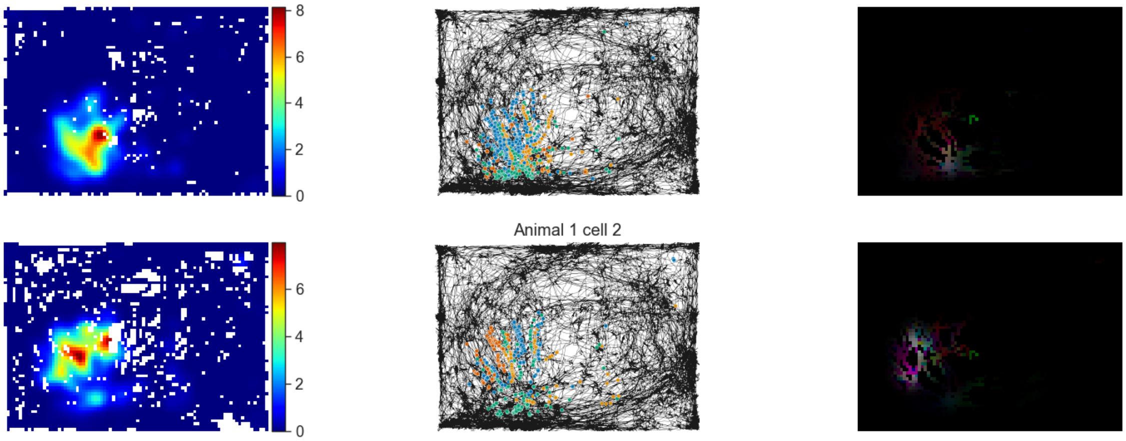
Animal 1 cell 2

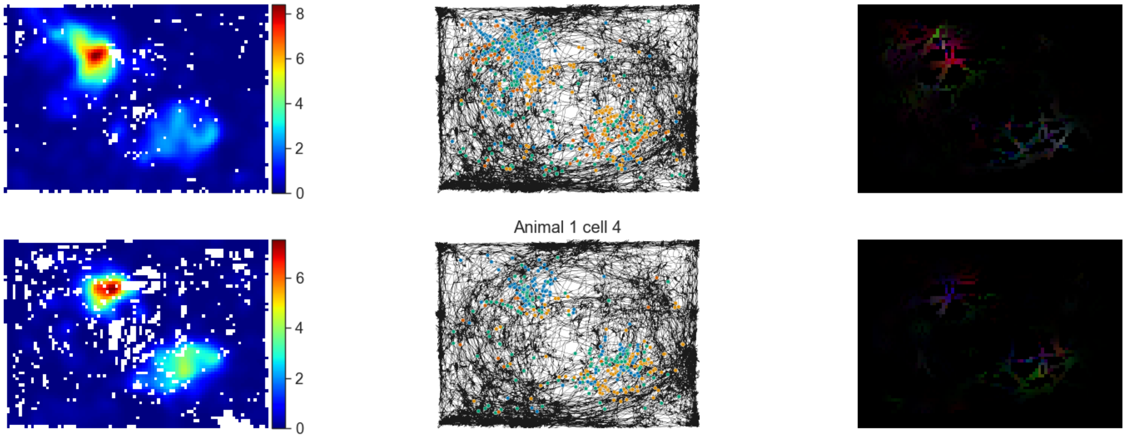
Animal 1 cell 4

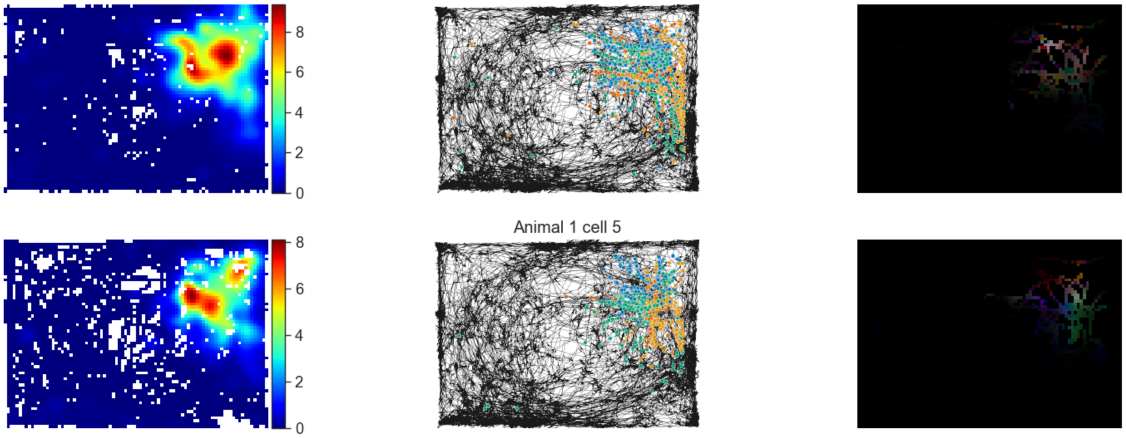
Animal 1 cell 5
0 2 4 6 8
0 2 4 6 8

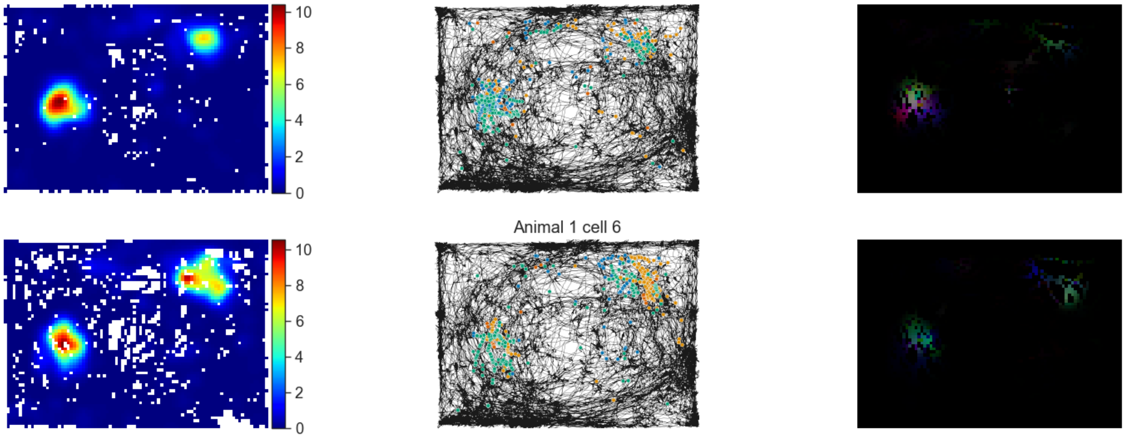
Animal 1 cell 6
0 2 4 6 8 10
0 2 4 6 8 10

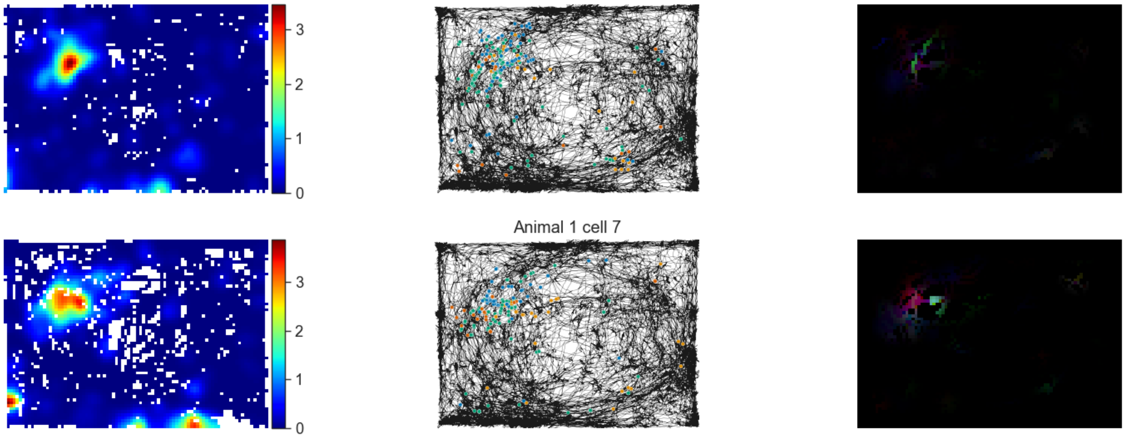
Animal 1 cell 7
0 1 2 3
0 1 2 3

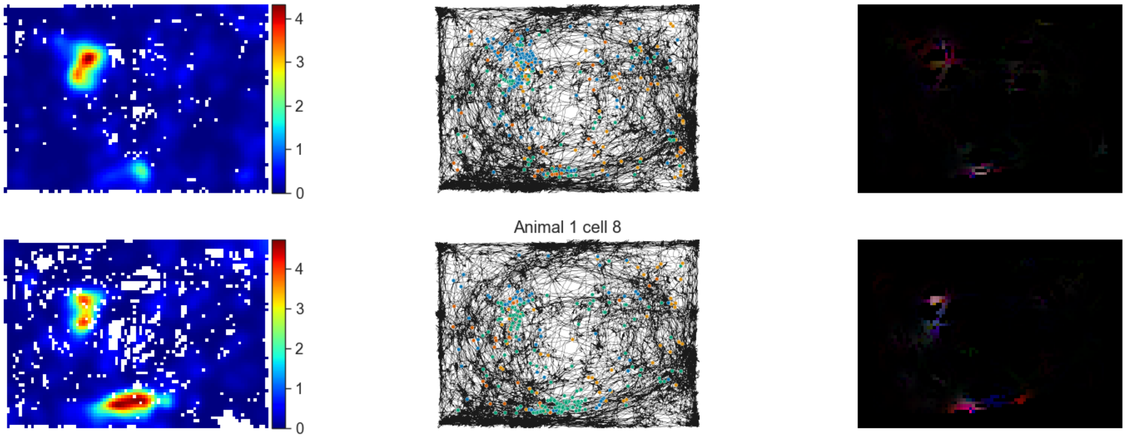
Animal 1 cell 8

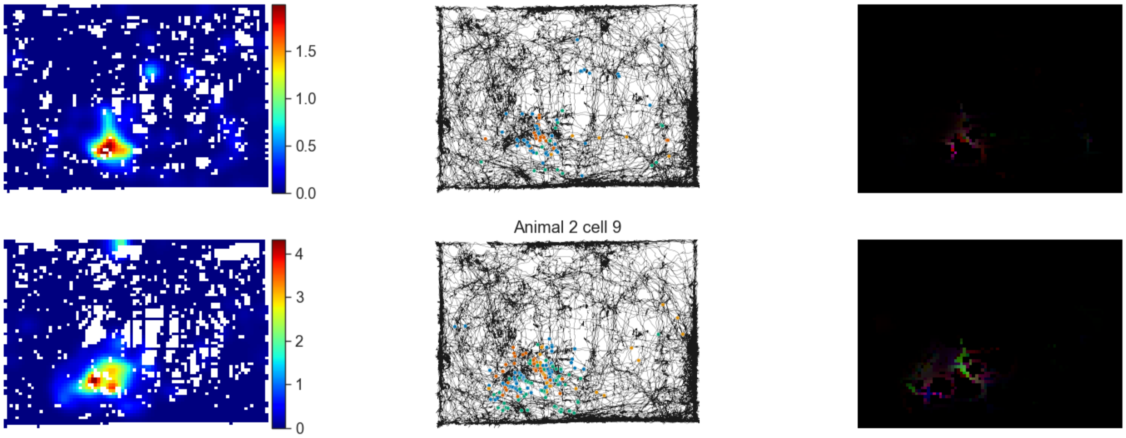
Animal 2 cell 9

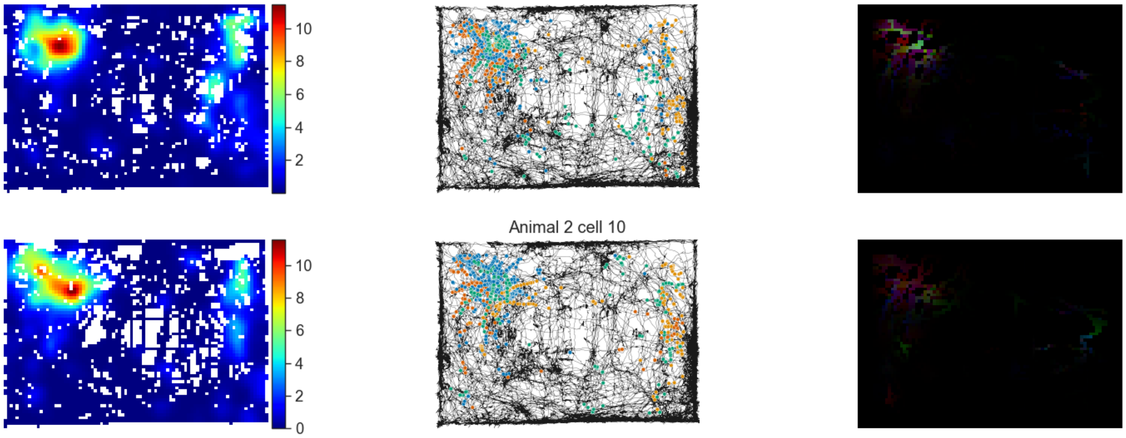
Animal 2 cell 10

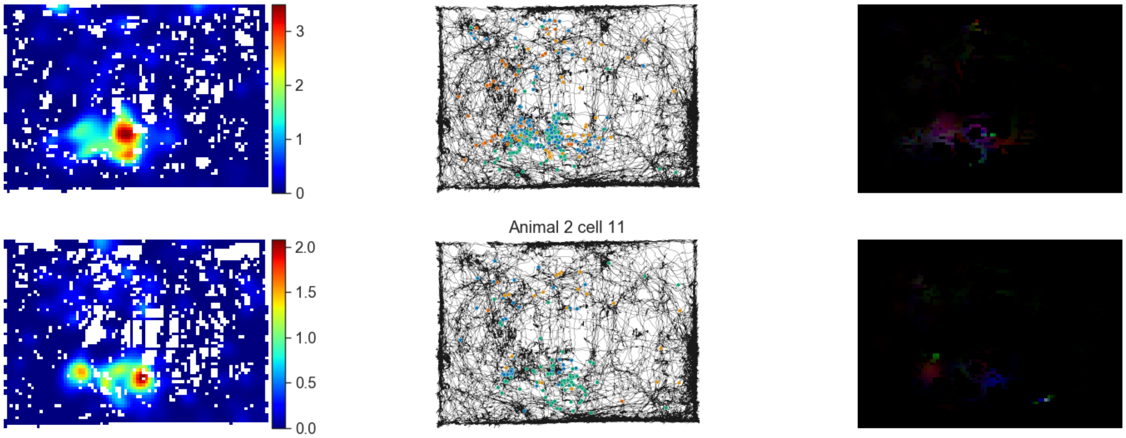
Animal 2 cell 11
0
1
2
3
0.0
0.5
1.0
1.5
2.0

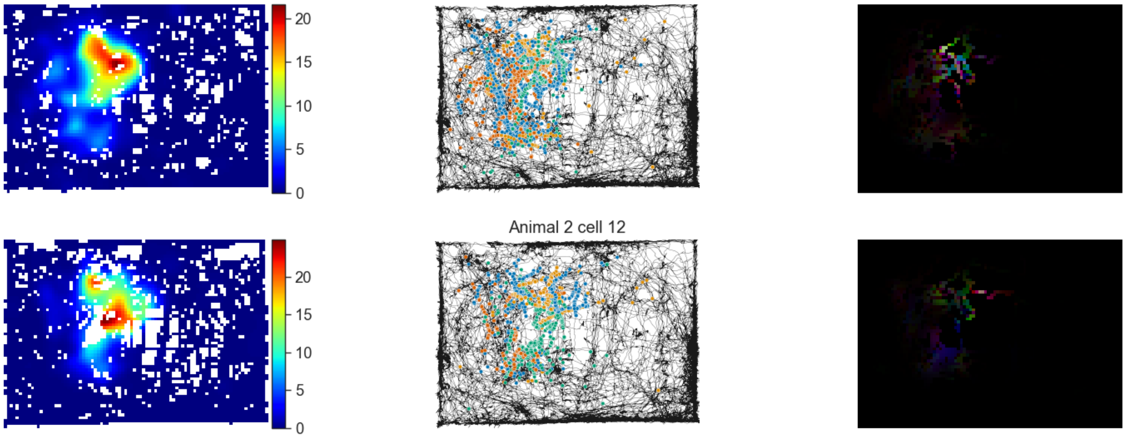
Animal 2 cell 12
0
5
10
15
20
0
5
10
15
20

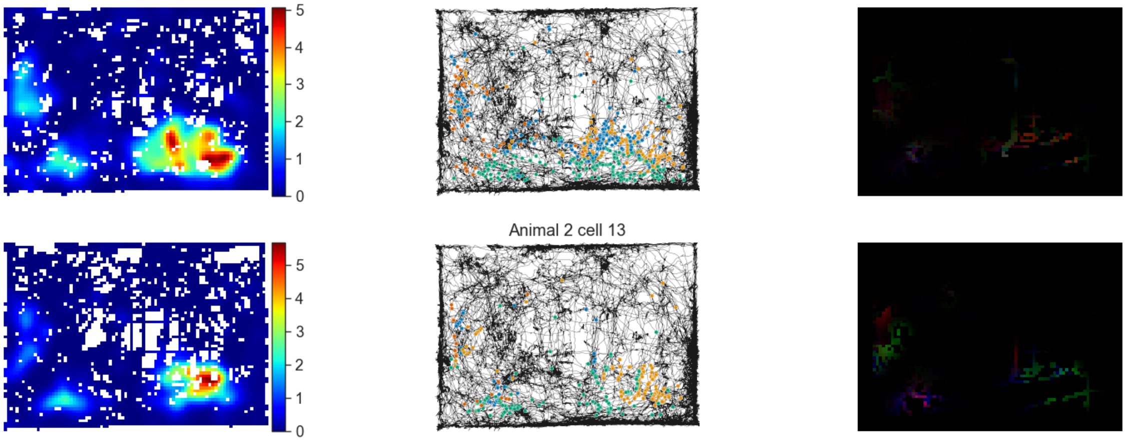
Animal 2 cell 13
0
1
2
3
4
5
0
1
2
3
4
5

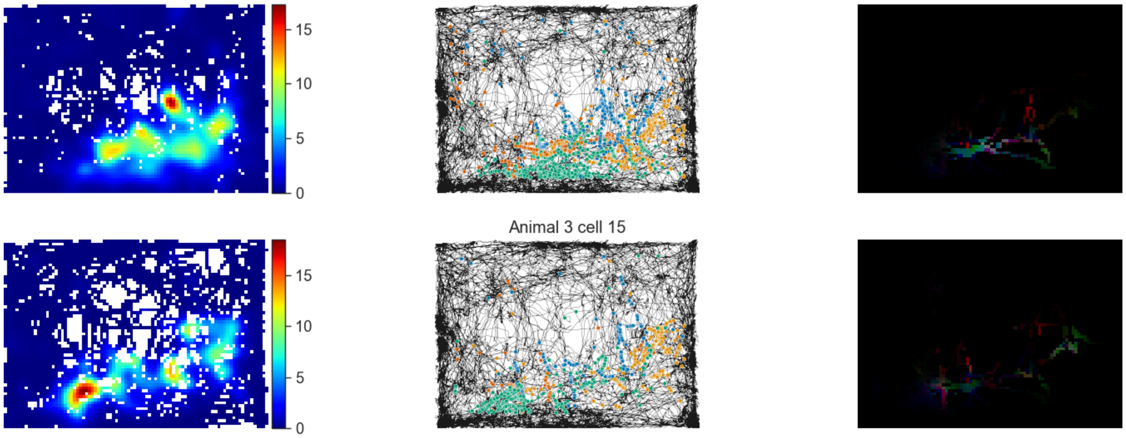
15
10
5
0
Animal 3 cell 15
15
10
5
0

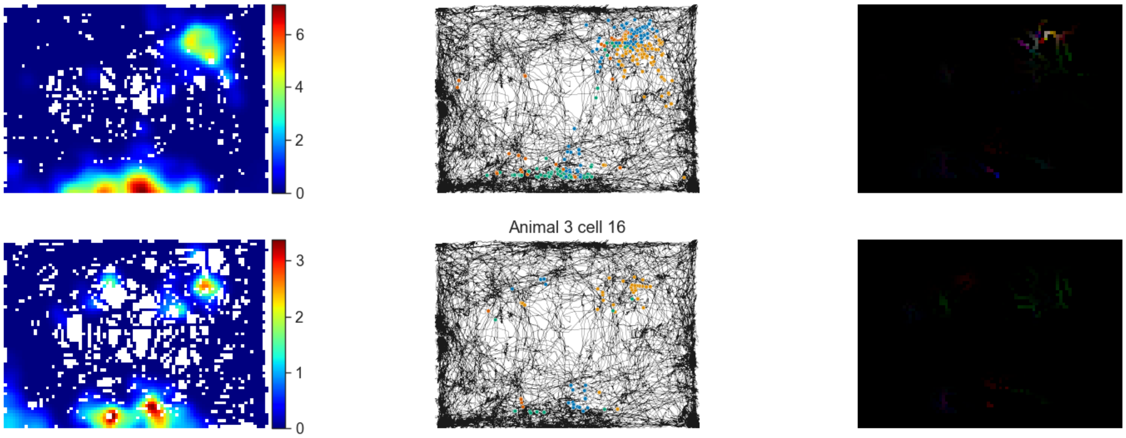
6
4
2
0
Animal 3 cell 16
3
2
1
0

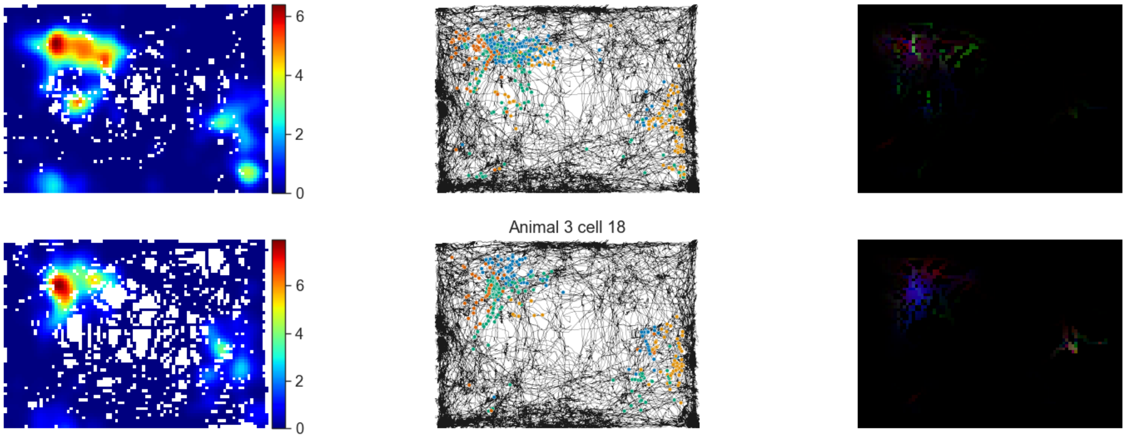
6
4
2
0
Animal 3 cell 18
6
4
2
0

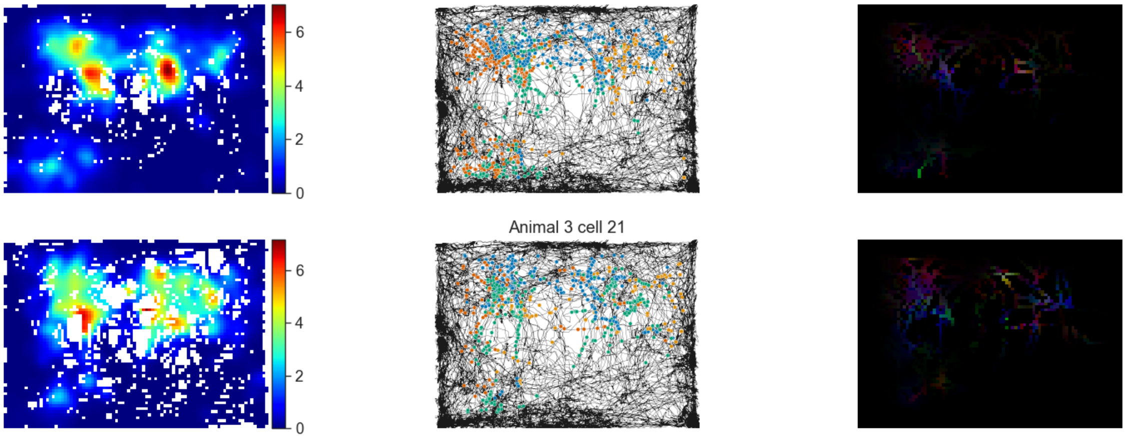
Animal 3 cell 21
0
2
4
6

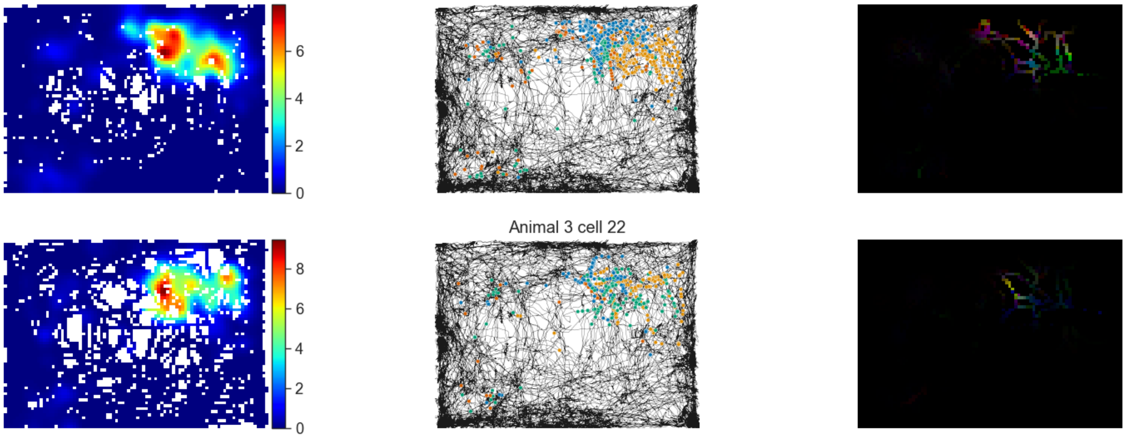
Animal 3 cell 22
0
2
4
6
8

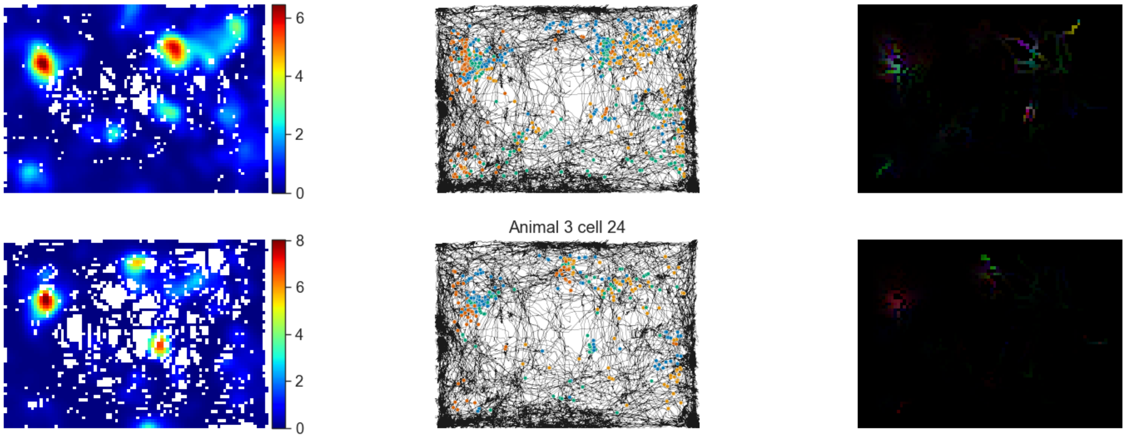
Animal 3 cell 24
0
2
4
6
8

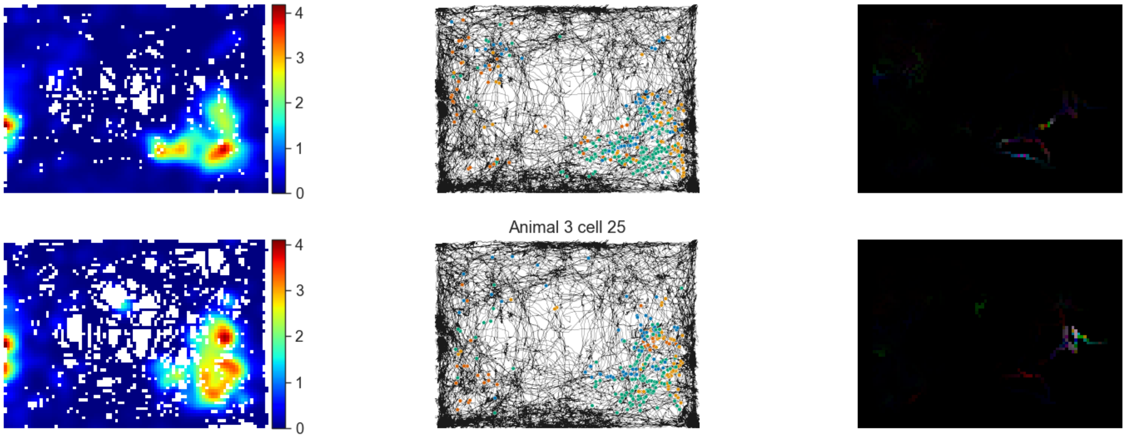
4
3
2
1
0
Animal 3 cell 25
4
3
2
1
0

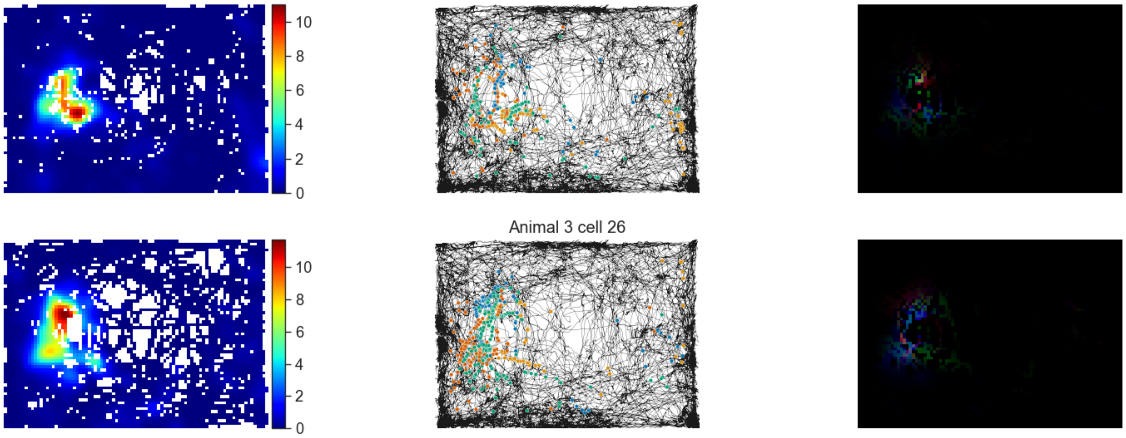
10
8
6
4
2
0
Animal 3 cell 26
10
8
6
4
2
0

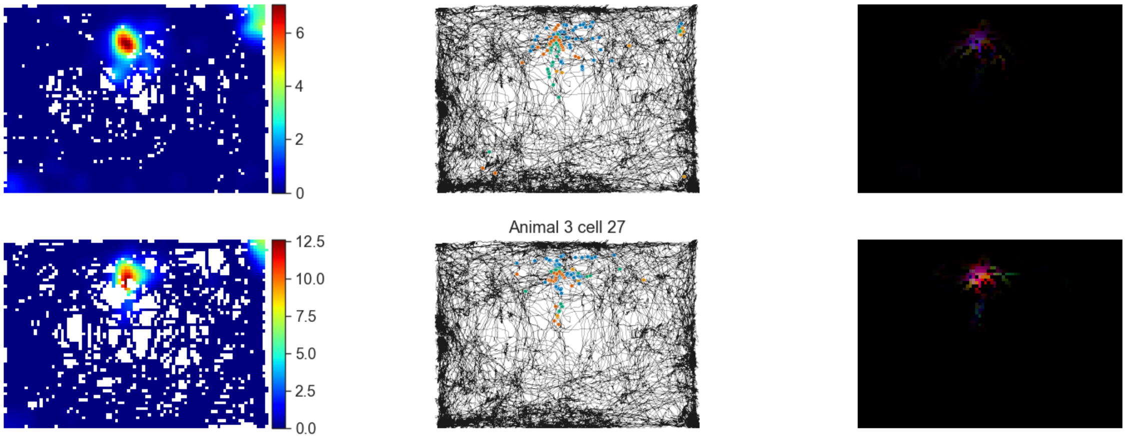
6
4
2
0
Animal 3 cell 27
12.5
10.0
7.5
5.0
2.5
0.0

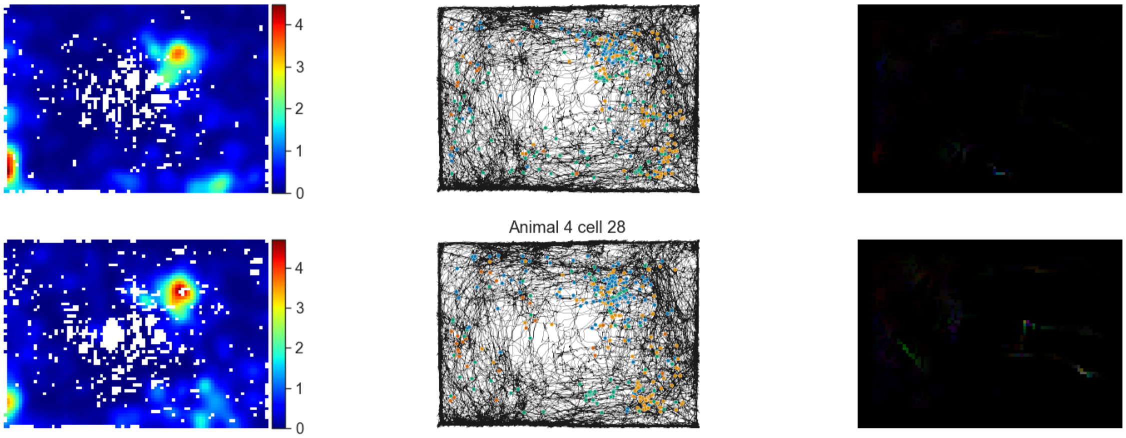
0
1
2
3
4
Animal 4 cell 28
0
1
2
3
4

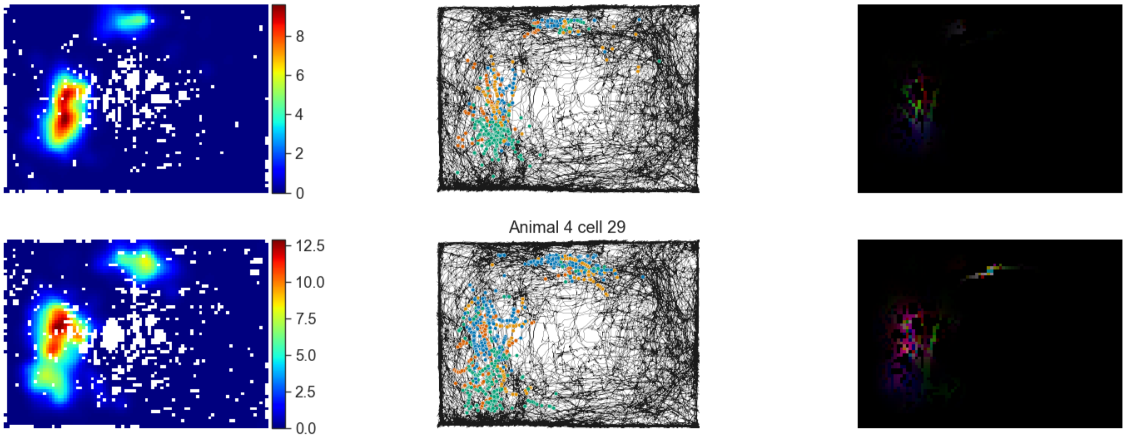
0
2
4
6
8
Animal 4 cell 29
0.0
2.5
5.0
7.5
10.0
12.5

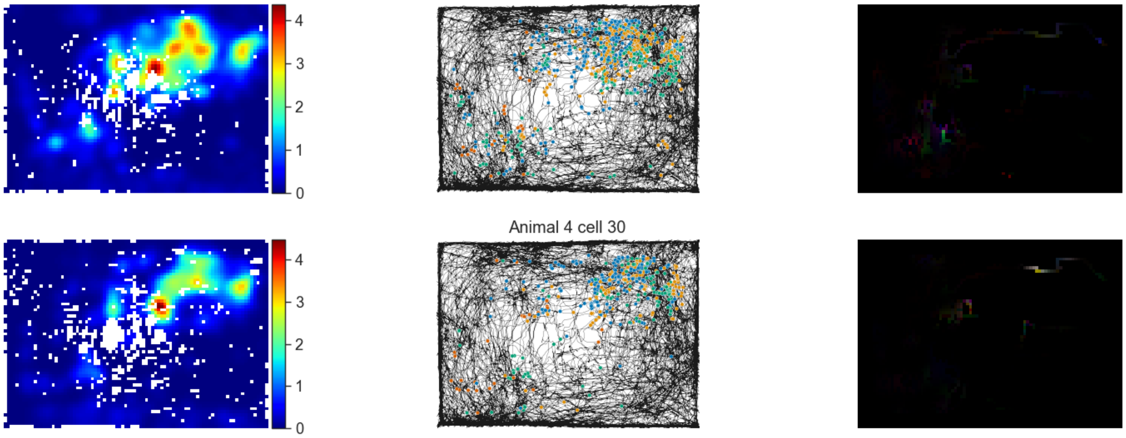
0
1
2
3
4
Animal 4 cell 30
0
1
2
3
4

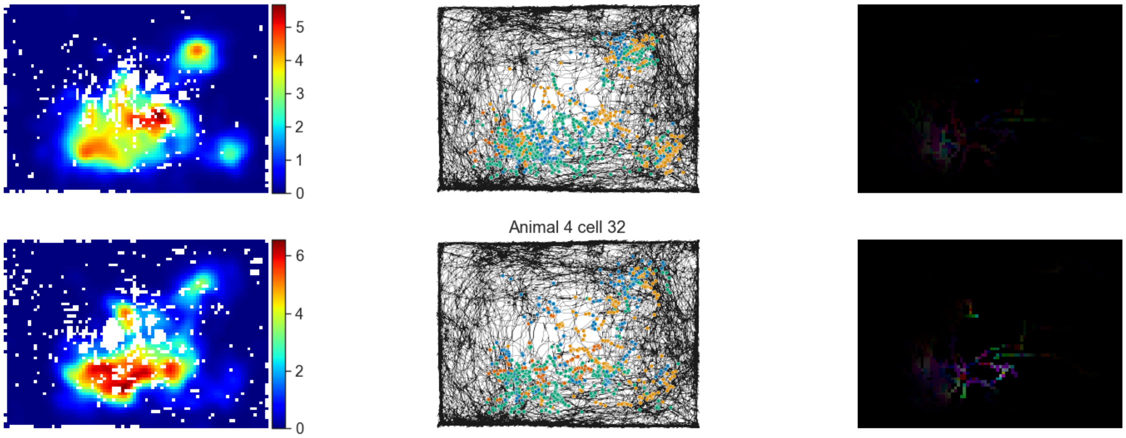
Animal 4 cell 32
0
1
2
3
4
5
0
2
4
6

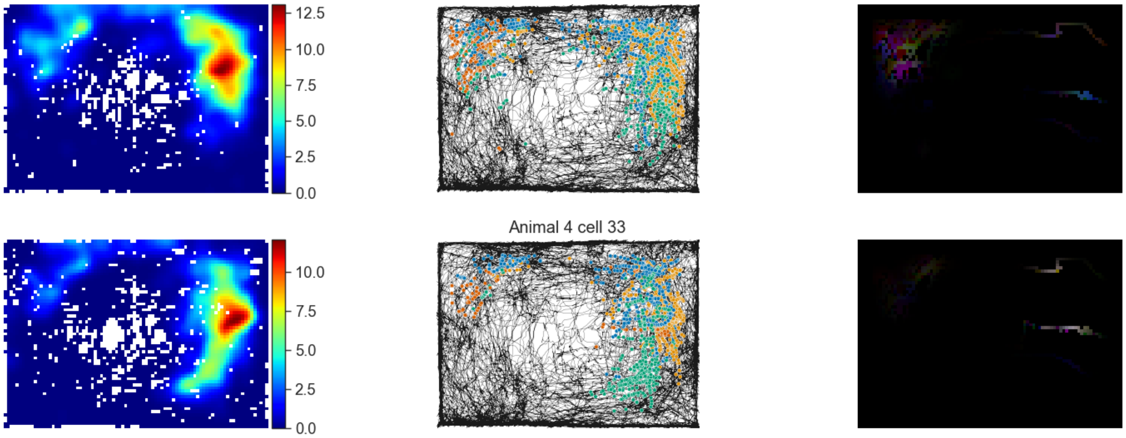
Animal 4 cell 33
0.0
2.5
5.0
7.5
10.0
12.5
0.0
2.5
5.0
7.5
10.0

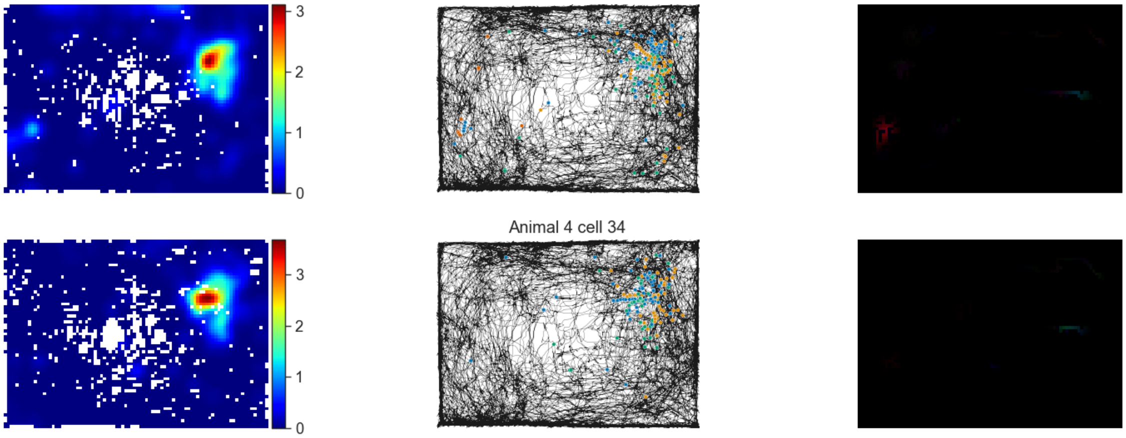
Animal 4 cell 34
0
1
2
3
0
1
2
3

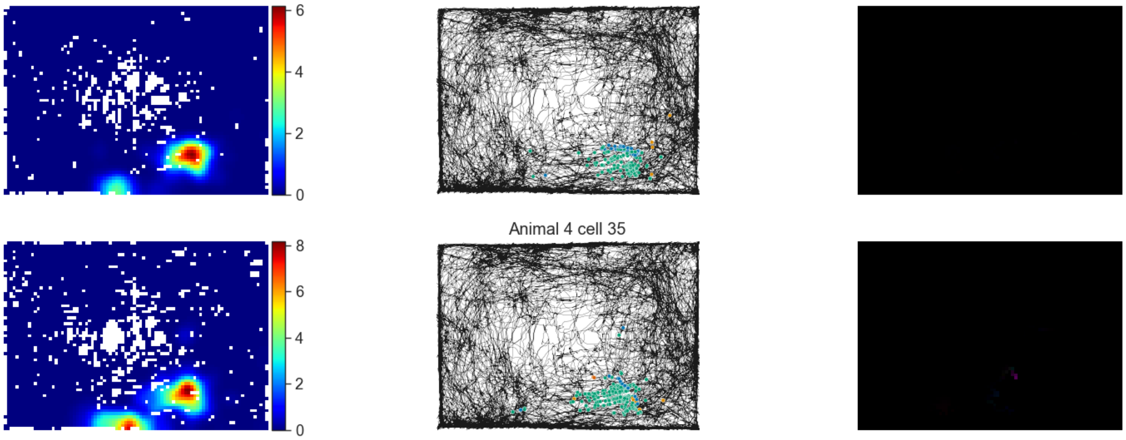
Animal 4 cell 35
0
2
4
6
8

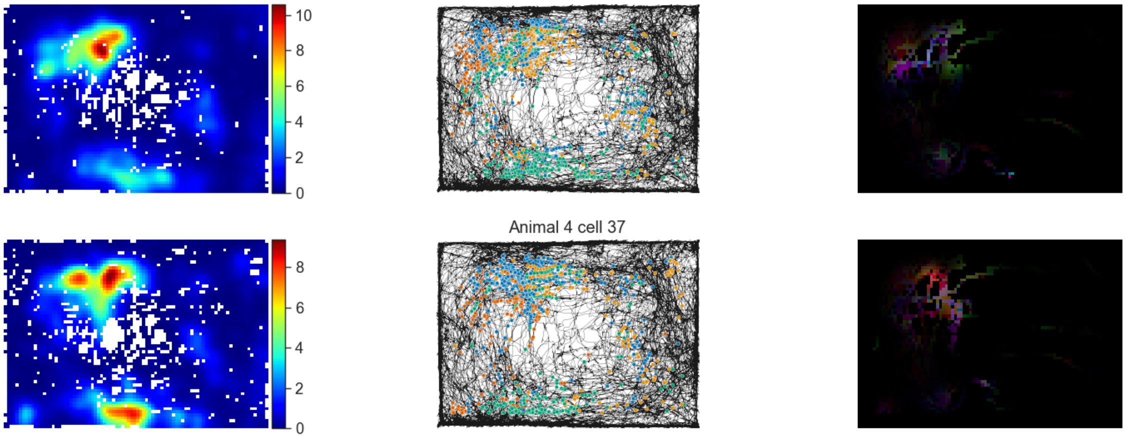
Animal 4 cell 37
0
2
4
6
8
10

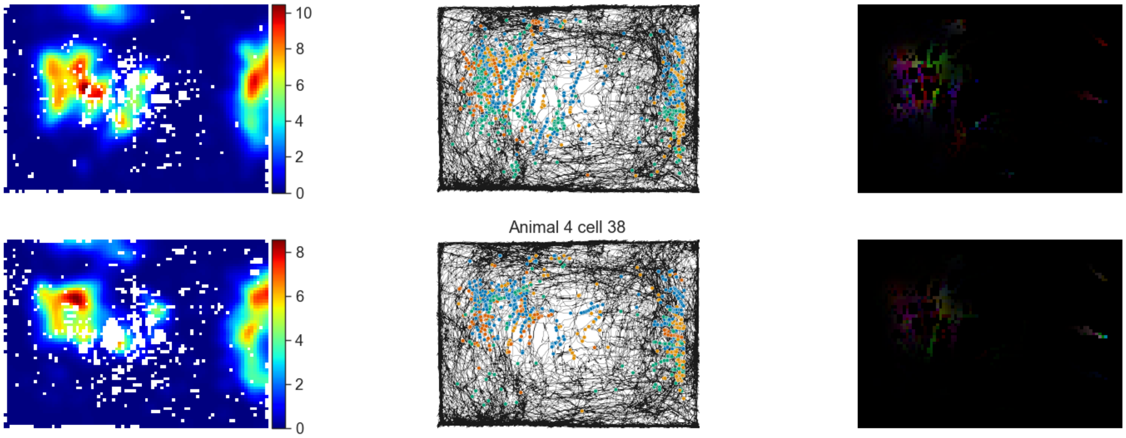
Animal 4 cell 38
0
2
4
6
8
10

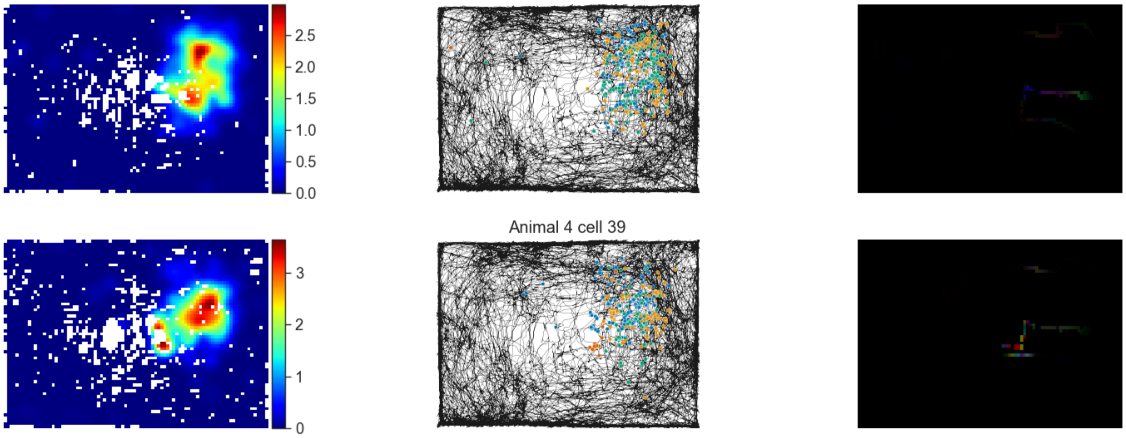
Animal 4 cell 39
2.5
2.0
1.5
1.0
0.5
0.0
3
2
1
0

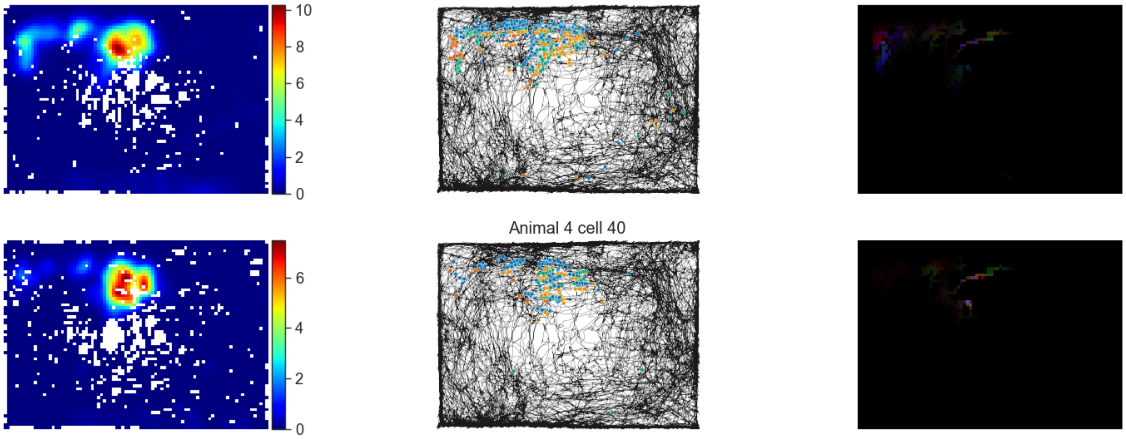
Animal 4 cell 40
10
8
6
4
2
0
6
4
2
0

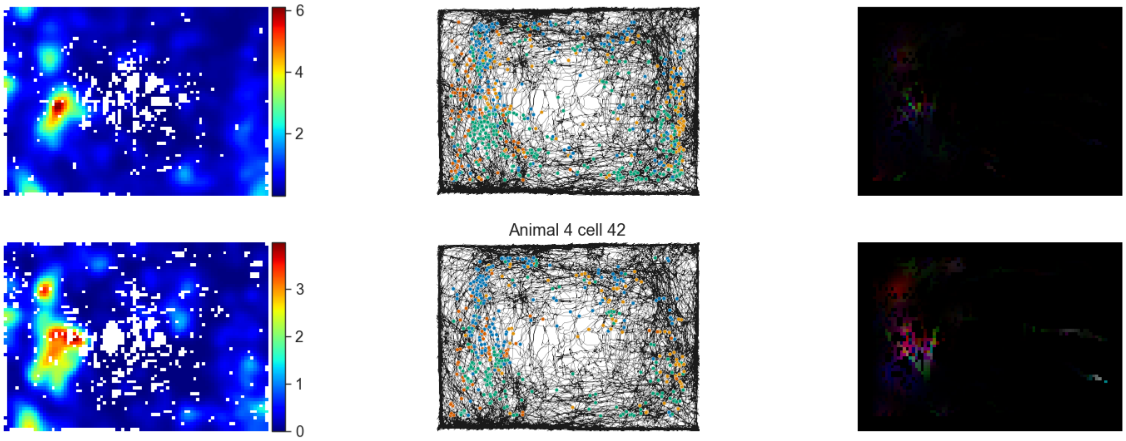
Animal 4 cell 42
6
4
2
3
2
1
0

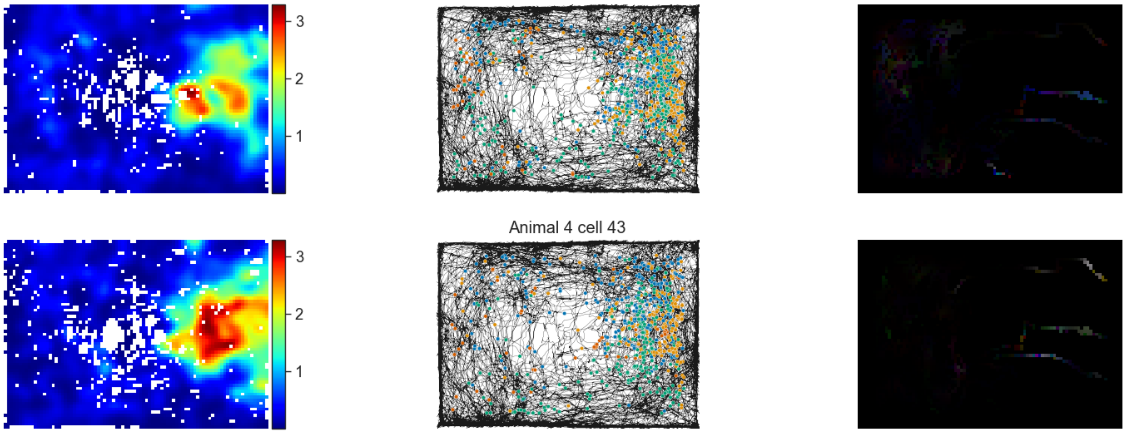
Animal 4 cell 43
3
2
1
3
2
1

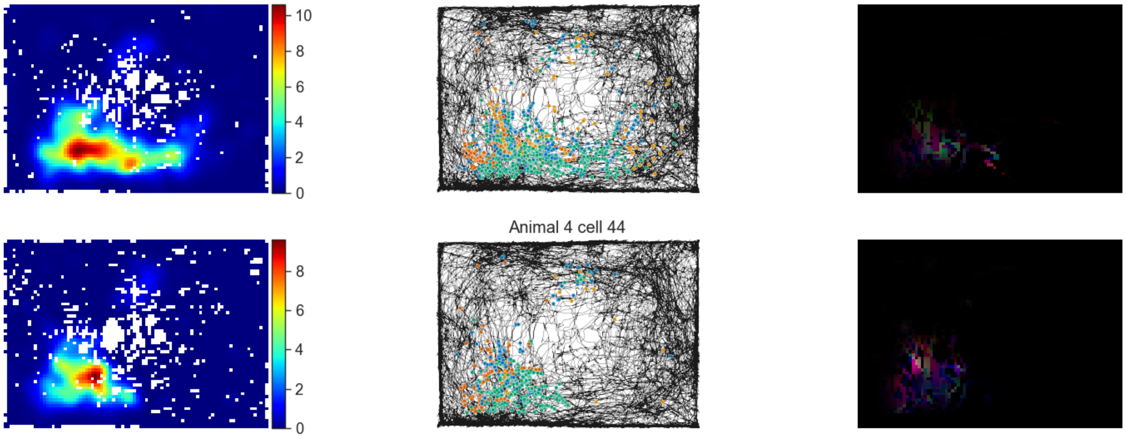
Animal 4 cell 44
10
8
6
4
2
0
8
6
4
2
0

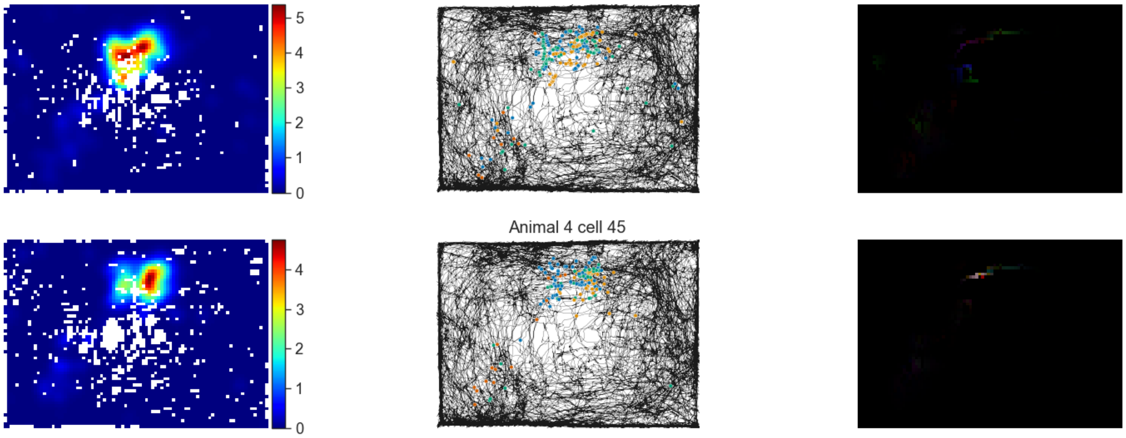
Animal 4 cell 45
5
4
3
2
1
0
4
3
2
1
0

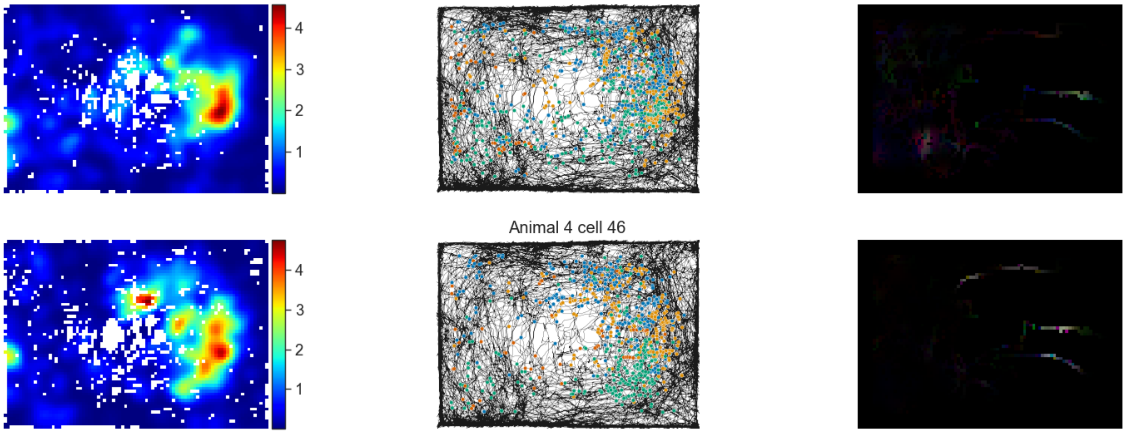
Animal 4 cell 46

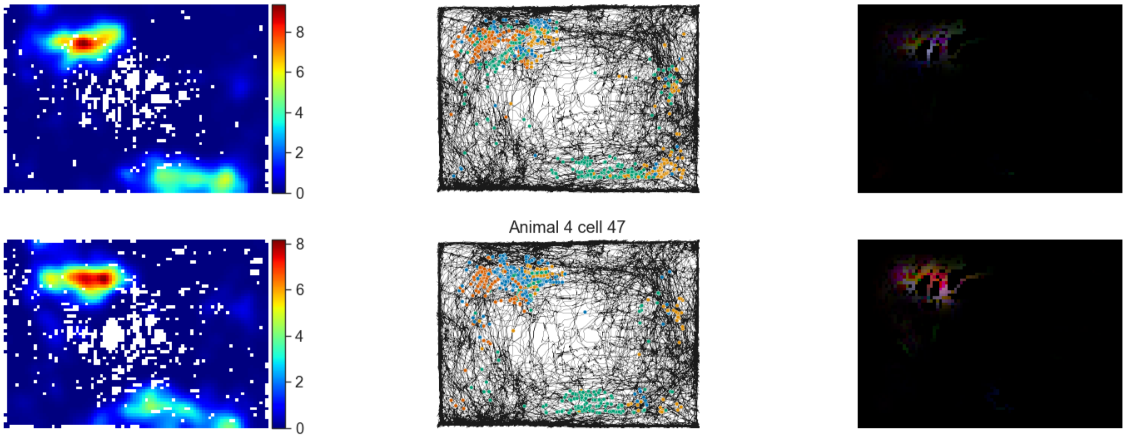
Animal 4 cell 47

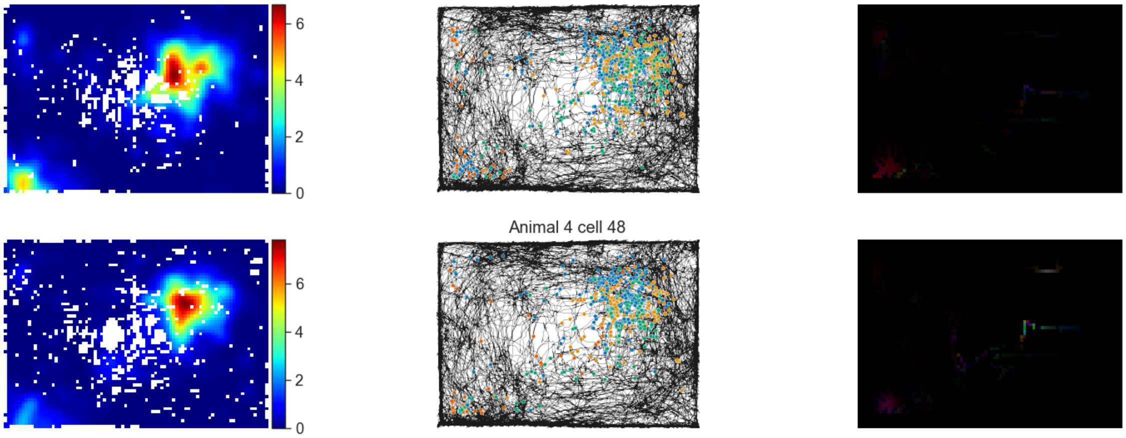
Animal 4 cell 48

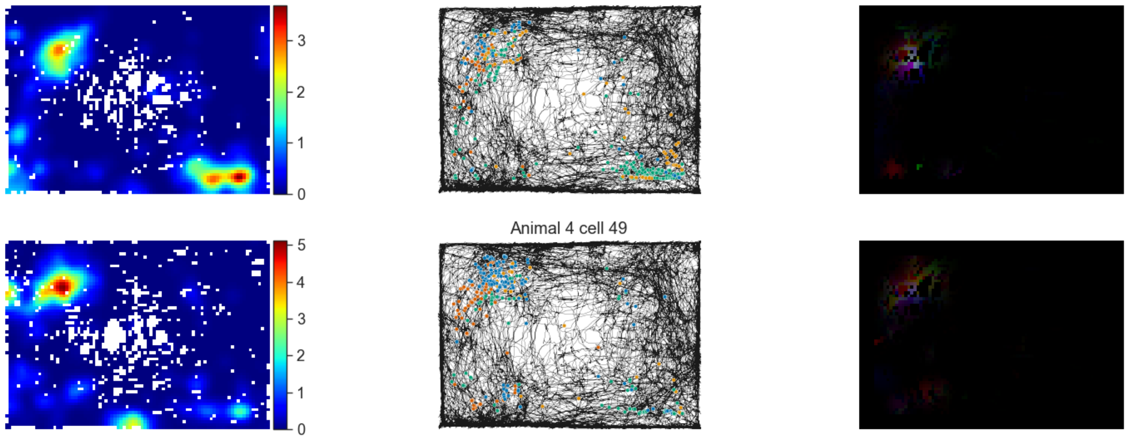
Animal 4 cell 49

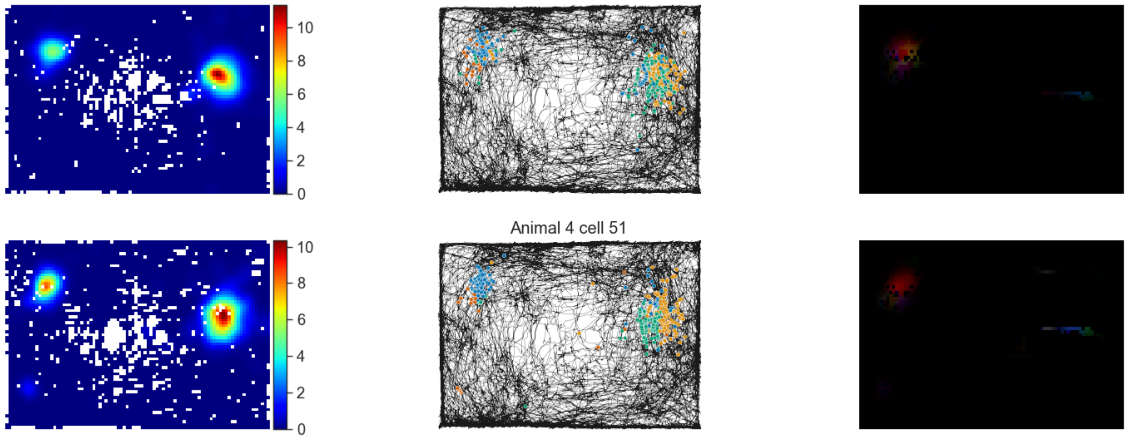
Animal 4 cell 51

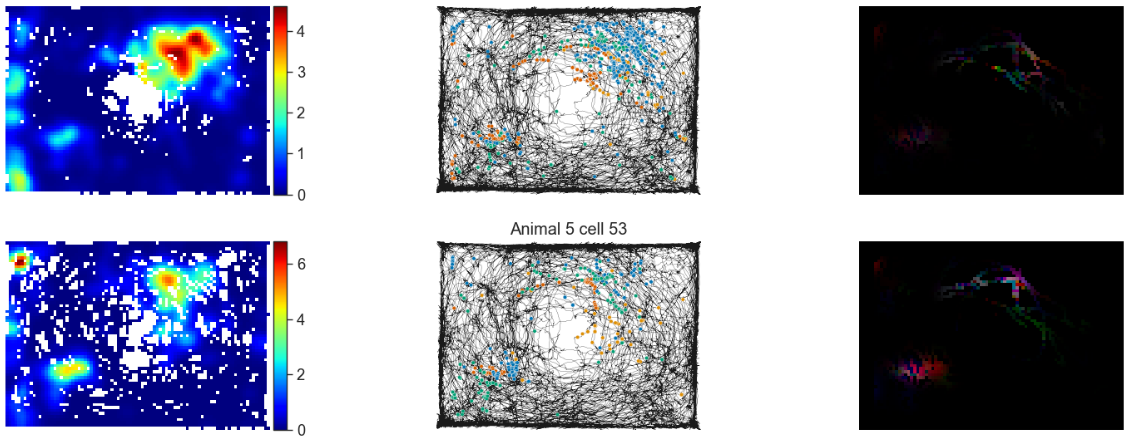
Animal 5 cell 53

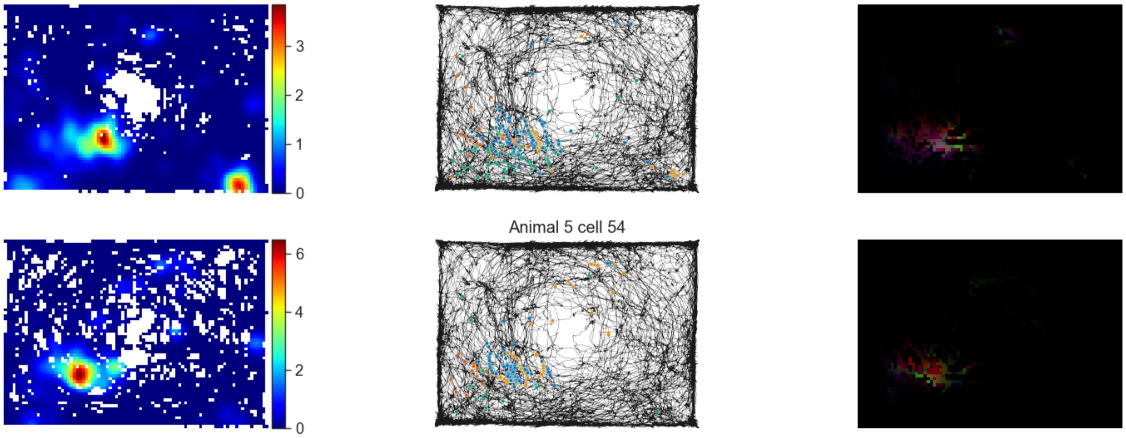
Animal 5 cell 54

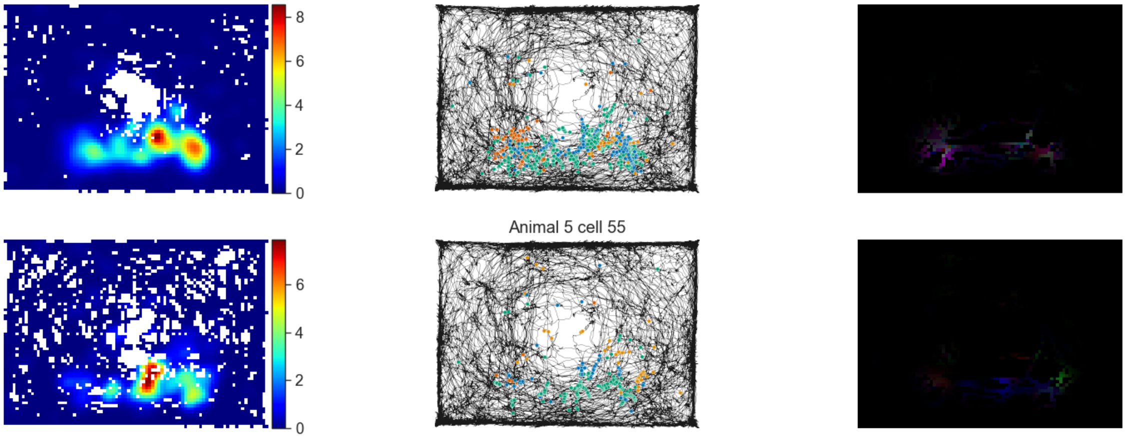
Animal 5 cell 55

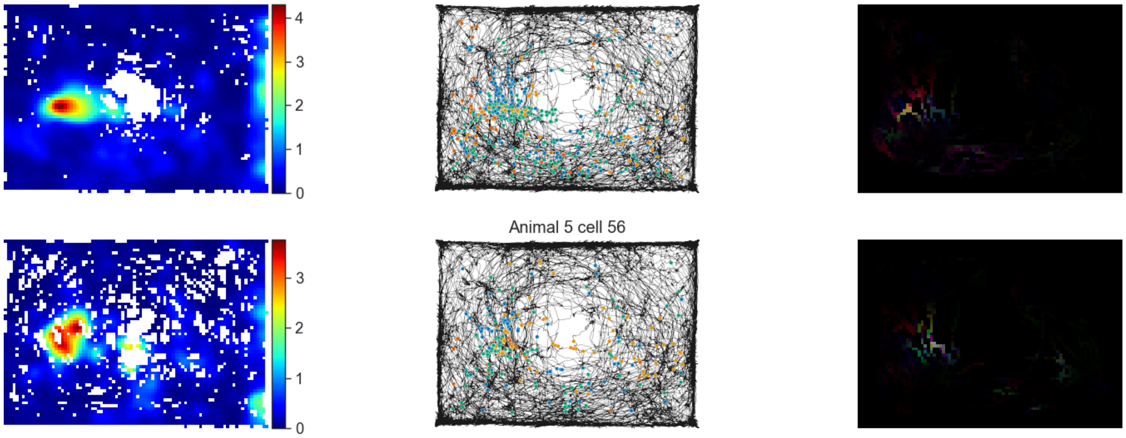
Animal 5 cell 56

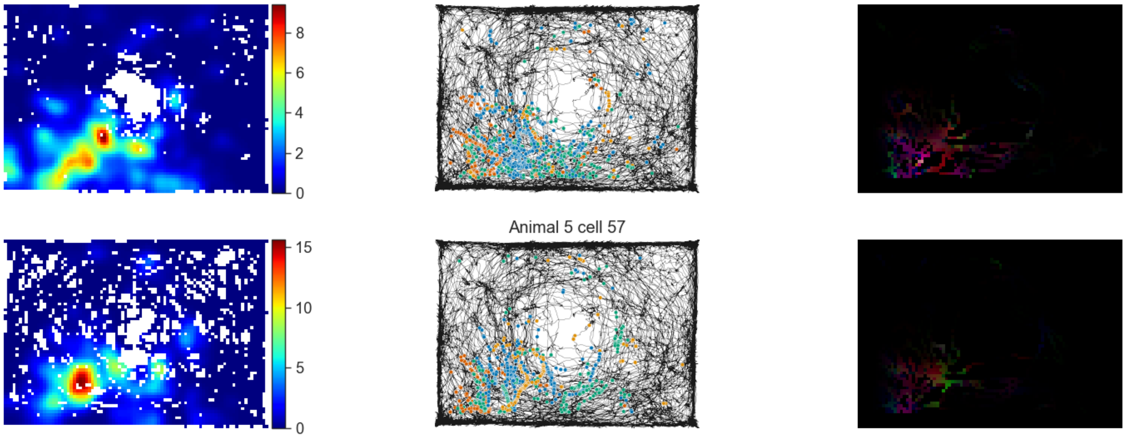
Animal 5 cell 57

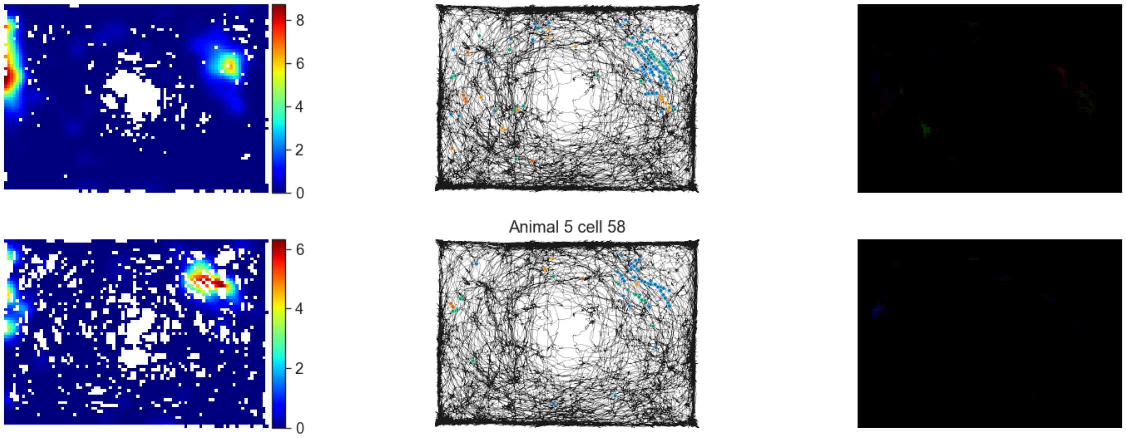
Animal 5 cell 58

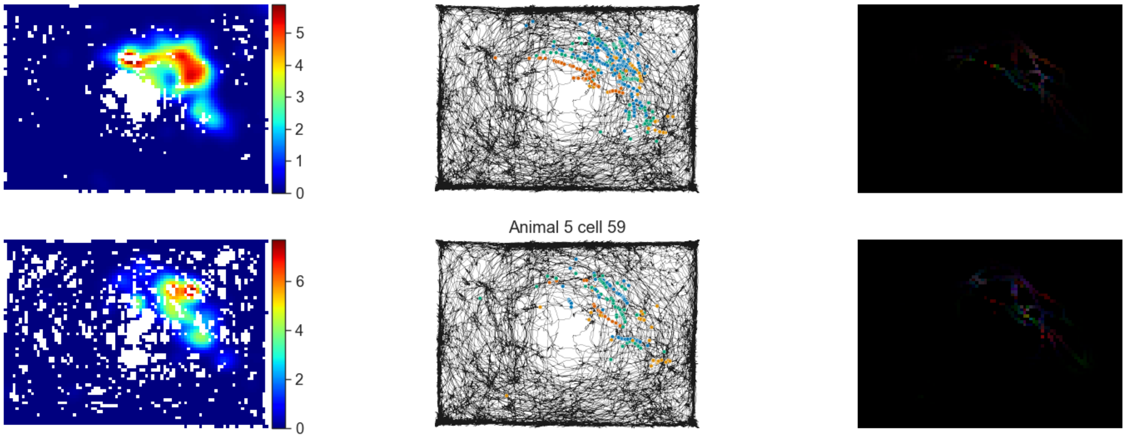
Animal 5 cell 59

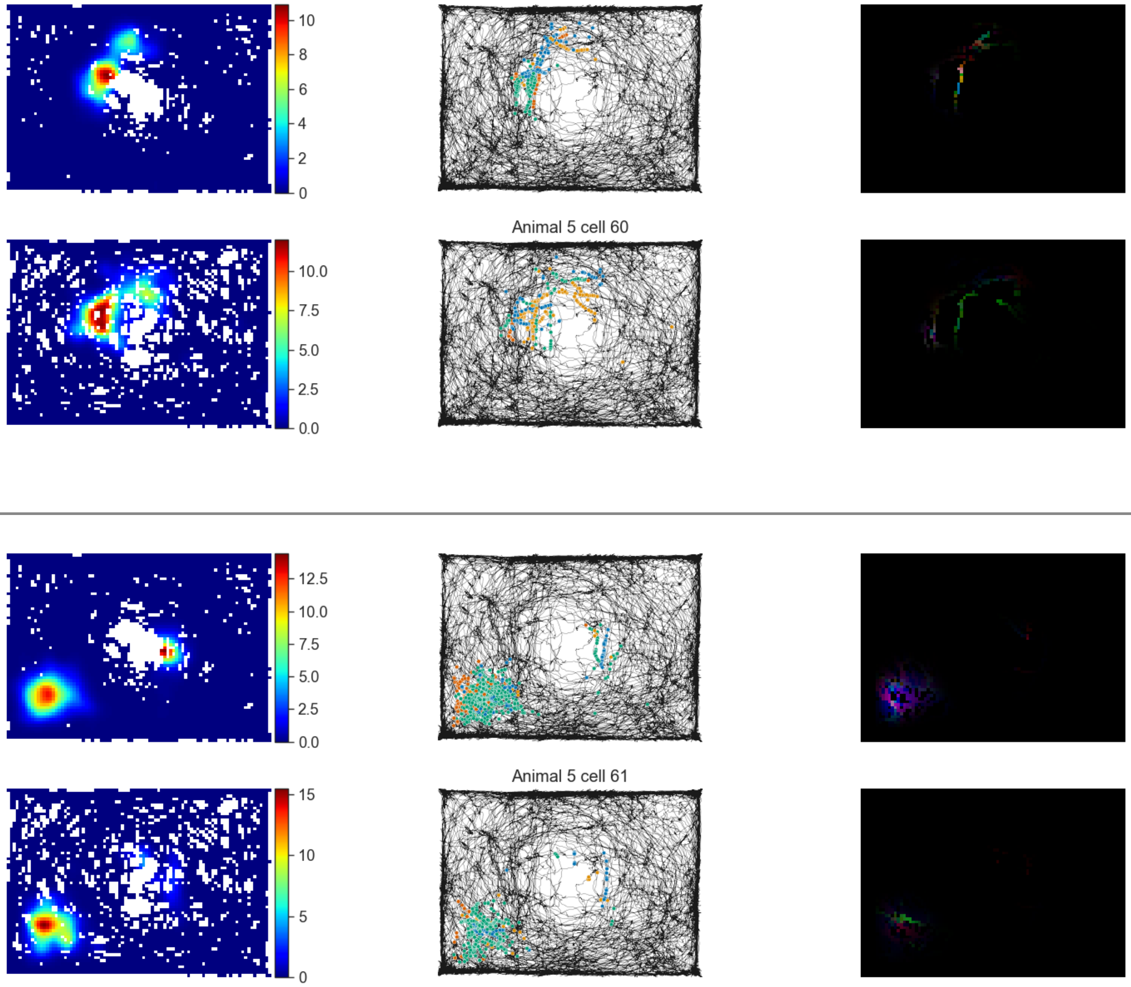

10
8
6
4
2
0
Animal 5 cell 60
10.0
7.5
5.0
2.5
0.0
12.5
10.0
7.5
5.0
2.5
0.0
Animal 5 cell 61
15
10
5
0

Data S1A

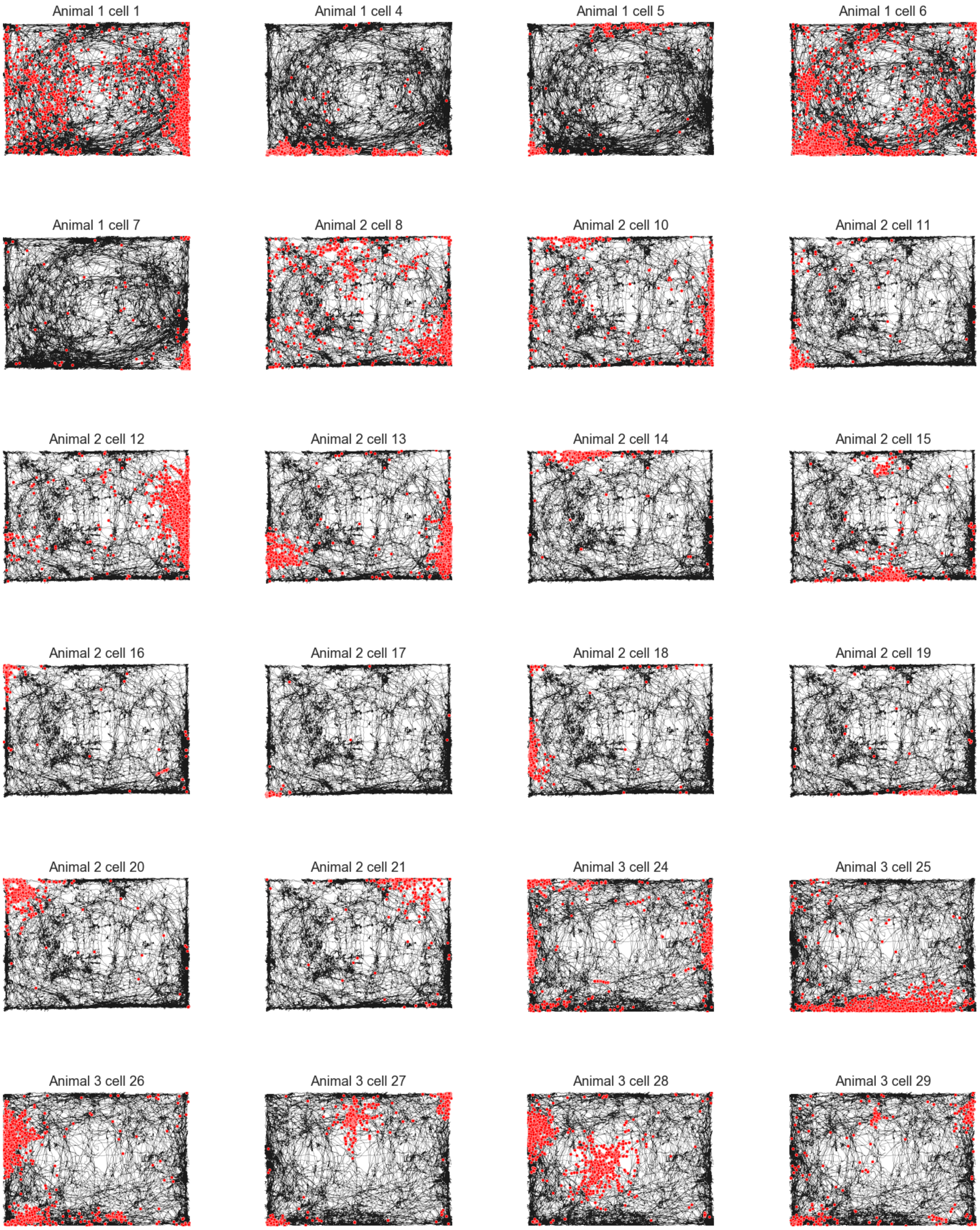
Animal 1 cell 1
Animal 1 cell 4
Animal 1 cell 5
Animal 1 cell 6
Animal 1 cell 7
Animal 2 cell 8
Animal 2 cell 10
Animal 2 cell 11
Animal 2 cell 12
Animal 2 cell 13
Animal 2 cell 14
Animal 2 cell 15
Animal 2 cell 16
Animal 2 cell 17
Animal 2 cell 18
Animal 2 cell 19
Animal 2 cell 20
Animal 2 cell 21
Animal 3 cell 24
Animal 3 cell 25
Animal 3 cell 26
Animal 3 cell 27
Animal 3 cell 28
Animal 3 cell 29

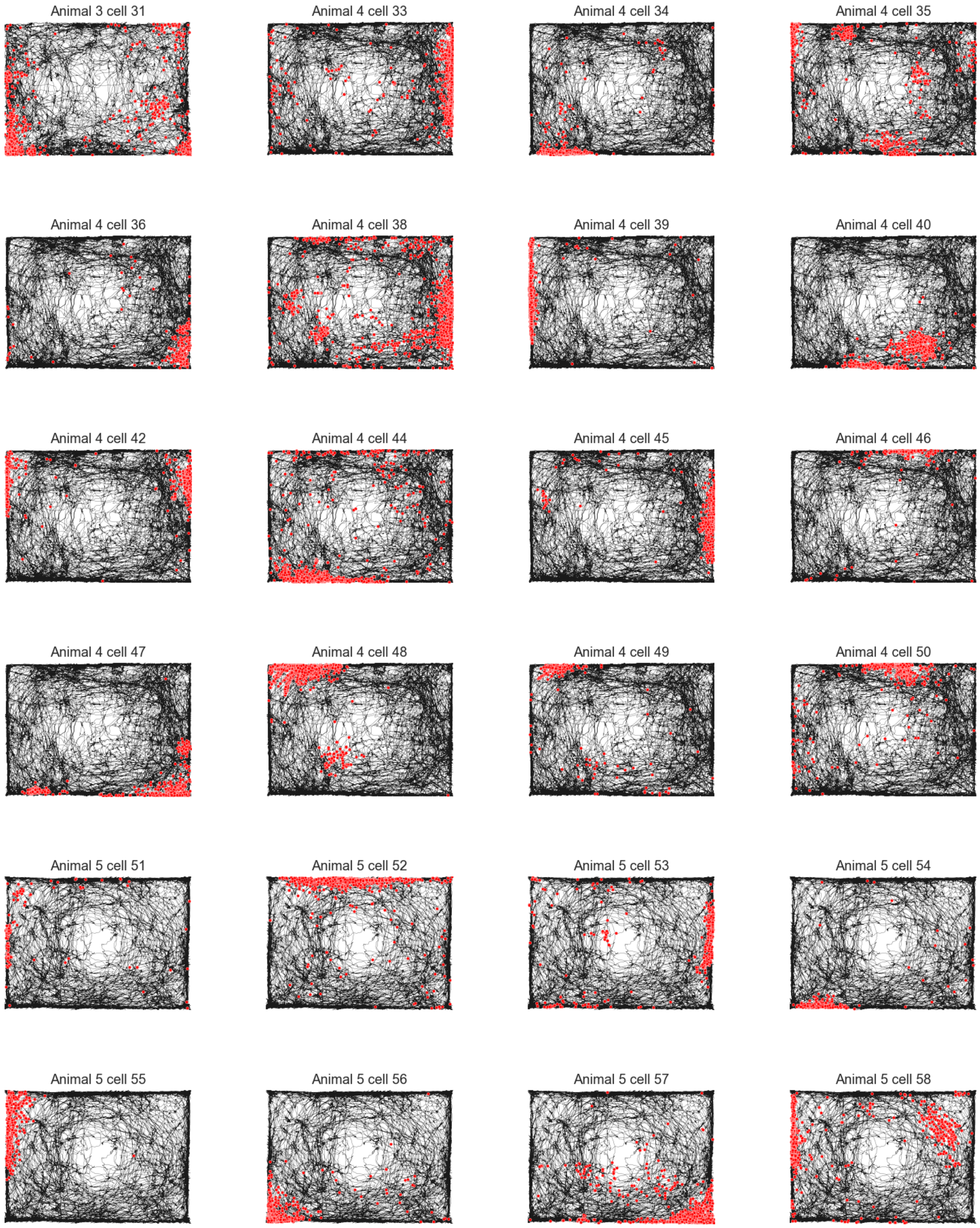
Animal 3 cell 31
Animal 4 cell 33
Animal 4 cell 34
Animal 4 cell 35
Animal 4 cell 36
Animal 4 cell 38
Animal 4 cell 39
Animal 4 cell 40
Animal 4 cell 42
Animal 4 cell 44
Animal 4 cell 45
Animal 4 cell 46
Animal 4 cell 47
Animal 4 cell 48
Animal 4 cell 49
Animal 4 cell 50
Animal 5 cell 51
Animal 5 cell 52
Animal 5 cell 53
Animal 5 cell 54
Animal 5 cell 55
Animal 5 cell 56
Animal 5 cell 57
Animal 5 cell 58

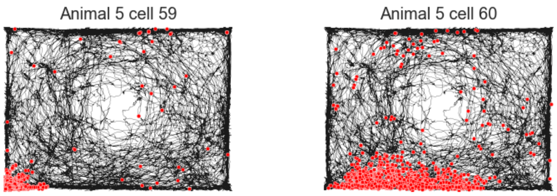

Data S1B
