# Supplementary material for: State transitions in the statistically stable place cell population correspond to rate of perceptual change
Source: Curr Biol. 2022 Aug 22;32(16):3505–3514.e7. doi: 10.1016/j.cub.2022.06.046 (PMC9616721; doi:10.1016/j.cub.2022.06.046)
Supplement: Document S2. Article plus supplemental information [file mmc3.pdf]

Current Biology

Article

State transitions in the statistically stable place cell population correspond to rate of perceptual change

Graphical abstract

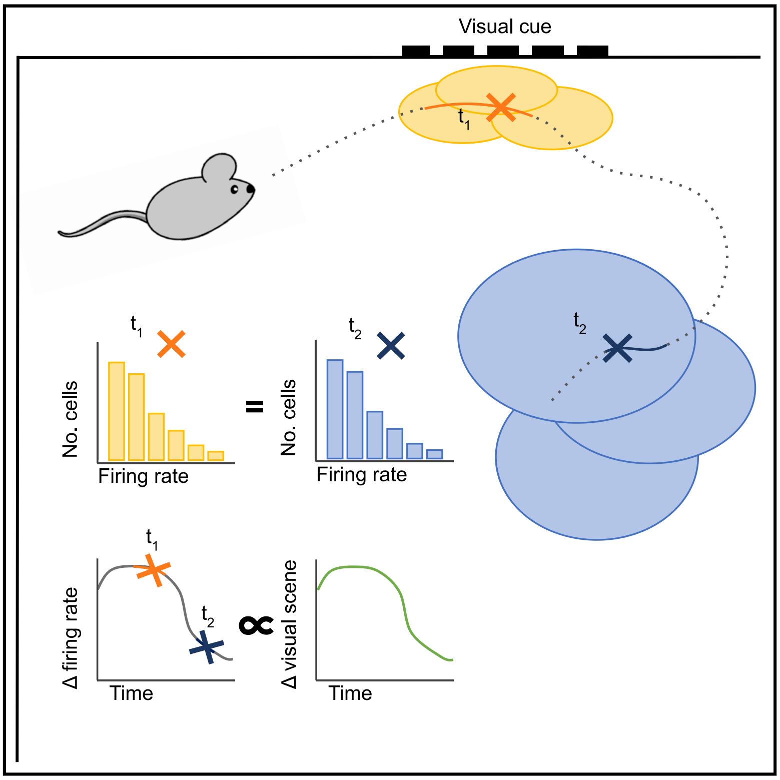

Highlights

- Neural activity in rodent CA1 place cell populations is homeostatically balanced
- Hippocampal place field size and frequency are governed by proximity to boundaries
- Transition rate through place cell population matches rate of change in visual scene

Authors

Sander Tanni, William de Cothi, Caswell Barry

Correspondence

caswell.barry@ucl.ac.uk

In brief

New work by Tanni, de Cothi, and Barry finds that place cell activity in the mammalian hippocampus is homeostatically controlled—the distribution of firing rates in the population is stable. During motion, the rate of transition through these cells matches the rate of change in the visual scene, yielding smaller, more numerous fields near walls.

Tanni et al., 2022, Current Biology *32*, 3505–3514
August 22, 2022 © 2022 The Author(s). Published by Elsevier Inc.
https://doi.org/10.1016/j.cub.2022.06.046

CellPress

Current Biology

CellPress
OPEN ACCESS

Article

State transitions in the statistically stable place cell population correspond to rate of perceptual change

Sander Tanni,[1] William de Cothi,[1] and Caswell Barry[1,2,3,*]
[1]Department of Cell and Developmental Biology, University College London, London, UK
[2]Twitter: @caswellcaswell
[3]Lead contact
*Correspondence: caswell.barry@ucl.ac.uk
https://doi.org/10.1016/j.cub.2022.06.046

SUMMARY

The hippocampus occupies a central role in mammalian navigation and memory. Yet an understanding of the rules that govern the statistics and granularity of the spatial code, as well as its interactions with perceptual stimuli, is lacking. We analyzed CA1 place cell activity recorded while rats foraged in different large-scale environments. We found that place cell activity was subject to an unexpected but precise homeostasis—the distribution of activity in the population as a whole being constant at all locations within and between environments. Using a virtual reconstruction of the largest environment, we showed that the rate of transition through this statistically stable population matches the rate of change in the animals' visual scene. Thus, place fields near boundaries were small but numerous, while in the environment's interior, they were larger but more dispersed. These results indicate that hippocampal spatial activity is governed by a small number of simple laws and, in particular, suggest the presence of an information-theoretic bound imposed by perception on the fidelity of the spatial memory system.

INTRODUCTION

Hippocampal place cells, pyramidal neurons in regions CA1 and CA3, are distinguished by their spatially constrained firing fields.[1] The activity of these cells, as a population, provides a sparse representation of self-location that is relatively independent of other variables—such as head direction and velocity[2,3]—and is believed to provide the neural basis of a cognitive map.[4] Fifty years of research have contributed extensively to our knowledge of this system, and today, place cells are understood to be common to mammals,[5,6] have been shown to be engaged as a temporal and abstract code,[7,8] and are known to be reactivated during periods of quiescence.[9,10]

Despite these achievements, our understanding of the dynamics that control the statistics and distribution of place cell representations has been slower to advance. In part, this is due to technical barriers that make it difficult to collect long-duration, high-yield recordings in animals as they explore large spaces. Despite these constraints, a small number of groups have conducted work in extended environments, showing that individual place cells can develop multiple fields[11] and exhibit distinct propensities to be recruited on long linear tracks.[12,13] Similarly, investigations along the hippocampal axis identified a gradient of spatial scales, with ventral cells having considerably larger place fields than dorsal cells.[14] Nevertheless, an understanding of the hippocampal population code on the implementational level, in Marr's terms,[15] is lacking. Effectively, we know little about the rules that govern activity in the place cell population, how it evolves across space, and how it is influenced by sensory information. Put simply, we do not know how place field size and density interact with each other and the environment. So, for example, although place fields are known to be smaller and more numerous in visually rich environments,[16] it is not clear whether these changes are linked and how they affect activity at the level of the entire population and, thus, the implication for information transfer to downstream structures.

One practical outcome of this situation is that we do not have sufficient empirical data to arbitrate between classes of computational models. For example, geometric cue-based models describe place field locations by integrating distance and direction from environmental features such as boundaries.[17,18] These models predict that the place fields near to boundaries are generally smaller, specifically being more compact perpendicular to the adjacent boundary, while more distant fields are expected to be diffuse. Notably, if fields simply became larger at locations more distant from boundaries, without some form of compensation, we would expect to observe a net increase in population firing rate toward the interior of environments. To offset this, the boundary-tuned precursors to place cells—boundary vector cells—are typically assumed to be more densely distributed adjacent to walls than at longer distances.[17,19] In contrast, models based on attractor dynamics, presumed to be instantiated in region CA3, tend to ignore any systematic variance in place field size and density across environments, emphasizing even coverage and carefully balanced activity.[20,21] These two classes of model, as well as others,[22–24] provide competing but not incompatible explanations of

This is an open access article under the CC BY license (http://creativecommons.org/licenses/by/4.0/).

hippocampal dynamics, yet the evidence needed to generate a synthesis is lacking.

Here we analyze large populations of place cells recorded while rats foraged in different-sized, equally proportioned environments of up to 8.75 m^2. We find that place fields and cells are recruited in proportion to environmental area but with a strong influence of location on field frequency and size—fields are smaller and more numerous near boundaries, whereas being larger and less numerous toward the environment's center. Surprisingly, these two effects counter each other exactly, resulting in stable population-level firing within and between environments. Thus, the proportion of co-active cells, mean firing rate, and distribution of activity across the population were preserved at all locations, suggesting the presence of a strong homeostatic mechanism governing place cell firing. Using a virtual reality (VR) replica of the recording environment, we show that the rate of change in the activity of the place cell population is strongly correlated with the rate of change in the animals' visual scenes. Thus, although the statistics describing the distribution of place cell activity were stable across time and space, the rate at which the components of this distribution varied corresponded with the amount of perceivable change experienced by the animal. Taken together, these results suggest that the size and extent of individual place fields are well described by geometric cue-based models, whereas the population as a whole conforms to the expectations of attractor-based models. More generally, as predicted by theory,[25,26] it appears that the effective scale of representations within the spatial memory system are limited by the perceptible information afforded by the environment.

RESULTS

Using extracellular electrodes (128 channels per animal), we recorded 629 CA1 place cells (89–172 cells/rat) from five rats while they foraged for randomly dispensed rewards (20 mg pellets) in four familiar, differently sized environments. The environments—designated A–D—were identically proportioned, with each being double the area of the previous one to a maximum of 8.75 m^2 (Figures 1A and S1A). The order of environments B, C, and D was randomized for each animal, with the smallest environment, A, being used at the beginning and end of the recording session. Recording duration was scaled proportional to environment area—15 to 120 min—and a single recording session consisting of 5 trials was analyzed from each animal (Figure 1B). Place cells were isolated based on their waveforms (Figure 1C) and temporal firing rate statistics (Figure 1D), with many cells being active in multiple environments (Figure 1E). The spatial rate maps of these cells were stable in all environments, with high intra-trial (1st versus 2nd half) spatial correlation across place cells (mean correlation by environment, range 0.58–0.65; Figure S1C; Data S1A). Individual place fields were detected iteratively as contiguous regions of stable firing rates continuously increasing toward a peak (STAR Methods) (Figure S1B). There was no correlation between the number of place fields detected in the largest environment and the intra-trial (1st and 2nd half) spatial correlation ($r = -0.071$, $p = 0.100$, $n = 537$), indicating that the observed multiple fields were not caused by intra-trial remapping.

As is typical of the hippocampal system, the spatially modulated activity of neurons in environment A was stable both within and between recordings (Figure S2A; intra-trial [A ½ versus ½] spatial correlation, 0.61; inter-trial [A versus A′], 0.51; inter-trial with shuffle [A versus shuffled A′], −0.03), while activity in the four different environments was highly separable, being sufficient to assign 94.5% of activity vectors (1 s duration) to the correct environment (STAR Methods) (Figure S2B).

In all animals, the number of active place cells—neurons with at least one spatial field—was greater in larger environments (Figures 2A and 2D), as was the total number of place fields (Figure 2B). The number of fields per cell was not correlated with the cells' clustering quality, and only one animal exhibited a correlation between the L-ratio and field count (Table S1), indicating that the observed place cells with multiple fields were not a result of multiple single-field cells being assigned to the same cluster. Place field recruitment increased more rapidly than the number of active cells, such that the average number of fields per cell was higher in larger environments (1.28 fields/cell in A to 2.28 fields/cell in D; Figure 2C). The same place cells were recorded in all environments, allowing for the consideration of the total spatial representation of the place cell population. When all recording environments were considered collectively (total area 16.4 m^2), an approach used previously in the context of hippocampal spatial memory capacity,[13] it was more common for cells to have 2 fields than 1, and 84.8% of all cells had multiple fields either within (Data S1B) or across environments (Figure 2C). Furthermore, consistent with previous studies,[12,13] we found that individual cells had different propensities to form place fields, and that this proclivity was maintained across environments (Figure S3C), meaning that cells with numerous fields in one environment were more likely to have numerous fields in another. Thus, the number of fields per cells were better fit with a gamma-Poisson model—which allows for cells to have different rates of field formation—than an equal-Poisson model[13]—which assumes the same frequency of field formation for all place cells (log-likelihoods, −1,538 versus −1,679; Bayesian information criterion, 3,089 versus 3,365). If all cells formed fields at the same rate, then the number of fields per cell would follow a Poisson distribution, with its mean determined by the environment area and field formation rate.[12,13] In our data, this simpler equal-Poisson model consistently overestimates the proportion of cells with 4–6 fields across the collective environment (Figures S2D–S2F). In contrast, the gamma-Poisson model accurately fits the rate at which place cells were recruited ($\geq$1 field) as a function of environment area (Figure 2D), predicting that 99% of CA1 place cells will have at least one place field in environments greater than 51.8 m^2 (Figure 2D, inset).

To quantify the relationship between the number of place fields and environment size, we examined how field recruitment varied with area—normalizing place field counts by total number of fields detected in each animal. We found a remarkably robust linear regression with a positive intercept ($r = 0.983$, $p = 10^{-14}$, slope = 0.038, intercept = 0.093, $n = 20$; Figure 2E). The r value for the fitted line can be viewed as being particularly high because each datapoint—the proportion of place fields detected per animal, per environment—is the average of a large number of samples (each animal has several hundred place fields) and

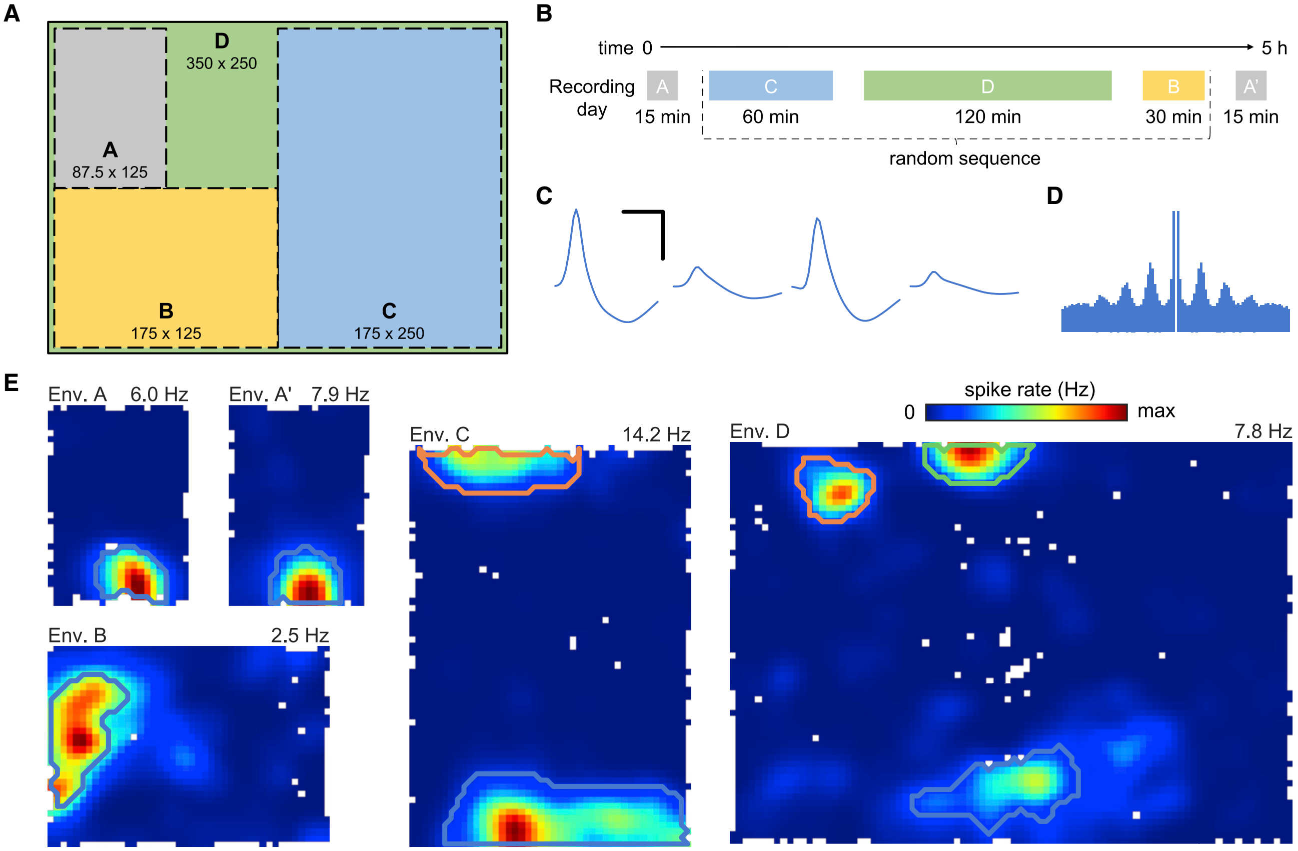

Figure 1. Place cell recordings in multiple large environments

(A) Schematic of the four environments (A, B, C, and D), illustrating their relative sizes (cm) and positions in the experimental room. Each environment was distinguished by a set of unique cues (Figure S1A).

(B) Rats foraged in all environments during the recording session— environment A twice, at the start and end of the session, interleaved with the other three in random order. Recording duration scaled linearly with environment area.

(C–E) Waveforms (scale bars, 0.5 ms and 100 μV), auto-correlogram (maximum lag 500 ms), and rate maps for a typical place cell with activity in all environments (bin size 4 cm). Distinct place fields are delineated with lines of different color (see Figure S1B for details on field detection). The color map for each plot scales from 0 Hz to the peak rate above each map. Unvisited bins are white.

See also Data S1.

therefore is less susceptible to fluctuations. Notably, this linear fit indicates that the proportion of place fields per unit area is lower in larger spaces (Figure 2E, inset; Kruskal-Wallis: H = 17.9, p = 4.71 × 10^{-4}, n = 5).

Next, to understand why place fields were less numerous per unit area in large environments, we examined how fields were distributed within environments. To this end, we segmented the space into concentric bands according to distance to the nearest boundary (each band 25 cm wide). In environments B–D, for which at least two bands could be defined, we calculated the density of place field peaks and found that it was greater near to the walls, reducing toward the environment center (Kruskal-Wallis tests: env. B, H = 4.81, p = 0.028; env. C, H = 12.5, p = 0.002; env. D, H = 16.1, p = 0.001; n = 5; Figure 3A). Equally, considering only the band closest to the wall (<25 cm), field peak density was generally higher in smaller environments (Figure 3A; Kruskal-Wallis: H = 12.4, p = 6.02 × 10^{-3}, n = 5). These effects could not be explained by the difference in dwell time between locations (Figure S3A). In direct contrast, the average size of place fields increased with distance from the wall (Kruskal-Wallis tests: env. B, H = 6.82, p = 0.009; env. C, H = 10.2, p = 0.006; env. D, H = 10.5, p = 0.015; n = 5; Figure 3B) and were smaller in environment A (Kruskal-Wallis: H = 15.9, p = 0.001, n = 5; Figure 3B). In particular, it appeared that the predominant factor contributing to this effect was that the field width in a given axis was proportional to the nearest wall distance along that axis (Figure S3B) and not to wall distance orthogonal to that axis (Figures 3C and 3D; Kruskal-Wallis: H = 12.1, p = 0.007, n = 5; H = 0.39, p = 0.94, n = 5, respectively). The average field size of a cell also did not correlate with its clustering quality (Table S1). Therefore, compared with locations near the walls of an enclosure, there were on average fewer individual place fields further from the walls, but those fields that were present tended to be larger.

Considered alone, the observed decrease in the density of field peaks away from the boundary would be expected to result in fewer active place cells, yielding a lower mean firing rate. Conversely, the increase in field size would lead to a

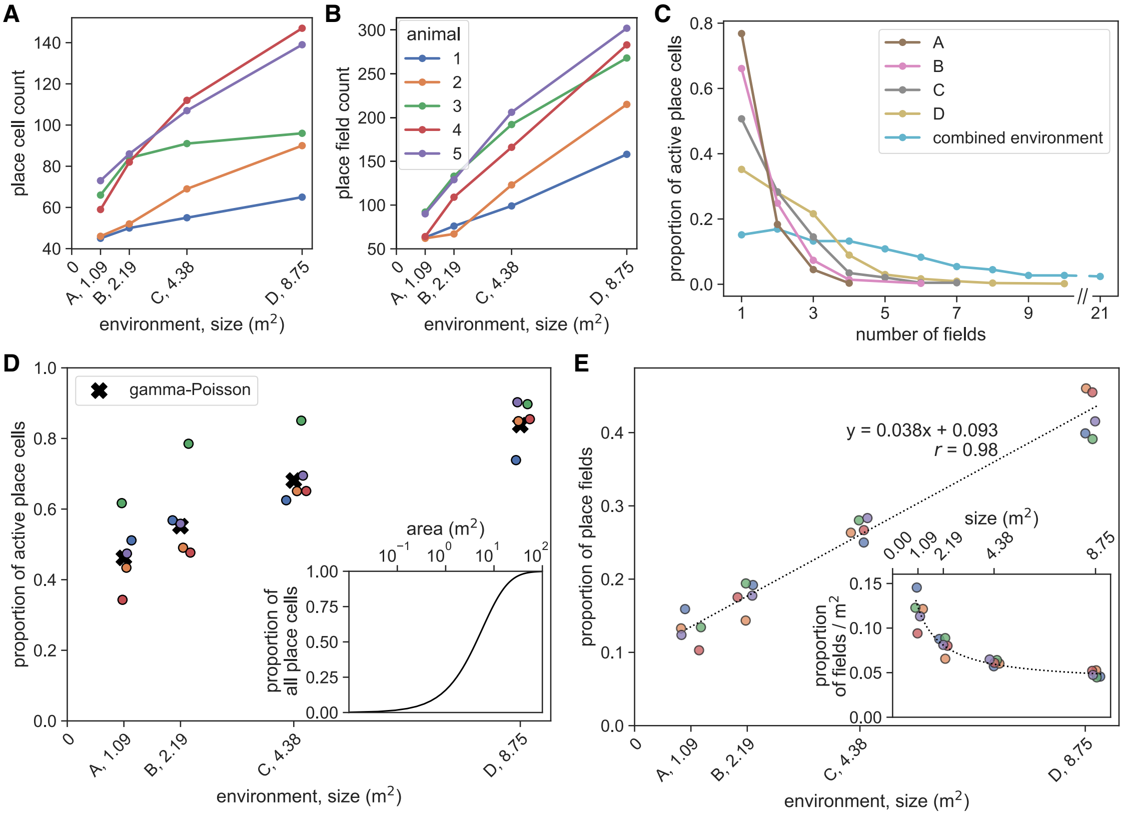

Figure 2. Place cells are more likely to be active and have more fields in large environments

(A and B) Number of active place cells (A) and place fields (for normalized values, see D) (B) by environment area (for normalized values, see E).

(C) Distribution of place field counts per cell by environment. Only cells with at least one field in any environment are included. Variance in field count was not determined by clustering quality (Table S1).

(D) The proportion of active place cells (at least one field in one or more environments) in each animal that had a field in a given environment increased with area (Kruskal-Wallis: H = 14.1, p = 0.003, n = 5) and was closely matched by a gamma-Poisson model fit to field numbers in a combined environment (Figure S2D), adjusted by the relative field densities from inset in (E) (STAR Methods) (mean squared error 0.0099). Inset shows the same gamma-Poisson model extrapolated to predict CA1 place cell recruitment in very large environments.

(E) Larger environments had more place fields, here shown as a proportion of all fields detected in each animal across the four environments (Kruskal-Wallis: H = 17.6, p = 5.36 × 10^{-4}, n = 5), scaling linearly with environment area (dashed line, linear regression fit, r = 0.982, p = 10^{-14}, n = 20). Inset shows place field count by environment area, regression line shown with same parameters as main plot (Kruskal-Wallis: H = 17.9, p = 4.71 × 10^{-4}, n = 5). Animal colors same as (B); points jittered to facilitate visualization.

greater overlap between place fields, resulting in the opposite outcome. Remarkably, we found that these two effects were exactly balanced, meaning that neither the proportion of co-active place cells (firing rate > 1 Hz) nor the mean firing rate of the population varied with distance from the wall (Figures 4A and 4B; Kruskal-Wallis: H = 1.2, p = 0.77 n = 5; H = 0.61, p = 0.89, n = 5, respectively). This was the case within and across all environments (Figures S4A and S4B). Similarly, interneuron firing rates were also constant at different distances to the bounding walls (Figure S4C; Kruskal-Wallis: H = 0.23, p = 0.97, n = 5). Furthermore, the distribution of firing rates in the place cell population was also stable across different distances to the wall, as there was no difference in how variable the firing rate distributions were in different recording halves at the same location or at different locations (see STAR Methods for more details; comparison of distribution divergence within and between locations with Mann-Whitney: U = 45, p = 0.43, n = 4 and 18; Figure 4C). These results are underlined by a very high correlation between the environment size and the total place field area in that environment, expressed as a proportion of the total area of all fields recorded from an animal (linear regression: r = 0.996, p = 10^{-20}, slope = 0.06, intercept = 0.006, n = 20) (Figure 4D). The r value for this correlation can be viewed as being particularly high because each datapoint is aggregating across a large number of place fields (between 50 and 300) and so is less susceptible to fluctuations.

Thus, taken together, there appear to be several consistent and related features of the place cell code for space. First, the total area of place fields active in an environment is a near perfect linear function of the environment's area. Second, population activity is homeostatically balanced, maintaining a constant proportion of active cells (15%) and mean firing rate (0.52 Hz/place cell)—despite field size growing with distance to walls. Finally, and more generally, the distribution of activity in the place cell population is also stable across space.

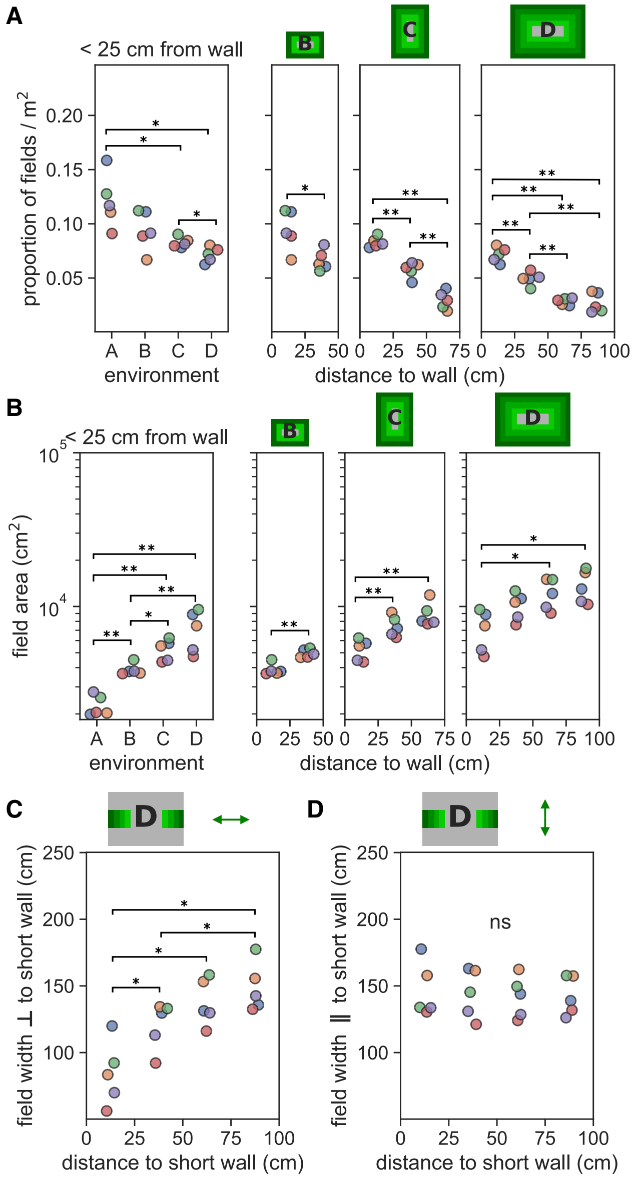

Figure 3. Density of place field peaks and the size of fields change with distance from the environmental boundary

(A) Place field peak density per unit area (field peak/m^2) is lower near the wall in large environments compared with small environments (left) and decreased with distance from the wall (right). Cartoon above each plot indicates wall distances in shades of green. Pairwise post hoc tests for this and subsequent panels adjusted for multiple comparisons using Benjamini-Hochberg (non-negative) correction. *p = 0.05; **p = 0.01; ns, not significant.

(B) The average area of place fields increased with distance from the wall and were larger in bigger environments when the wall distance was controlled for. Variance in field area was not determined by clustering quality (Table S1).

(C) The average width of place fields measured orthogonal to the nearest wall (Figure S3B) in environment D increased with distance from the wall. The cartoon above the plot indicates the wall distances and includes locations in the environment in green bands, and the arrow indicates the axis of measurement.

(D) The average width of place fields measured parallel to the short wall was the same in all distance bins. The same observation was made in recordings from all environments (Figures S3C and S3D).

The distribution of firing rates in the place cell population is constant, but the size of place fields and the density of their peaks varies systematically; this implies that hippocampal activity must change at different rates depending on the location of the animal and the direction in which it is moving (Figures S5E–S5J). Specifically, because place field width in a given axis is strongly determined by distance to the nearest wall orthogonal to that axis, the activity vector (see STAR Methods on population activity change) should change fastest when the animal is near a wall and moving directly toward or away from it. Thus, analyzing only trajectories running orthogonal to the short wall of the largest environment (D), we found that the Euclidean distance between activity vectors (1-cm intervals) was the greatest when animals were close to the wall (Kruskal-Wallis: H = 12.2, p = 0.007, n = 5; Figure 5A), an effect that was not found for parallel runs (Kruskal-Wallis: H = 3.78, p = 0.29, n = 5; difference between orthogonal and parallel, Mann-Whitney: U = 2, p = 0.018, n = 5; Figure 5B). Similar effects were confirmed in the other environments (Figures S5A and S5B). There was no correlation between this measure and theta frequency, while accounting for running speed in partial correlation analysis (Figure S5C), despite prior work having shown that theta oscillations organize place cell firing and field sizes.[27] Note that by analyzing the instantaneous rate of change in the population as a whole, we avoid the difficulty of estimating field size adjacent to boundaries, where it is difficult to determine how much of a field would exist outside the walls of the environment. This same analysis also eliminates the need to derive measures from individual rate maps, which represent average spatial firing over time and can be distorted when neural activity is modulated by factors such as heading direction.[28] By replicating the analysis of Keinath et al.,[28] we did not observe a systematic offset in the cross-correlograms between opposing boundary-tethered rate maps (t(99) = 1.1, p = 0.27), which may be of concern given the imperfect overlap of fields between runs (Data S1A); however, using instantaneous rate of change further maximizes the fidelity of our analysis against such errors.

Finally, to investigate whether visual information contributed to this effect, we developed a VR replica of environment D (Figure S5D). A 300° field of view was used to reconstruct each animals' visual scene at 1-cm increments along its trajectory (Figure 5C), and the visual change between consecutive frames was calculated during motion (speed >10 cm/s; STAR Methods). We found a tight coupling between the change in visual scene and change in place cell population activity (Figures 5D and 5G; time series correlation collapsed across all animals: Pearson's r = 0.60, p < 0.001, n = 510,871), which is a stronger relationship than that was found for other behavioral variables (Figure 5G) and which persisted when they were accounted for (partial correlation collapsed across all animals: r = 0.51, p < 0.001, n = 510,871). As expected, when mapped out in space, both the activity vector change and visual change showed a general increase with proximity to the walls, corners, and the two wall-mounted cues (Figures 5E and 5F; no smoothing applied), as well as a strong correlation between both maps (correlation between maps collapsed across all animals: Pearson's r = 0.72, p < 0.001, n = 5,524). Importantly, filtering the behavior by heading direction (Figure 5H) emphasizes how this variation depends not only on position but also on movement

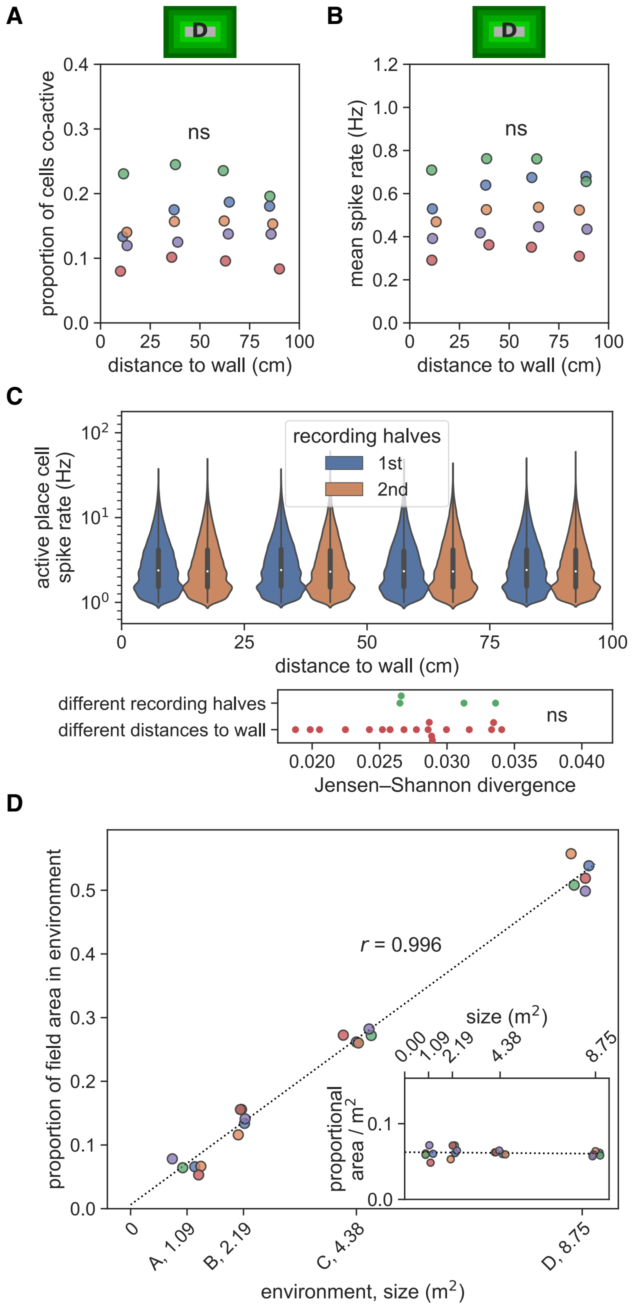

Figure 4. Distribution of activity within the place cell population remains constant despite changes in place field properties

(A and B) The proportion of active place cells (>1 Hz) (A) was constant at different distances to the wall in environment D, as was the mean firing rate of all place cells (B), as well as all other environments (Figure S4). ns, not significant.

(C) The distributions of firing rates, combining spikes across animals, at different distances to the wall in environment D, and split temporally between the first and second recording halves (top). Jensen-Shannon divergence measured between temporal recording halves and different distances to wall (bottom).

(D) The proportion of total place field area accounted for by place fields in each environment, computed separately for each animal, is highly correlated with the size of the environment (dashed line). Inset shows the values of the main plot divided by the size of each environment—the proportion of total place field area per square meter of each environment.

direction, with walls and cues orthogonal to the direction of motion being key factors in driving the visual change—a pattern that closely mirrored the direction-filtered activity vector change (direction-filtered map correlations collapsed across all animals: Pearson's $r > 0.66$, all $p < 0.001$, $N > 5{,}306$ for all 4 cardinal heading directions).

DISCUSSION

Using high-yield recordings from rodents foraging in large environments, we have shown that although the distribution and extent of individual place fields are governed by proximity to environmental boundaries, the statistics of the population activity as a whole are effectively constant. Specifically, the widths of place fields displayed a Weber's law-like[29] increase with distance to the orthogonal wall, which was exactly opposed by a commensurate decrease in field count. Thus, on average, small place fields were densely distributed near the boundaries of the environments, while broader fields were found more sparsely toward the middle. Practically, this means that the combined field area of all active place cells scales linearly with environment area because the reduced size of place fields in small environments is offset by their increased number and vice versa. Therefore, while the instantaneous activity in the population was stable, the rate of change through the population depends on both boundary proximity and movement direction, in a manner that correlates strongly with the rate of change in the animals' reconstructed visual scenes.

Place cells have often been conceptualized as pure allocentric representations of space fully abstracted from sensory stimuli,[4] and at first glance, this seems difficult to reconcile with the results reported here. However, our findings encapsulate a large body of prior work, suggesting that perceptual experience plays a larger than expected role in the formation of spatial representations. Thus, results showing that individual cells have more and larger fields in bigger spaces[11,13] can be understood in terms of a balance between homeostasis at the population level and the amount of perceptual change, which is naturally elevated near boundaries and cues, as well as other locations where the visual scene changes rapidly as the animal moves. Equally, this same relationship accounts for the clustering of place fields near visual cues in a 1D VR,[16] as well as a similar clustering of fields close to the walls,[30] doorways,[31] and other environmental features[19] of physical spaces. It is also consistent with the observation that place cell firing in a virtual environment predominantly reflects visual inputs following a manipulation of the relationship between motion inputs and visual gain[32] and can be sustained solely on the basis of visual cues when an animal is moved passively.[33,34] However, given that place cell responses can be oriented by sounds and smells[35,36] and persist in darkness,[37] as well as in congenitally blind rodents,[38] it seems highly likely that information from other modalities also contributes to the fidelity of spatial representations. Hence, more generally, we propose that the resolution of the hippocampal code for space is determined by the rate of change in the cross-modal perceptual environment. Recent observations that rodent place fields concentrate at the interfaces between different textures,[39] points where the tactile milieu changes rapidly, support this view.

CellPress
OPEN ACCESS

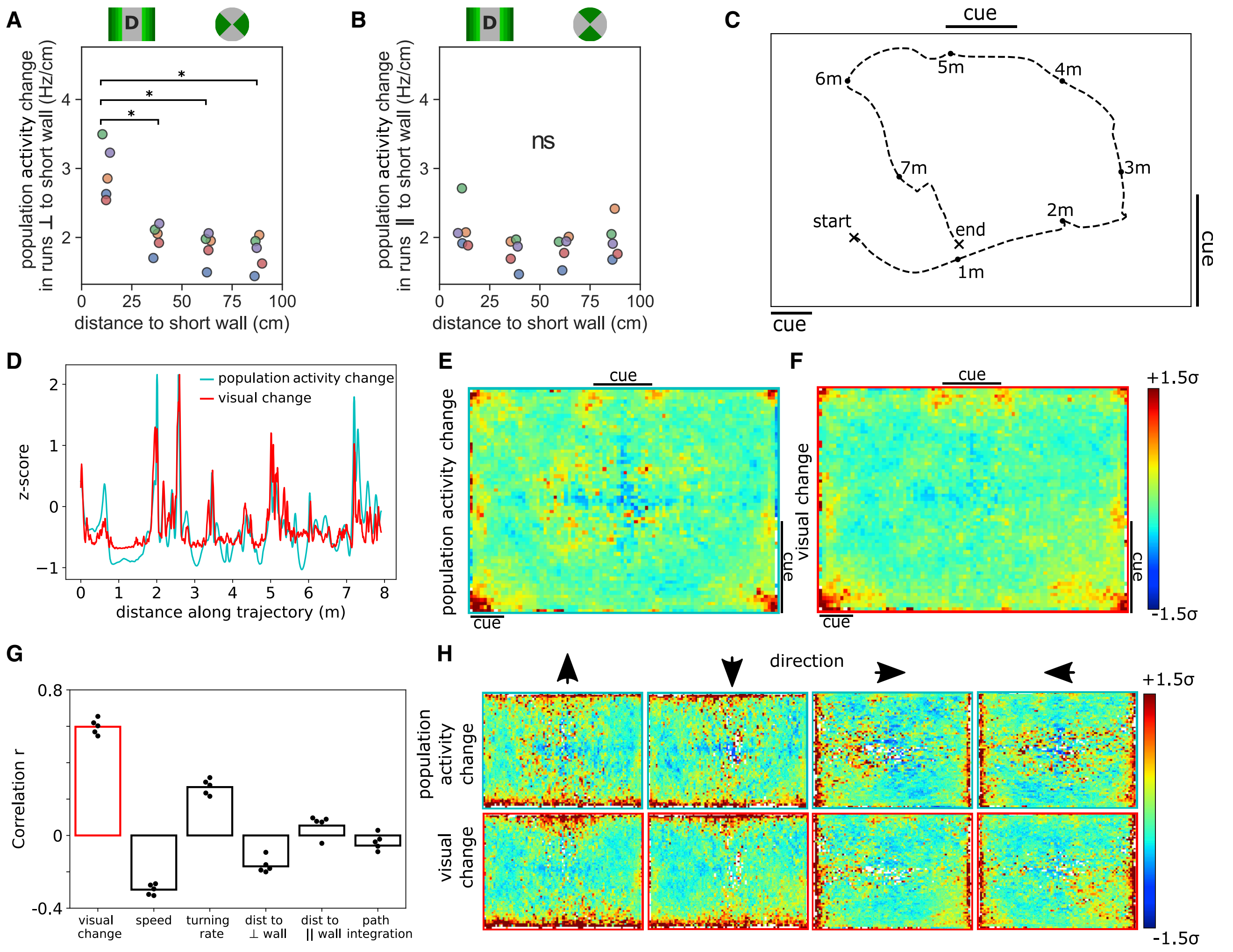

Figure 5. The rate of change in the place cell population mirrors the rate of change in the visual scene

Trajectories were split into 1-cm intervals, and the Euclidean distance between adjacent activity vectors was calculated for the largest environment (see STAR Methods on population activity change).

(A and B) For trajectories orthogonal to the wall (A), the rate of change in the place cell population was greater when animals were close to the wall—an effect that is not observed for trajectories running parallel to the wall (B). Pairwise post hoc tests adjusted for multiple comparisons using Benjamini-Hochberg (non-negative) correction. The cartoon above each plot indicates the wall distances and includes locations in the environment in green bands, and the green sectors on the circles indicate the sampled movement directions. The legend above indicates spatial bins (left) and movement directions (right) used to plot data. The same observations were made in the other large environments (Figures S5A and S5B). *p = 0.05; ns, not significant.

(C) An example 8-m trajectory in the largest environment from animal 5.

(D) Time series data for the trajectory in (C) showing a tight coupling between *Z* scored rate of change in the hippocampal population and reconstructed visual scene (Figure S5D).

(E and F) Spatially averaged rates of change for place cell population and visual scenes (data from all rats, no smoothing) are similar, both being accentuated toward walls, corners, and cues. Note the local increase at cue boundaries observed for both visual and population change (black lines in C indicate location of wall-mounted cues).

(G) Population activity change correlates more strongly (bars indicate correlation across all animals, points indicate correlations per animal) with visual change ($r = 0.60$) than speed ($r = -0.30$), turning rate ($r = 0.27$), distance to the nearest walls orthogonal ($r = -0.17$) or parallel ($r = 0.05$) to the rats' motion, and path integration since the rat last touched a wall ($r = -0.06$).

(H) Filtering by heading direction reveals how the change in both the visual scene and population activity depends on proximity to walls and their orientation relative to the direction of travel.

Our work resonates with early geometric cue-based models of place cell activity that sought to describe place fields as the threshold sum of putative boundary responsive neurons, evidence for which has been found in the subiculum and entorhinal cortex.[17–19] Notably, these models focused on the description of individual fields but were agnostic of population-level interactions.[20,21] Even so, geometric models generally assumed that boundary responses would be more diffuse for cells tuned to distant walls and that long-range boundary responses would be less common than short-range tuning.[17,19] Interestingly, early

formulations of these geometric models explicitly linked the tuning width of boundary responses to rate of change in the visual scene—specifically rate of change in the angle subtended to the top of boundary[40]—foreshadowing our results. Although these components yield spatial responses that broadly match our observations, it is unclear whether preconfigured boundary responses alone would be sufficient to generate a precise homeostasis. Thus, our current results likely indicate that a synthesis of both cell-level and population-level approaches is important for understanding how the hippocampus represents large-scale spaces—yoking the evolution of statistically stable population-level activity to movement through visual states.[24,41]

The precise mechanism that maintains the observed population-level statistics is unclear but might plausibly result from metabolic homeostasis imposed by limitations on blood flow, or the energetic limitations of the neurons themselves,[42] and could be modeled as competitive learning between hippocampal inputs for these limited resources.[43] Equally, it may well be a natural limitation of the highly recurrent CA3 network and the need to balance excitation with inhibition to avoid a runaway increase in activity.[44]

Recent publications have investigated the hippocampal map in larger environments,[45,46] with a focus on describing the variability of place field size.[11–13,47,48] A notable exception from these was a study describing how and why this distribution changes between locations, even claiming that there is no variability in place field size for small environments (i.e., no multiscale representation).[45] By recording across more than two differently sized environments, our results corroborate another study,[46] showing that there is a broad distribution of place field sizes in all environments and that larger environments allow for the mean of this distribution to increase, making the variability more prominent. Importantly, we extend this prior work, showing that it is precisely this change in the field size that can be explained by a simple mechanism—the rate of perceptual change afforded by the environment. We also corroborate findings from previous work that compared gamma-Poisson and equal-Poisson models in describing place cell field formation propensities on linear track and VR environments,[12,13] extending their findings to an open field environment.

Importantly, the relationship between visual change and place cell fidelity can be understood in terms of information theory. The rate of change of perceptual states is tightly linked to the amount of Fisher information they transmit about an unknown parameter[49]—in this case, position. Regions where the visual scene changes rapidly with respect to position—near the walls, for example—convey more information about self-location than regions where visual stimuli change more slowly. Indeed, previous work has shown that the spatial information conveyed by place cell activity is higher in the presence of cues,[30] with place fields closest to visual cues being the most informative.[50] In turn, for simple neural codes in one and two dimensions, the tuning width of a given neuron is inversely proportional to the Fisher information it carries.[25,26] If we make the reasonable assumption that the place code maximizes information transmitted up to the limit imposed by vision, then this directly predicts that place field width will be inversely proportional to the rate of change in the visual scene. Alternatively stated, the rate of change in place cell population activity is expected to be proportional to visual change—the result we observe. Notably, this relationship is not necessarily specific to vision or place cells. Thus, it seems plausible that the scale and fidelity of other neural representations of self-location must be subject to the same information-theoretic limits. Indeed, the increase in entorhinal grid cell scale noted toward the center of large environments,[51] and potentially other spatial distortions,[52,53] can be seen through the same lens.

STAR★METHODS

Detailed methods are provided in the online version of this paper and include the following:

- KEY RESOURCES TABLE
- RESOURCE AVAILABILITY
  - Lead contact
  - Materials availability
  - Data and code availability
- EXPERIMENTAL MODEL AND SUBJECT DETAILS
  - Animals and tetrode implantation
- METHOD DETAILS
  - Electrophysiological and behavioral recordings
  - Histology
  - Experimental paradigm
  - Cell identification
  - Computing ratemaps
  - Place field detection
  - Position decoding
  - Spatial correlation
  - Field formation models
  - Field size measures
  - Population activity statistics
  - Population activity change
  - Visual change
  - Path Integration
- QUANTIFICATION AND STATISTICAL ANALYSIS

SUPPLEMENTAL INFORMATION

Supplemental information can be found online at https://doi.org/10.1016/j.cub.2022.06.046.

ACKNOWLEDGMENTS

We would like to thank Tim Behrens, Dan Bush, Julie Lefort, and Romain Bourboulou for useful comments on the manuscript. S.T., W.d.C., and C.B. thank the Wellcome Trust for supporting this work through the Senior Research Fellowship awarded to C.B. (212281/Z/18/Z) and the Medical Research Council UK for the PhD studentship awarded to S.T. This research was funded in whole, or in part, by the Wellcome Trust (212281/Z/18/Z).

AUTHOR CONTRIBUTIONS

S.T. and C.B. conceptualized the experiment. S.T. collected and spike-sorted the data. S.T. and C.B. analyzed the electrophysiology and behavioral data. W.d.C. created the virtual replica, performed the simulations, and related analysis. S.T., W.d.C., and C.B. wrote the manuscript.

DECLARATION OF INTERESTS

The authors declare no competing interests.

Received: September 23, 2021
Revised: April 20, 2022
Accepted: June 15, 2022
Published: July 13, 2022

REFERENCES

1. O'Keefe, J., and Dostrovsky, J. (1971). The hippocampus as a spatial map. Preliminary evidence from unit activity in the freely-moving rat. Brain Res. *34*, 171–175.
2. Muller, R.U., Bostock, E., Taube, J.S., and Kubie, J.L. (1994). On the directional firing properties of hippocampal place cells. J. Neurosci. *14*, 7235–7251.
3. Skaggs, W.E., and McNaughton, B.L. (1998). Spatial firing properties of hippocampal CA1 populations in an environment containing two visually identical regions. J. Neurosci. *18*, 8455–8466.
4. O'Keefe, J., and Nadel, L. (1978). The Hippocampus as a Cognitive Map (Clarendon Press).
5. Ekstrom, A.D., Kahana, M.J., Caplan, J.B., Fields, T.A., Isham, E.A., Newman, E.L., and Fried, I. (2003). Cellular networks underlying human spatial navigation. Nature *425*, 184–188.
6. Yartsev, M.M., and Ulanovsky, N. (2013). Representation of three-dimensional space in the hippocampus of flying bats. Science *340*, 367–372.
7. Grosmark, A.D., and Buzsáki, G. (2016). Diversity in neural firing dynamics supports both rigid and learned hippocampal sequences. Science *351*, 1440–1443.
8. Kraus, B.J., Robinson, R.J., White, J.A., Eichenbaum, H., and Hasselmo, M.E. (2013). Hippocampal "time cells": time versus path integration. Neuron *78*, 1090–1101.
9. Ólafsdóttir, H.F., Carpenter, F., and Barry, C. (2017). Task demands predict a dynamic switch in the content of awake hippocampal replay. Neuron *96*, 925–935.e6.
10. Wilson, M.A., and McNaughton, B.L. (1993). Dynamics of the hippocampal ensemble code for space. Science *261*, 1055–1058.
11. Fenton, A.A., Kao, H.-Y., Neymotin, S.A., Olypher, A., Vayntrub, Y., Lytton, W.W., and Ludvig, N. (2008). Unmasking the CA1 ensemble place code by exposures to small and large environments: more place cells and multiple, irregularly arranged, and expanded place fields in the larger space. J. Neurosci. *28*, 11250–11262.
12. Lee, J.S., Briguglio, J.J., Cohen, J.D., Romani, S., and Lee, A.K. (2020). The statistical structure of the hippocampal code for space as a function of time, context, and value. Cell *183*, 620–635.e22.
13. Rich, P.D., Liaw, H.-P., and Lee, A.K. (2014). Large environments reveal the statistical structure governing hippocampal representations. Science *345*, 814–817.
14. Kjelstrup, K.B., Solstad, T., Brun, V.H., Hafting, T., Leutgeb, S., Witter, M.P., Moser, E.I., and Moser, M.-B. (2008). Finite scale of spatial representation in the hippocampus. Science *321*, 140–143.
15. Marr, D. (1982). Vision: A Computational Investigation into the Human Representation and Processing of Visual Information (W.H. Freeman and Company).
16. Bourboulou, R., Marti, G., Michon, F.-X., El Feghaly, E., Nouguier, M., Robbe, D., Koenig, J., and Epsztein, J. (2019). Dynamic control of hippocampal spatial coding resolution by local visual cues. eLife *8*, e44487.
17. Hartley, T., Burgess, N., Lever, C., Cacucci, F., and O'Keefe, J. (2000). Modeling place fields in terms of the cortical inputs to the hippocampus. Hippocampus *10*, 369–379.
18. O'Keefe, J., and Burgess, N. (1996). Geometric determinants of the place fields of hippocampal neurons. Nature *381*, 425–428.
19. Barry, C., Lever, C., Hayman, R., Hartley, T., Burton, S., O'Keefe, J., Jeffery, K., and Burgess, N. (2006). The boundary vector cell model of place cell firing and spatial memory. Rev. Neurosci. *17*, 71–97.
20. Káli, S., and Dayan, P. (2000). The involvement of recurrent connections in area CA3 in establishing the properties of place fields: a model. J. Neurosci. *20*, 7463–7477.
21. Samsonovich, A., and McNaughton, B.L. (1997). Path integration and cognitive mapping in a continuous attractor neural network model. J. Neurosci. *17*, 5900–5920.
22. de Cothi, W., and Barry, C. (2020). Neurobiological successor features for spatial navigation. Hippocampus *30*, 1347–1355.
23. Stachenfeld, K.L., Botvinick, M.M., and Gershman, S.J. (2017). The hippocampus as a predictive map. Nat. Neurosci. *20*, 1643–1653.
24. Uria, B., Ibarz, B., Banino, A., Zambaldi, V., Kumaran, D., Hassabis, D., Barry, C., and Blundell, C. (2020). The spatial memory pipeline: a model of egocentric to allocentric understanding in mammalian brains. Preprint at bioRxiv. https://doi.org/10.1101/2020.11.11.378141.
25. Zhang, K., and Sejnowski, T.J. (1999). Neuronal tuning: to sharpen or broaden? Neural Comput. *11*, 75–84.
26. Brown, W.M., and Bäcker, A. (2006). Optimal neuronal tuning for finite stimulus spaces. Neural Comput. *18*, 1511–1526.
27. Maurer, A.P., VanRhoads, S.R., Sutherland, G.R., Lipa, P., and McNaughton, B.L. (2005). Self-motion and the origin of differential spatial scaling along the septo-temporal axis of the hippocampus. Hippocampus *15*, 841–852.
28. Keinath, A.T., Epstein, R.A., and Balasubramanian, V. (2018). Environmental deformations dynamically shift the grid cell spatial metric. eLife *7*, e38169.
29. Fechner, G.T. (1860). Elemente der Psychophysik (Breitkopf u. Härtel).
30. Hetherington, P.A., and Shapiro, M.L. (1997). Hippocampal place fields are altered by the removal of single visual cues in a distance-dependent manner. Behav. Neurosci. *111*, 20–34.
31. Spiers, H.J., Hayman, R.M.A., Jovalekic, A., Marozzi, E., and Jeffery, K.J. (2015). Place field repetition and purely local remapping in a multicompartment environment. Cereb. Cortex *25*, 10–25.
32. Chen, G., Lu, Y., King, J.A., Cacucci, F., and Burgess, N. (2019). Differential influences of environment and self-motion on place and grid cell firing. Nat. Commun. *10*, 630.
33. Purandare, C.S., Dhingra, S., Rios, R., Vuong, C., To, T., Hachisuka, A., Choudhary, K., and Mehta, M.R. (2022). Moving bar of light evokes vectorial spatial selectivity in the immobile rat hippocampus. Nature *602*, 461–467.
34. Chen, G., King, J.A., Burgess, N., and O'Keefe, J. (2013). How vision and movement combine in the hippocampal place code. Proc. Natl. Acad. Sci. USA *110*, 378–383.
35. Jeffery, K.J., Donnett, J.G., Burgess, N., and O'Keefe, J.M. (1997). Directional control of hippocampal place fields. Exp. Brain Res. *117*, 131–142.
36. Anderson, M.I., and Jeffery, K.J. (2003). Heterogeneous modulation of place cell firing by changes in context. J. Neurosci. *23*, 8827–8835.
37. Quirk, G.J., Muller, R.U., and Kubie, J.L. (1990). The firing of hippocampal place cells in the dark depends on the rat's recent experience. J. Neurosci. *10*, 2008–2017.
38. Save, E., Cressant, A., Thinus-Blanc, C., and Poucet, B. (1998). Spatial firing of hippocampal place cells in blind rats. J. Neurosci. *18*, 1818–1826.
39. Wang, C.H., Monaco, J.D., and Knierim, J.J. (2020). Hippocampal place cells encode local surface-texture boundaries. Curr. Biol. *30*, 1397–1409.e7.
40. Burgess, N., Donnett, J.G., Jeffery, K.J., and O'Keefe, J. (1997). Robotic and neuronal simulation of the hippocampus and rat navigation. Philos. Trans. R. Soc. Lond. B Biol. Sci. *352*, 1535–1543.
41. Hedrick, K.R., and Zhang, K. (2016). Megamap: flexible representation of a large space embedded with nonspatial information by a hippocampal attractor network. J. Neurophysiol. *116*, 868–891.
42. Laughlin, S.B., de Ruyter van Steveninck, R.R., and Anderson, J.C. (1998). The metabolic cost of neural information. Nat. Neurosci. *1*, 36–41.

43. Barry, C., and Burgess, N. (2007). Learning in a geometric model of place cell firing. Hippocampus *17*, 786–800.
44. Hasselmo, M.E., Schnell, E., and Barkai, E. (1995). Dynamics of learning and recall at excitatory recurrent synapses and cholinergic modulation in rat hippocampal region CA3. J. Neurosci. *15*, 5249–5262.
45. Eliav, T., Maimon, S.R., Aljadeff, J., Tsodyks, M., Ginosar, G., Las, L., and Ulanovsky, N. (2021). Multiscale representation of very large environments in the hippocampus of flying bats. Science *372*, eabg4020.
46. Harland, B., Contreras, M., Souder, M., and Fellous, J.-M. (2021). Dorsal CA1 hippocampal place cells form a multi-scale representation of megaspace. Curr. Biol. *31*, 2178–2190.e6.
47. Alme, C.B., Miao, C., Jezek, K., Treves, A., Moser, E.I., and Moser, M.-B. (2014). Place cells in the hippocampus: eleven maps for eleven rooms. Proc. Natl. Acad. Sci. USA *111*, 18428–18435.
48. Park, E., Dvorak, D., and Fenton, A.A. (2011). Ensemble place codes in hippocampus: CA1, CA3, and dentate gyrus place cells have multiple place fields in large environments. PLoS ONE *6*, e22349.
49. Wei, X.-X., and Stocker, A.A. (2017). Lawful relation between perceptual bias and discriminability. Proc. Natl. Acad. Sci. USA *114*, 10244–10249.
50. Olypher, A.V., Lánský, P., Muller, R.U., and Fenton, A.A. (2003). Quantifying location-specific information in the discharge of rat hippocampal place cells. J. Neurosci. Methods *127*, 123–135.
51. Hägglund, M., Mørreaunet, M., Moser, M.-B., and Moser, E.I. (2019). Grid-cell distortion along geometric borders. Curr. Biol. *29*, 1047–1054.e3.
52. Krupic, J., Bauza, M., Burton, S., Barry, C., and O'Keefe, J. (2015). Grid cell symmetry is shaped by environmental geometry. Nature *518*, 232–235.
53. Stensola, T., Stensola, H., Moser, M.-B., and Moser, E.I. (2015). Shearing-induced asymmetry in entorhinal grid cells. Nature *518*, 207–212.
54. Barry, C., Hayman, R., Burgess, N., and Jeffery, K.J. (2007). Experience-dependent rescaling of entorhinal grids. Nat. Neurosci. *10*, 682–684.
55. Siegle, J.H., López, A.C., Patel, Y.A., Abramov, K., Ohayon, S., and Voigts, J. (2017). Open Ephys: an open-source, plugin-based platform for multichannel electrophysiology. J. Neural Eng. *14*, 045003.
56. Tanni, S., and Barry, C. (2021). SpatialAutoDACQ—Automated System for Scalable Data Acquisition in Spatial Tasks (Zenodo).
57. Kadir, S.N., Goodman, D.F.M., and Harris, K.D. (2014). High-dimensional cluster analysis with the masked EM algorithm. Neural Comput. *26*, 2379–2394.
58. Schmitzer-Torbert, N., and Redish, A.D. (2004). Neuronal activity in the rodent dorsal striatum in sequential navigation: separation of spatial and reward responses on the multiple T task. J. Neurophysiol. *91*, 2259–2272.
59. Harris, K.D., Hirase, H., Leinekugel, X., Henze, D.A., and Buzsáki, G. (2001). Temporal interaction between single spikes and complex spike bursts in hippocampal pyramidal cells. Neuron *32*, 141–149.
60. Mathis, A., Herz, A.V.M., and Stemmler, M. (2012). Optimal population codes for space: grid cells outperform place cells. Neural Comput. *24*, 2280–2317.
61. Towse, B.W., Barry, C., Bush, D., and Burgess, N. (2014). Optimal configurations of spatial scale for grid cell firing under noise and uncertainty. Philos. Trans. R. Soc. Lond. B Biol. Sci. *369*, 20130290.
62. Zhang, K., Ginzburg, I., McNaughton, B.L., and Sejnowski, T.J. (1998). Interpreting neuronal population activity by reconstruction: unified framework with application to hippocampal place cells. J. Neurophysiol. *79*, 1017–1044.
63. Virtanen, P., Gommers, R., Oliphant, T.E., Haberland, M., Reddy, T., Cournapeau, D., Burovski, E., Peterson, P., Weckesser, W., Bright, J., et al. (2020). SciPy 1.0: fundamental algorithms for scientific computing in Python. Nat. Methods *17*, 261–272.
64. Hardcastle, K., Ganguli, S., and Giocomo, L.M. (2015). Environmental boundaries as an error correction mechanism for grid cells. Neuron *86*, 827–839.
65. Benjamini, Y., and Hochberg, Y. (1995). Controlling the false discovery rate: a practical and powerful approach to multiple testing. J. R. Stat. Soc. B *57*, 289–300.
66. Seabold, S., and Perktold, J. (2010). Statsmodels: econometric and statistical modeling with Python. In 9th Python in Science Conference.

STAR★METHODS

KEY RESOURCES TABLE

REAGENT or RESOURCE	SOURCE	IDENTIFIER
**Chemicals, peptides, and recombinant proteins**		
Cresyl violet	Sigma Aldrich	Product code: C5042, http://www.sigmaaldrich.com/catalog/product/sigma/c5042?lang=en&region=US
Histoclear	National Diagnostics	Product code: HS-202, https://www.nationaldiagnostics.com/histology/product/histo-clear-ii
**Deposited data**		
Supporting data for: State transitions in the statistically stable place cell population are determined by rate of perceptual change	figshare	Figshare: https://doi.org/10.5522/04/18128891.v1
**Experimental models: Organisms/strains**		
Lister Hooded rats	Charles River	http://www.criver.com/products-services/basic-research/find-a-model/lister-hooded?loc=GB
**Software and algorithms**		
Custom Python code	Zenodo	Zenodo: https://doi.org/10.5281/zenodo.5842287
Unity3D	https://unity3d.com/get-unity/download	2019 LTS
Tint Spike sorting software	Axona	Product code: COMP/TINT01, http://axona.com/products
**Other**		
Single-screw mouse microdrive	Axona	Product code: MDMR-01M1, http://axona.com/products
NanoZ plating equipment	Multichannel Systems	nanoZ, http://www.multichannelsystems.com/products/nanoz
Open EPhys Acquisition Board	Open Ephys Organisation	https://open-ephys.org/acquisition-system/eux9baf6a5s8tid06hk1mw5aafjdz1
Intan Standard SPI interface cable	Intan Technologies	RHD2000 3-ft (1.8 m), https://intantech.com/products_RHD2000.html
Intan amplifier/accelerometer board with 64 unipolar inputs	Intan Technologies	RHD2164, https://intantech.com/products_RHD2000.html
32 channel connector, Omnetics	Genalog	A79621-001, https://genalog.com/
12.7 µm HM-L coated Stablohm 650	California Fine Wire Company	100187, http://www.calfinewire.com
17 µm H HL coated platinum-iridium	California Fine Wire Company	100167, http://www.calfinewire.com

RESOURCE AVAILABILITY

Lead contact

Further information and requests for resources and reagents should be directed to and will be fulfilled by the lead contact, Caswell Barry (caswell.barry@ucl.ac.uk).

Materials availability

This study did not generate new unique reagents.

Data and code availability

- All data have been deposited at figshare (UCL Research Data Repository) and are publicly available as of the date of publication. DOIs are listed in the key resources table.
- All original code has been deposited at Zenodo and is publicly available as of the date of publication. DOIs are listed in the key resources table.
- Any additional information required to reanalyze the data reported in this paper is available from the lead contact upon request.

EXPERIMENTAL MODEL AND SUBJECT DETAILS

Animals and tetrode implantation

Five male Lister Hooded rats were used for this study. All procedures were approved by the UK Home Office, subject to the restrictions and provisions contained in the Animals Scientific Procedures Act of 1986. All rats (333-386 g/13-17 weeks old at implantation) were implanted with two microdrives targeted to the right and left CA1 (ML: 2.5 mm, AP: 3.8 mm posterior to bregma, DV: 1.6 mm from dura) following a standard surgery and recovery procedure.[54] After surgery, rats were housed individually in Perspex cages (70 cm long x 45 cm wide x 30 cm high) on a 12 hr light/dark cycle. Screening and experiments took place during the dark phase of the cycle. After one week of recovery, rats were maintained at 90-95% of free-feeding weight with ad libitum access to water.

The hair around the incision site was removed, and the skin was sterilized with Betadine. The animal was placed on a heating pad for the duration of the surgery to maintain body temperature. Viscotears Liquid Gel was used to protect the animal's eyes. General anesthesia during the operation was maintained with an isoflurane-oxygen mix of 1.5-3% at 3 l/min. Carprieve (1:10) and Baytril were injected subcutaneously (0.1 ml/100 g) before the surgery for analgesia and to minimize chances of infection, respectively. Baytril was also included in post-operative treatment in their water for one week. An analgesic, Metacam Oral Suspension suspended in jelly, was administered for three days post-surgery. The tetrodes were implanted through ~1 mm trephine craniotomies over target sites, and they were fixed to the exposed skull with dental cement (Super-Bond C&B) and six bone screws. A gold pin used as ground and reference was soldered to one of the orbital bone screws before its implantation. The craniotomies and elements of the microdrives were protected from dental cement using Vaseline.

METHOD DETAILS

Electrophysiological and behavioral recordings

Each single-screw microdrive (Axona) was assembled with two 32 channel Omnetics connectors (A79026-001), 16 tetrodes of twisted wires (either 17 μm H HL coated platinum-iridium, 90% and 10% respectively, or 12.7 μm HM-L coated Stablohm 650; California Fine Wire), and platinum-plated to reduce impedance to below 150 kΩ at 1 kHz (NanoZ).

Electrophysiological recordings were acquired using Open Ephys recording system[55] and a 64-channel amplifier board per drive (Intan RHD2164). The recorded signal was referenced to an orbital bone screw - also the ground for the amplifier boards. The Open Ephys Acquisition Board was grounded to an aluminum foil sheet positioned underneath the vinyl flooring throughout the entire extent of the experimental room. Electrophysiological signals were recorded from 128 channels at 30 kHz. Spikes were detected as negative threshold crossings of more than 50 μV in the 30 kHz signal after bandpass filtering between 600 and 6000 Hz. For each spike, waveforms were stored at 30 kHz for the 1.2 ms window surrounding the threshold crossing. The waveforms are displayed and discussed in their inverted form, where the largest deflection from baseline is a positive peak.

Positional tracking was performed with an open-source multi-camera tracking system SpatialAutoDACQ.[56] The output position data from SpatialAutoDACQ was the spatial coordinate of an infra-red LED positioned above the animal's ears, sampled at 30 Hz.

Histology

Anatomical locations of recordings were verified using histology. Rats were anesthetized with isoflurane and given intraperitoneal injection of Euthatal (sodium pentobarbital) overdose (0.5 ml / 100 g) after which they were transcardially perfused with saline, followed by a 10% Formalin solution. Brains were removed and stored in 10% Formalin and 30% sucrose solution for 3-4 days before sectioning. Subsequently, 50 μm frozen coronal sections were cut using a cryostat, mounted on gelatin coated or positively charged glass slides, stained with cresyl violet and cleared with clearing agent (Histo-Clear II), before covering with DPX and coverslips. Sections were then inspected using an Olympus microscope, and tetrode tracks reaching into CA1 pyramidal cell layer were verified.

Experimental paradigm

Screenings for a suitable place cell yield were performed from one week after surgery in a 1.4 x 1.4 m environment, different from those used in any of the experiments. Tetrodes were gradually advanced in 62.5 μm steps until ripple oscillations could be observed, and pyramidal cells with stable firing fields could be identified.

Screenings, training, and experiments all took place in the same experimental room, using environments constructed of the same materials. Environments had black vinyl flooring; were constructed of 60 cm high modular boundaries (MDF) colored matt black, surrounded by black curtains on the sides and above. Each environment was illuminated by an elevated (2 m) diffuse daylight lamp from each corner of the environment that was adjacent to a corner of the experimental room (multiple lights in larger environments), with each lamp producing between 30-50 Lux/m. All experiments involved scattered 20 mg chocolate-flavored pellets (Dustless Precision Pellets Rodent, Purified, Bio-Serv, USA) dropped into the environment by an automated system in SpatialAutoDACQ to encourage foraging. The automated system scattered the pellets randomly with greater preference for areas least visited by the animal.

Place cells were recorded as each animal foraged in four environments of different size (Figures 1A and S1A). The environment sizes were 87.5 x 125 cm (environment A), 175 x 125 cm (environment B), 175 x 250 cm (environment C) and 350 x 250 cm (environment D). All environments were rectangles with close to identical shape (axes ratio 1.40). Environment A was the smallest, and the other sequentially larger environments – B, C and D – each doubled in size by doubling the length of the shortest axis (Figure 1A). There were two sets of cues in the environments. The most prominent cue elevated above the wall of the enclosure was different

in all environments, varying in size and the type of pattern, but always black and white. Two different secondary smaller cues were used, an A4 sheet (11 × 16 cm) with a dot pattern and a set of three adjacent A4 pages, placed on the wall at a height the animal could not reach. The number of secondary cues and the size of the primary cues varied slightly between environments to scale with their size (Figure 1A).

During a single session consisting of 5 trials, the place cells of an animal were recorded in all four environments and twice in environment A (Figure 1B). The second recording in environment A is referred to as a recording in environment A'. The duration of the recording in the smallest environment (A) was 15 minutes. The recording duration doubled along with the size of each environment, reaching 120 minutes in the largest environment (D). Each animal was recorded on three or four sessions, the data analyzed here comes from the first session in which an animal achieved good spatial sampling in all environments (sessions 3, 2, 2, 3, and 4, for the 5 animals).

After a recording in each environment, the flooring was wiped with an unscented soap solution to clear any potential olfactory cues. The animal was kept in a familiar rest box (with water provided) between each recording for 15 to 30 minutes, while the preceding and following environments were disassembled and reassembled, respectively.

Cell identification

Spikes were assigned unit identities with automated clustering software (KlustaKwik)[57] based on spike waveforms. The results from the automated clustering were curated using an offline data analysis suite (Tint, Axona, St. Albans, UK) to further separate under-clustered units and merge over-clustered units.

All recordings from a given animal that were performed on the same session were clustered simultaneously, concatenating the spike waveform data. Therefore, the same set of units were identified across all such recordings. This approach made it possible to analyze the properties of the same place cell population in multiple conditions.

L-ratio[58] and Isolation Distance[59] were calculated through Mahalanobis distance to verify that sorting quality has not affected the results. The features used for this analysis were the same as those used for automated and manual clustering: amplitude, time-to-peak, time-to-trough, peak-to-trough, half-width, trough-ratio, and the first three PCA components of waveforms. These measures were computed on waveforms combining all clusters, including noise, and pooling across all recordings – the same way as was done for spike sorting.

Place cells were identified computationally after the clustering procedure. The following criteria were used to identify place cells:

- Waveform peak-to-trough duration of over 0.45 ms.
- Waveform peak half-width of over 0.1 ms.
- The ratio between amplitude and trough voltage values (trough-ratio) of over 0.175.
- Spatial correlation of odd and even minute ratemaps of over 0.5 in at least one recording.
- Spatial correlation of first and last half ratemaps of over 0.25 in at least one recording.
- At least one field in one of the recordings (field detection method described below).
- Mean firing rate across all recordings lower than 4 Hz.

Place cells were further filtered for duplicates recorded on separate tetrodes. Duplicate units were considered to be unit pairs that passed the following criteria mostly based on cross-correlograms with 2 ms bins and a maximum lag of 25 ms:

- At least 200 spikes at 0-lag.
- Lower than 0.5 ms sigma of a gaussian fitted to the cross-correlogram.
- Mean spatial correlation of ratemaps higher than 0.5 across recordings where both units have at least 200 spikes.

If duplicate units were detected, the one with more total spikes was set as noise, to maximize signal to noise ratio. This approach was based on the observation that the unit in the duplicated pair that had more spikes was usually less well isolated from noise or other units.

Interneurons were identified based on the following criteria:

- Minimum mean firing rate of 4 Hz across all recordings.
- Maximum waveform half-width of 150 μs.
- Maximum trough-ratio of 0.4.
- Maximum spatial correlation of 0.75 in any environment.

Computing ratemaps

To calculate a ratemap for each unit, the position data was binned into 4 cm square bins, and the number of position samples in each bin was divided by the sampling rate, producing the dwell time for each spatial bin. Spike timestamps were paired with simultaneous position samples and assigned to corresponding spatial bins, thereby producing spike counts for each spatial bin. Only the position samples and spike timestamps from periods where the animal was moving at more than 10 cm/s were used to produce these dwell time and spike count maps. Both dwell times and spike counts were smoothed with a Gaussian kernel (standard deviation of 2 spatial

bins) while setting unsampled bin values and those outside the environment to 0. The resulting smoothed spike counts were divided by smoothed dwell times, producing spatial ratemaps.

Place field detection

Place fields were detected in spatial ratemaps to analyze place cell properties at the level of individual place fields. Here, a place field is defined as a contiguous area in a ratemap, where the firing rate decays continuously from a single prominent peak, and the observed firing rates are well correlated across multiple visits to the same location. Often individual place fields are so close to each other that the firing rate threshold traditionally used for place field detection (1 Hz) would not be able to detect them as separate place fields. This effect is exacerbated by the spatial smoothing step in computing spatial ratemaps. However, based on the definition above, these areas should be considered as separate place fields.

An iterative thresholding method was used to find spatial bins that constituted a single place field in a ratemap (Figure S1B). As a first step, the ratemap was thresholded at 1 Hz, and contiguous groups of bins (ignoring diagonal connections) were identified as candidate fields. The regions including at least 10 spatial bins and a peak value of at least 2 Hz were considered as valid candidate fields. The ratemap of each valid candidate field was then thresholded again with a 0.05 Hz higher threshold (1.05 Hz), and the same method of finding contiguous regions and their validation was applied. This was done iteratively, resulting in continuously smaller regions, each with a higher threshold and associated with their parent field candidates with a lower threshold, some having more than one child field candidate.

The resulting lists were then parsed in reverse order, starting with the smallest candidate fields with highest thresholds. Candidate fields that were too large (greater than half the bins of the ratemap) or not sufficiently stable over repeated visits to the region (spatial correlation of odd and even minute ratemaps below 0.25) were ignored. As the increasingly lower threshold candidate fields overlapping with each other were assessed, the lowest threshold valid candidate field in a sequence of overlapping candidate fields was detected as a place field. The overlapping candidate fields with a higher threshold were ignored. If more than one child candidate field of a lower threshold candidate field was valid, the large single candidate field was ignored, and the smaller valid candidate fields were detected as separate place fields. In this manner, multiple place fields were detected in individual ratemaps of single units, as illustrated in Figure S1B.

Position decoding

Position decoding was used to estimate the location encoded in the activity of a place cell population at specific timepoints. The probability of the animal being at each location in the environment is computed based on the similarity between the ongoing firing rates of place cells in a time-window (e.g. 1 second) and their spatial ratemaps. The decoded location is then identified as the one with the highest likelihood. The spatial ratemaps for this purpose were computed using periods where the animal was moving faster than 10 cm/s, excluding the time-point that was being decoded – cross-validation with a 3-minute window. The method of matching the ongoing population activity to spatial ratemaps has been used previously[60,61] and is based on the original formulation by Zhang et al.[62]

Specifically, the population activity of N units $K = (k_1, ..., k_N)$ was computed, where k_i is the spike rate of the i-th unit in a temporal bin (e.g. 1 second). Expected population activity a at location x, belonging to the set of all spatial ratemap bin centres X, was based on the values of all units in the spatial ratemap corresponding to that location bin $a(x) = (f_i,...,f_M)$, such that $a_i(x) = f_i$, refers to the value in the spatial ratemap of unit i at location x. These representations of neural activity were used to compute the conditional probability of observing K, at location x as:

$$P(K|x) = \prod_{i}^{N} \frac{a_i(x)^{k_i}}{k_i!} e^{-a_i(x)} \quad \text{(Equation 1)}$$

This method allows assessing the probability of any spatial bin being decoded independently of the number of bins considered and their spatial arrangement. It is agnostic to the animal's real location and past decoded locations, as it considers all locations to have equal prior probability – it has a flat prior. The location encoded in the population activity $\widehat{x}(K)$ was then computed as the centre of the spatial bin with the highest conditional probability:

$$\widehat{x}(K) = \max_{x \in X} P(K|x) \quad \text{(Equation 2)}$$

To decode the environment from the place cell population activity (Figure S2B), the posterior probability distribution (Equation 1) was calculated over all visited bins in all environments. The environment pertaining to the most probable spatial bin (Equation 2) was then identified as the decoded environment.

Spatial correlation

Spatial correlation was used to quantify the similarity between spatial ratemaps of pairs of cells or spatial ratemaps of the same cell that were constructed using data from different parts of the same recording. Spatial correlation was the Pearson correlation coefficient for pairs of values from spatial bins with matching locations in two ratemaps. For a spatial bin to be included, it must have had a firing rate above 0.01 Hz in at least one of the ratemaps to avoid high correlations between 0 Hz bins. Unvisited bins were ignored. At least 6 such valid spatial bins were required for spatial correlation to be computed, which was always the case for place cells.

Field formation models

Two models were used to estimate place field count per cell as a function of environment size: equal-Poisson and gamma-Poisson models. The equal-Poisson model has one parameter, the average field formation propensity τ, which is constant for all cells, and the model predicts the field counts per cell X as a function of τ and environment area in m^2 A

$$X \sim Pois(\tau A) \quad \text{(Equation 3)}$$

The gamma-Poisson model estimates the field formation propensities T in the place cell population based on a shape α and scale θAparameters

$$T \sim Gamma(\alpha, \theta A) \quad \text{(Equation 4)}$$

These field formation propensities T are then used to estimate the place field counts X

$$X \sim Pois(T) \quad \text{(Equation 5)}$$

Gamma-Poisson can then be defined using a negative binomial

$$X \sim negbin\left(\alpha, \frac{1}{1+\theta A}\right) \quad \text{(Equation 6)}$$

Using the change of variables $r=\alpha$, $p = \frac{1}{1+\theta A}$

$$X \sim negbin(r, p) \quad \text{(Equation 7)}$$

giving the gamma-Poisson probability mass function

$$P(X = x) = \frac{\Gamma(r+x)}{\Gamma(r)\Gamma(x+1)} p^r (1 - p)^x \quad \text{(Equation 8)}$$

The parameters for both models were optimized using maximum likelihood estimation using the field counts of all place cells (N = 627) in the combined environment - counting fields per cell across all four environments. The parameter optimization was performed with L-BFGS-B solver implemented in the Scipy Python package.[63] The two models were compared using the Bayesian information criterion.

The gamma-Poisson model was fit as described above, and then evaluated on prediction of proportion of cell recruitment with the physical environment size values and also with environment sizes adjusted based on field density. The latter always performed better, therefore, all the reported results were computed using environment sizes adjusted based on field density. The proportion of fields in each environment was modeled with linear regression $y = bA + c$, where A is the area of an environment (Figure 2E). Therefore, using the same parameters b and c the field density ρ in an environment of size A can be computed as

$$\rho = \frac{bA+c}{A} \quad \text{(Equation 9)}$$

The field density adjusted environment size A' for computing the gamma-Poisson probability mass function in an environment with size A was computed as

$$A' = A\frac{\rho}{b} \quad \text{(Equation 10)}$$

Proportion of place cells recruited to form at least one field in the four environments (A, B, C and D) based on the gamma-Poisson model was computed by modelling a population of 100,000 cells. Each cell had a field formation propensity τ (drawn from the gamma distribution defined by α and θ fit already previously to field formation propensities) that was used to compute the number of fields using a Poisson process with rate τA or $\tau A'$. Modelled cells with no field in any of the environments were ignored to match the conditions applied to the experimental data.

Field size measures

The place field areas were computed by summing the area of all spatial bins (16 cm^2) covered by each detected field. The place field widths in the two axes were computed as the length of the field's projection onto a given axis (Figure S3B). These values were used to calculate the mean field area and width in each animal at every spatial bin by averaging the values of all fields overlapping a particular spatial bin. To estimate the average field area and width at different distances to the wall, the values for spatial bins in a particular range of distance from the wall (e.g. 0-25 cm) were averaged separately for each animal. Where further spatial selection is indicated in the cartoons above figures (e.g. only including data from the middle third of the environment), the averaged spatial bins were selected in such manner to minimize the effects from orthogonal walls.

Population activity statistics

The proportion of co-active place cells and the mean firing rate of place cells at different distances to the wall were computed by averaging the spatial ratemap values in all spatial bins that were in that range of distances from the wall. The proportion of co-active

cells (firing rate $\geq$1 Hz) and the mean firing rate was computed using the spatial ratemap values at a given location, including all place cells detected in a given animal.

The population firing rate distributions were computed by aggregating the Gaussian smoothed (1 second sigma) firing rates aligned to position samples (30 Hz), across animals, where the animals' location was within a particular range of distance from the wall. At each timepoint, only the activity of neurons with firing rate $\geq$1 Hz were included to facilitate measuring firing rate distributions. Only samples when the animal was moving faster than 10 cm/s were included. The samples assigned to each range of distances from the wall were further split temporally into the first and second half. The Jensen-Shannon divergence, a measure of similarity between two distributions, was then computed between each pair of temporal halves, to quantify inherent variation, and also between all halves at different distances to the wall, to quantify variation in the firing rate distributions arising from difference in the animals' position.

Population activity change

Position data smoothed with Savitzky–Golay filter (166 ms window and polynomial order 5) and place cell firing rates computed at 33 ms bins and smoothed with a Gaussian (166 ms sigma) were used to construct population activity vectors for computing the population activity change. The position data was reduced to one dimension by computing the Euclidean distance cumulatively over consecutive samples. It was then used to linearly interpolate cell firing rates to position samples 1 cm apart. The population activity change was then computed as the Euclidean distance between consecutive samples of place cell firing rates - population activity vectors measured at 1 cm intervals. All place cells detected in an animal were included and periods where the animal was moving slower than 10 cm/s were excluded.

Population activity change was used in place of a direct rate map analysis as it is less susceptible to issues caused by 'cut-off' place fields at boundaries (Data S1B) and intra-trial remapping (Data S1A). To explain why we performed a series of simulations. First, we simulated N=1271 equal-sized Gaussian place fields distributed to evenly span the largest space used in the study. Thus the centers of some place fields lay outside of the walls, and so were not accessible to the 'rat', giving rise to 'cut off' place fields (Figure S5E). In these cases the effective peak of the field lay against the wall. Despite this, and in accordance with our results, the total population activity of the simulated place cells is the same at every measurable position in the environment (Figure S5F, top). This also means that the measurable rate map peaks, confined by the enclosure, are more densely distributed at the boundaries. Most importantly, however, the rate of change in the place cell population vector is constant everywhere (Figure S5F, bottom) due to the uniform level of overlap between fields in the measurable space - a result that holds true irrespective of heading direction (Figure S5G). Conversely, if instead of using population measures we were to just examine the size of individual place fields, then these would necessarily appear to be smaller near to the walls because of the cut off portions. For these reasons we prefer the population-level measures.

Next, we adapted our simulation to capture the main elements of our experimental findings (Figure S5H): 1) Place field centers are more densely distributed closer to boundaries (here we use a $1/x^2$ scaling for field density, abstracted from the derivative of the angle to the top of a boundary wall that is distance x away) 2) Field width orthogonal to the boundary follows the inverse scaling (i.e. scales with x^2) such that 3) The total population firing is the same everywhere (Figure S5I, top). Here we see that now the rate of change in the population vector is greater near the boundaries of the environment (Figure S5I, bottom), and in particular this is mainly the case when travelling orthogonally to those boundaries (Figure S5J). Note, the predictions of this model are directly in line with our main results (Figure 5) - although this modelling does not take into account the position of the visual cues in the environment which can be seen to also have a strong effect on the rate of change in the population vector (Figures 5G and 5H - high rate of change is visible at the edges of the visual cue on the North wall).

Visual change

The virtual environment was created in Unity3D with the same proportions as the physical environment. Animal trajectories were speed filtered (>10 cm/s) and interpolated so that consecutive samples were 1 cm apart (equivalent to method in population activity change). The visual scene from each sample point was then captured by three greyscale cameras, raised the equivalent of 5 cm from the floor and angled 35° above the horizontal axis. These cameras were oriented 100° apart in the horizontal plane, and each rendered a 64x64 pixel image with a field of view of 100° to give a total field of view of 300°. The absolute difference between consecutively sampled greyscale images was used to yield the pixel-by-pixel change at each sampling point. This pixel-by-pixel change was then z-scored per pixel and averaged across pixels to generate a single value for visual change at each sample point.

Visual change and population activity change maps represent the average value of samples across the environment using a 4x4 cm bin size and no smoothing applied. For illustration purposes, the time series presented in Figure 5D was smoothed with a 1D boxcar filter of width 3, but no smoothing was applied when calculating the reported correlations between time series.

Path Integration

Points of contact with a particular wall were determined as the animal being within 12cm of it.[64] The path integration variable for a given sampling point in the trajectory was then taken as the cumulative distance travelled along the trajectory since the last point of contact with a wall.

QUANTIFICATION AND STATISTICAL ANALYSIS

The details of statistical analysis - test statistics, p value, and sample size (N) - can be found in figure legends or the relevant parts of the results section. Any exclusion of data is detailed in the results section and figures.

In most cases Kruskal-Wallis test was used to test for differences between groups due to small sample sizes. Positive Kruskal-Wallis tests of more than two samples were followed by two-sided Mann-Whitney U test for individual pairwise comparisons, and Benjamini/Hochberg (non-negative) correction[65] for multiple comparisons. Benjamini/Hochberg (non-negative) correction was implemented in Statsmodels Python package[66] and Kruskal-Wallis, Mann-Whitney U, Linear regression, Pearson coefficients, Poisson and Gamma distributions were computed using Python statistics package Scipy.[63]

Current Biology, Volume *32*

Supplemental Information

State transitions in the statistically stable place cell population correspond to rate of perceptual change

Sander Tanni, William de Cothi, and Caswell Barry

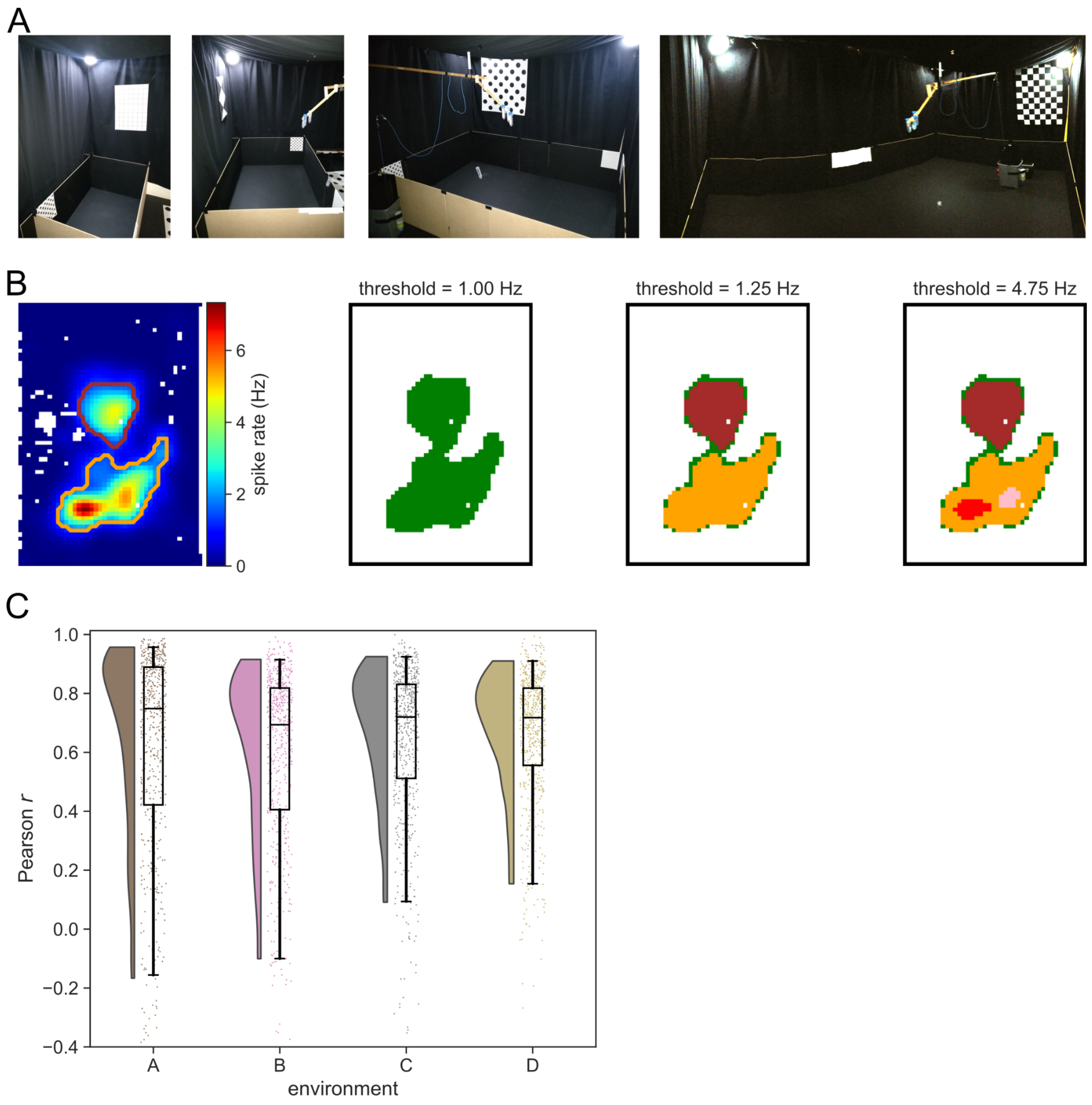

Figure S1. Experimental paradigm description and validation. Related to main Figure 1. (**A**) Photographs of experimental environments (from left: A, B, C and D). (**B**) Field detection method based on iterative thresholding identifies multiple place fields in a single ratemap. The spatial ratemap of a place cell is shown on the left. With the threshold at 1 Hz, only one place field (green) is detected. While increasing the threshold at 0.05 Hz increments, two place fields (brown and orange) are identified with 1.25 Hz threshold. These both pass the place field criteria. By increasing the threshold further, two smaller place fields (red and pink) are detected with a 4.75 Hz threshold, both overlapping with the larger orange place field. At least one of the smaller place fields (red and pink) did not pass the place field criteria. Therefore, both of them (red and pink) were ignored because a larger place field, detected with a lower threshold and overlapping with them, did pass the place field criteria. (**C**) Spatial correlation between first and second half of the recording in each environment for every active place cell. The box shows quartiles of the dataset, and whiskers indicate the 5th and 95th percentile of the data distribution.

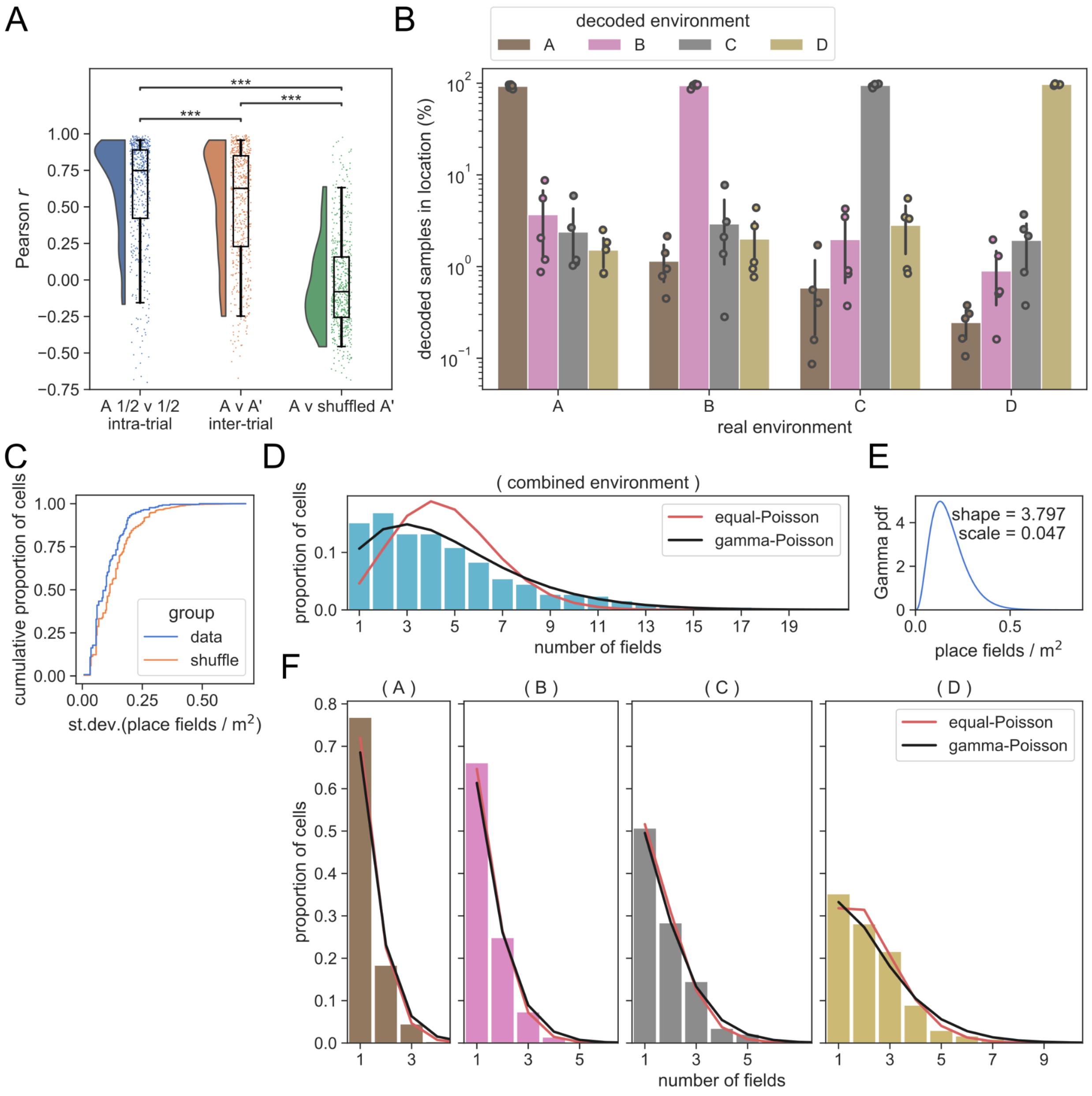

Figure S2. Details of recorded place cell and place field counts. Related to main Figure 2. (**A**) Place cells formed stable representations. Spatial correlations (bin-wise Pearson correlation) between ratemaps from the first and second half of environment A (A ½ v ½) and between repeated trials (A v A') were high (mean spatial correlation of 0.61 and 0.51, respectively), significantly exceeding the values obtained by randomly repairing cells (A v shuffled A') (-0.04). Kruskal-Wallis test: H = 644, p = 1.64 x 10^{-140}; Mann-Whitney for A ½ v ½ and A v A', U = 1.8 x 10^{5}, p = 3.2 x 10^{-6}, for A ½ v ½ and A v shuffled A', U = 2.8 x 10^{5}, p = 10^{-92}, for A v A' and A v shuffled A', U = 2.9 x 10^{5}, p = 10^{-114}. The box shows quartiles of the dataset, and whiskers indicate the 5th and 95th percentile of the data distribution. P-value markers: ***, 0.001. The kernel-density estimate is bounded between the 5th and 95th percentile. (**B**) Place cells formed distinct representations for each environment. Population activity vectors were reliably decoded to the environment from which they were drawn - Bayesian-framework with 1s window used for decoding. Error bars show 95% confidence intervals of the mean based on bootstrapping. (**C**) Place cell field formation propensity is conserved across environments. The standard deviation of place cells' field formation propensity (place fields / m^2, accounting for field density in

Figure 2E inset) across environments was lower than for a shuffled distribution (Mann-Whitney: $U = 2.7 \times 10^7$; $p = 6 \times 10^{-8}$; $n = 258$). Only cells with at least 1 field in each environment were used in this analysis. Shuffle was obtained by permuting cell identities within each animal and environment 1000 times. (**D**) Distribution of field counts per place cell after grouping all environments together and predictions of the two models fit to this data. (**E**) Probability density function (pdf) of gamma with fitted parameters, defining the field propensity distribution as a function of environment size. (**F**) Distribution of field counts per place cell for cells in each environment that have at least one place field, and predictions of the two models fit to data in **D**.

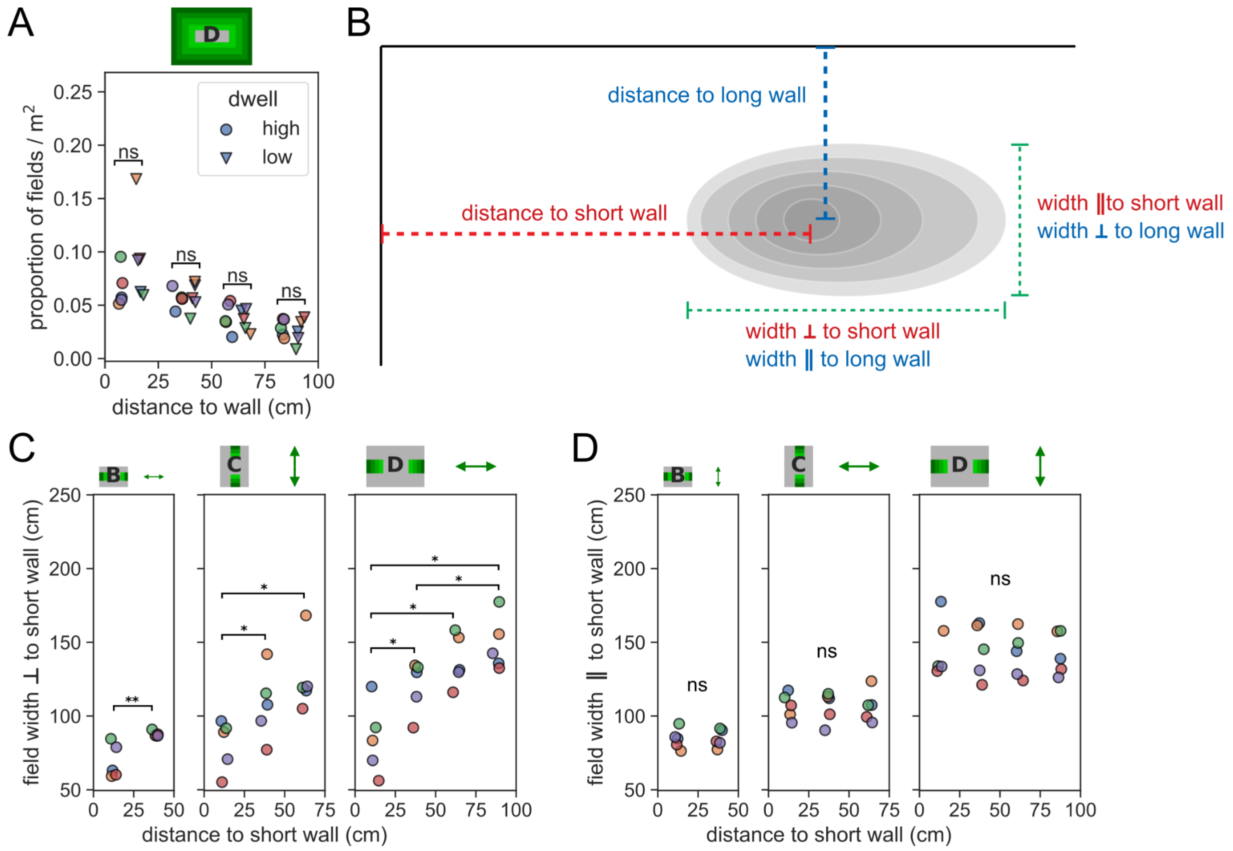

Figure S3. Variability in place field density and size. Related to main Figure 3. (**A**) Change in field density with distance to wall is not explained by the difference in dwell time. Field density (proportion of fields / m^2) and dwell time were computed for 25 x 25 cm non-overlapping regions of the largest environment chosen to be at a range of distances to the nearest wall. For each animal, we grouped regions according to distance to the wall and for each of these groups found the mean field density in the region with the highest and lowest dwell time. Field density did not vary with dwell time (Mann-Whitney for all comparisons: $U \geq 6$, $p \geq 0.11$) but was different between regions at different distances to the wall (Kruskal-Wallis for both high and low dwell: $H \geq 13.1$, $p \leq 0.004$). P-value markers: *, 0.05; **, 0.01; ns, not significant. (**B**) The width of each place field was measured along two orthogonal axes. The distance of the place field from nearest walls in the two axes was measured from the location of peak firing rate. (**C**) The average place field size per animal measured orthogonal to the short wall increases with distance from the wall and is also greater near the wall in the larger environments. Pair-wise post hoc tests adjusted for multiple comparisons using Benjamini/Hochberg (non-negative) correction. The cartoon above the plot indicates the wall distances and included locations in the environment in green, and the arrow indicates the axis of measurement. (**D**) The average place field size per animal measured parallel to the short wall is constant at all distances to the short wall in all environments.

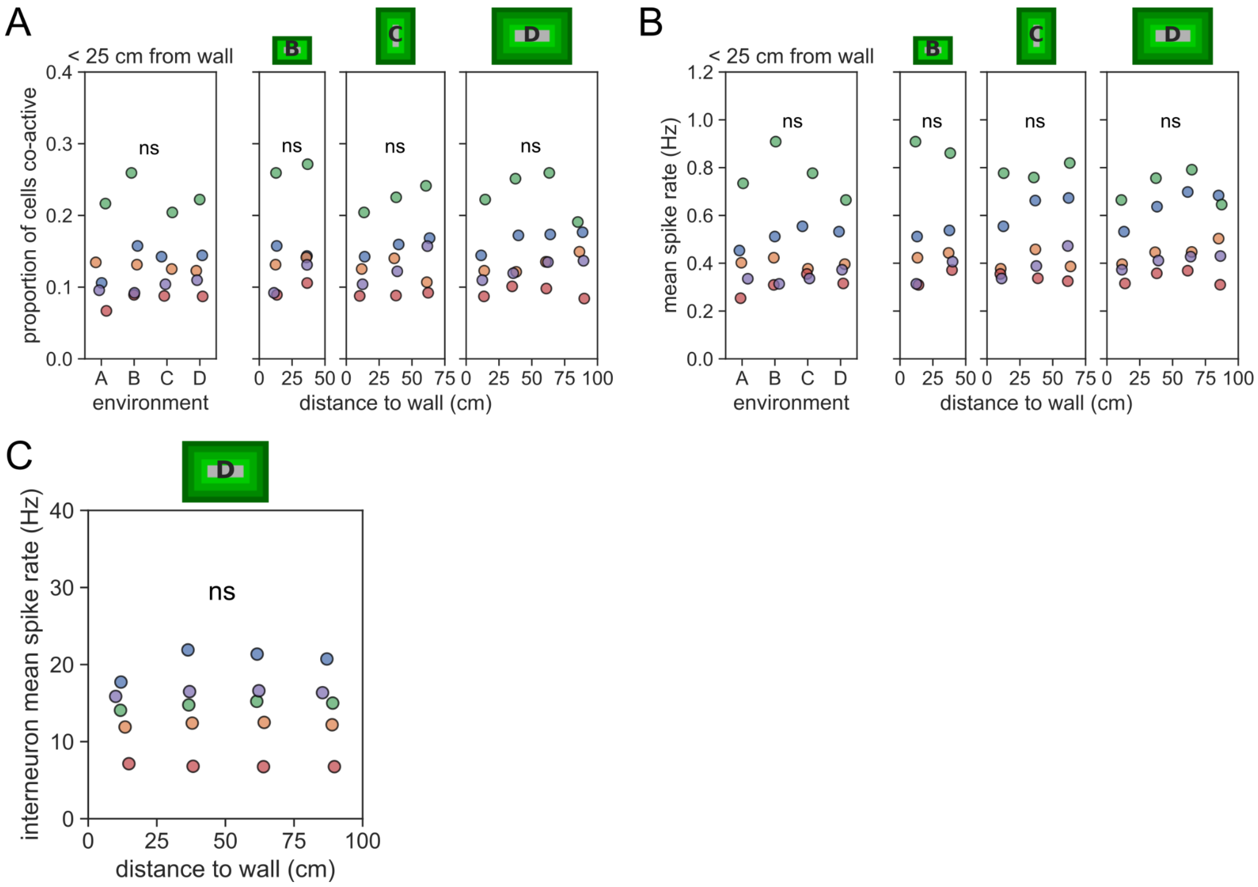

Figure S4. Homeostasis of place cell population activity. Related to main Figure 4. (**A**) The proportion of place cells recorded in each animal that were firing at greater than 1 Hz was constant at different distances to the wall in all environments and same across environments. P-value markers: ns, not significant. (**B**) The mean firing rate of all place cells recorded in each animal was constant at different distances to the wall in all environments and across environments. (**C**) Interneuron firing rate is constant at all distances to walls. Mean spike rate of all interneurons detected in each animal at different distances to wall in environment D.

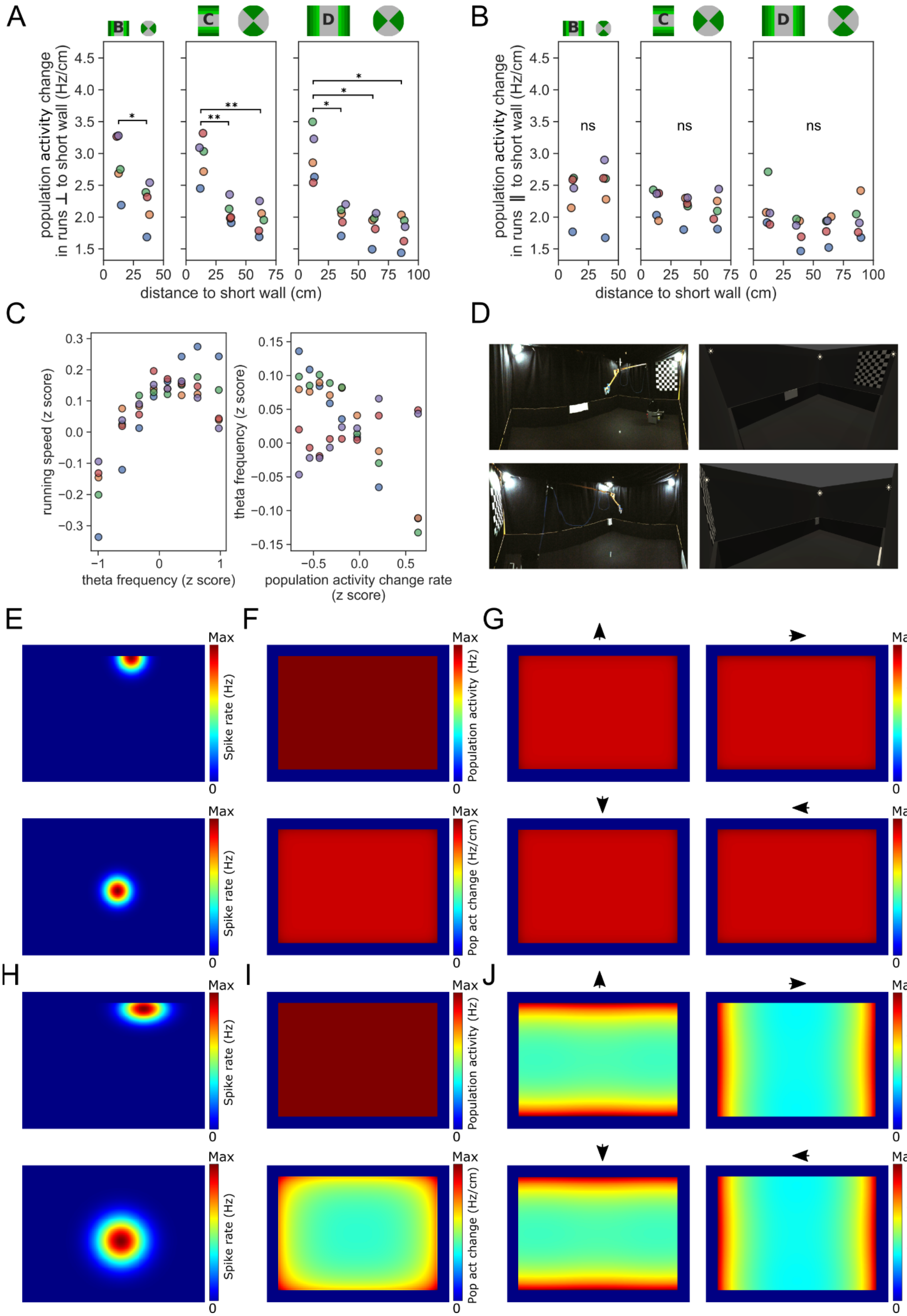
A
B
C
D
population activity change in runs ⊥ to short wall (Hz/cm)
population activity change in runs ∥ to short wall (Hz/cm)
distance to short wall (cm)
ns
running speed (z score)
theta frequency (z score)
population activity change rate (z score)
E
F
G
H
I
J
Max
Spike rate (Hz)
Population activity (Hz)
Pop act change (Hz/cm)

Figure S5. Analysis of population activity change rate. Related to main Figure 5. (**A&B**) Population activity change was higher in runs orthogonal and close to walls in all environments. (**A**) Euclidean distance between vectors representing population activity at different distances to the short walls of each environment, only including runs orthogonal to the short wall. Pair-wise post hoc tests adjusted for multiple comparisons using Benjamini/Hochberg (non-negative) correction. The cartoon above each plot indicates the wall distances and included locations in the environment in green, and the green sectors on the circles indicate the sampled movement directions. P-value markers: *, 0.05; **, 0.01; ns, not significant. (**B**) Same as **A**, but runs parallel to the short wall. (**C**) Running speed (z-scored) was positively correlated with theta frequency (z-scored) (r = 0.558, p = 0.0002) in partial correlation while accounting for population activity change rate (z-scored) in the largest environment, however, theta oscillation frequency was not correlated with population activity change rate, while running speed was accounted for in the partial correlation analysis (r = -0.009, p = 0.96). (**D**) The virtual environment used to calculate visual change. Recording environment D (left) was replicated in a virtual environment (right) in order to estimate each rodent's change in visual scene during its movement through the experimental environment. (**E**) Simulating 1271 equal-sized Gaussian place fields (two examples shown) that evenly span the largest recording environment such that 'true' field peaks can lay outside the environmental boundaries means that (**F** top) the total firing of the place cell population and (**F** bottom) the rate of change in the place cell population are the same at every measurable location, independent of heading direction (**G**). If instead (**H**) the place fields are distributed more densely at boundaries with field widths that increase the further the distance to an orthogonal boundary (two examples shown), we see (**I** top) the total population firing is still the same everywhere but now (**I** bottom) the rate of change in the population activity is increased at the boundaries. In particular, (**J**) this increase at the boundaries is primarily driven when travelling orthogonally to them, as seen in our main results (Fig 5). The simulations can be reproduced using our code shared on GitHub (https://github.com/Barry-lab/Publication_TanniDeCothiBarry2022/tree/main).

Animal	N cells	Mahalanobis distance VS field count	L-ratio VS field count	Mahalanobis distance VS field area	L-ratio VS field area
1	55	r = 0.066, p = 0.630	r = -0.087, p = 0.525	r = -0.079, p = 0.566	r = -0.250, p = 0.065
2	90	r = 0.124, p = 0.245	r = -0.106, p = 0.322	r = -0.002, p = 0.981	r = -0.038, p = 0.722
3	92	r = -0.038, p = 0.719	r = -0.160, p = 0.127	r = -0.026, p = 0.809	r = -0.024, p = 0.822
4	132	r = 0.100, p = 0.254	r = -0.201, **p = 0.021**	r = -0.080, p = 0.361	r = -0.006, p = 0.943
5	132	r = -0.072, p = 0.409	r = -0.050, p = 0.566	r = 0.132, p = 0.130	r = -0.030, p = 0.733

Table S1. Clustering quality does not account for differences in field size and field number between cells. Related to main Figures 2 and 3. Place fields were counted and their average field areas measured for each cell in the largest environment (D), these values were correlated with two measures of cluster goodness - Mahalanobis distance and L-ratio. A single significant correlation was found (in bold), indicating for Animal 4 that better separated cells (L-ratio) tended to have more fields. Since no adjustment for multiple comparisons was applied we believe this single result does not imply a systematic relationship between cluster goodness and field measures.
